# A holistic guide to effective prevention and treatment for kidney stones: a systematic review exploring anti-urolithiasis approaches

**DOI:** 10.1007/s00210-025-04658-y

**Published:** 2025-10-20

**Authors:** Anood T. Allam, Ahmed M. El-Dessouki, Riham A. El-Shiekh, Dina Abou-Hussein, Mahmoud Abdelmouti Mahmoud, Wagih H. Marcus, Hassan A. Ruby

**Affiliations:** 1 Clinical Pharmacy Department at the Faculty of Pharmacy, Cairo University, Cairo, Egypt; 2 Pharmacology and Toxicology Department, Faculty of Pharmacy, Ahram Canadian University, 6Th of October City, Giza, 12566 Egypt; 3 Department of Pharmacognosy, Faculty of Pharmacy, Cairo University, Kasr El-Aini Street, 11562, Cairo, 11562 Egypt; 4 Department of Biochemistry, Faculty of Pharmacy, Cairo University, Cairo, 11562 Egypt; 5 Department of Pharmacology and Toxicology, Faculty of Pharmacy, Cairo University, Cairo, 11562 Egypt

**Keywords:** Urolithiasis, Kidney stones, Nutraceuticals, Recurrence prevention, Urinary health

## Abstract

Urolithiasis, a disease of kidney stones, is a prevalent and recurrent condition characterized by the formation of hard mineral deposits in the urinary tract, leading to significant morbidity and a substantial healthcare burden worldwide. With recurrence rates ranging from 30 to 50% and a lifetime prevalence of 9–12% in industrialized populations, effective prevention and management strategies are crucial. Traditional approaches to urolithiasis, including pharmacological agents and surgical interventions, emphasize medical interventions and lifestyle changes; however, the persistent recurrence and impact on quality of life underscore the necessity for more comprehensive solutions. Recent advancements have focused on the potential of nutraceuticals, including herbal extracts, vitamins, and minerals, as adjuncts in the prevention and treatment of kidney stones. These agents may exert beneficial effects by inhibiting stone crystal formation, modulating urinary pH, reducing urinary calcium and oxalate excretion, enhancing diuresis, and supporting overall renal function. Moreover, these plant-based therapies may help alleviate symptoms such as pain and inflammation related to stone episodes. This systematic review synthesizes findings from 14 randomized controlled trials to assess the therapeutic impact and safety profile of various herbal interventions in patients with diagnosed kidney or ureteral stones. Thus, evidence from these recent clinical trials has increasingly supported the efficacy of single-herb and polyherbal formulations, such as *Nigella sativa*, *Alhagi maurorum*, Subap Plus, and Palasha Kshara, in modulating urinary risk factors and promoting stone expulsion. For instance, these trials demonstrated promising outcomes for several herbs: *Portulaca oleracea* significantly increased urinary citrate and reduced calcium, Traditional Persian Medicine-based Mofatet powder markedly reduced stone size and improved urinary parameters, *Alhagi maurorum* distillates and extracts showed comparable efficacy to hydrochlorothiazide in stone expulsion, and *Nigella sativa* performed as well as or better than tamsulosin in both pain relief and stone passage. Additional polyherbal remedies, such as Subap Plus, Palasha Kshara, and BNO 1040 extract, also yielded favourable results in improving stone clearance, easing symptoms, and potentially preventing recurrence. Notably, most studies reported good short-term tolerability and minimal adverse effects. Despite these promising findings, limitations in sample size, standardization of formulations, lack of comprehensive adverse events reporting, and need for long-term follow-up highlight the necessity for further rigorous trials.

## Introduction

Kidney stone disease (KSD), also known as nephrolithiasis or urolithiasis, refers to the formation of solid crystalline deposits within the kidneys or urinary tract (Xu et al. 2023). These stones arise from the supersaturation and precipitation of urinary solutes, typically under conditions of altered pH, dehydration, or metabolic imbalance. While often presenting with acute pain, the clinical spectrum ranges from incidental findings to recurrent, debilitating episodes that can lead to renal damage (Uribarri 2020; Hoffman et al. 2021).

Kidney stones are heterogeneous in both composition and etiology and are generally classified based on their dominant chemical constituents (Pak et al. 2003). The most common type is calcium oxalate, accounting for approximately 70–80% of all stones (Coe et al. 1992; Pak et al. 2003; Taylor et al. 2005). These may form independently or in combination with calcium phosphate and are often associated with hypercalciuria, hyperoxaluria, or hypocitraturia (Cupisti et al. 2007; Kustov et al. 2016). Uric acid stones, comprising 5–10% of cases, are radiolucent and typically form in acidic urine, particularly in patients with metabolic syndrome, gout, or high-purine diets. Their formation is strongly influenced by low urinary pH, rather than systemic uric acid levels alone (Tasian and Copelovitch 2014; Nevo et al. 2020). Struvite stones, also known as infection stones, are composed of magnesium ammonium phosphate and linked to chronic urinary tract infections by urease-producing bacteria (Das et al. 2017). Cystine stones are rare and result from a genetic defect in amino acid reabsorption, leading to excess urinary cystine. They tend to recur and are often difficult to manage, requiring a combination of hydration, alkalinization, and pharmacological therapy (D’Ambrosio et al. 2022). Other less common types include xanthine, drug-induced stones (e.g. indinavir), and mixed-composition stones, which reflect metabolic or environmental factors (Seegmiller 1968; Daudon and Jungers 2004). Nephrolithiasis, the formation of stones within the renal collecting system, affects approximately 10% of the population over their lifetime and is associated with significant healthcare costs and patient burden (Chewcharat and Curhan 2021). Pathogenesis involves the crystallization of urinary solutes due to supersaturation and impaired inhibitory mechanisms. Despite advances in surgical treatment, recurrence remains a significant concern, shifting the focus toward prevention by elucidating the underlying mechanisms and risk profiles. The aim of the present review is to provide an important assessment of the role of dietary plants as natural supplements in the prevention or management of kidney stones and indicate underlying pharmacological mechanisms.

### Prevalence and demographics

KSD has evolved from an episodic, localized ailment into a pervasive global health challenge, driven by changes in lifestyle, environment, and diagnostic awareness. In the USA, long-term data from the National Health and Nutrition Examination Survey (NHANES) illustrate this shift: early survey cycles relied on a simple “Have you ever passed a stone?” question to capture symptomatic cases, yielding a prevalence of 3.8% between 1976 and 1994. Later NHANES iterations questioned the number of stones passed, distinguishing single events from recurrent or silent (asymptomatic) stones. These refinements coincided with a rise to 5.2% by the mid-1990s and, more recently, to approximately 9.3% between 2007 and 2016 (Stamatelou et al. 2003; Romero et al. 2010; Hyams and Matlaga 2014).

A parallel surge has occurred in Asia’s so-called “stone belt” countries. In China, prevalence climbed steadily after 1978, reaching 5.8% by 2013; in Japan, it roughly doubled to 9% by 2005; South Korea saw rates jump from 3.5% in 1998 to 11.5% in 2013; southern Iran now reports over 21%; and Saudi Arabia experienced a sharp rise from 6.8 to 19.1% over a comparable period. Yet, these figures derive from heterogeneous case-finding methods, self-reported histories prone to recall bias, imaging studies of varying sensitivity, and medical records-based reviews that may overlook silent stones, making direct cross-country comparisons problematic and obscuring the relative impact of dietary, metabolic, genetic, and climatic drivers (Nelli 2004; Edvardsson et al. 2013, 2018; Liu et al. 2018).

Standardizing diagnostics, including imaging thresholds, stone composition, and symptoms, as well as unifying surveillance protocols, are essential for accurately mapping global KSD burden. Harmonized data will reveal regional risk factors and guide targeted prevention, either through hydration education, sodium restriction policies, or heat-dehydration countermeasures, to curb the rising incidence worldwide.

### Health implications

While often perceived as an isolated urological event, KSD carries broad and lasting health implications. The acute phase, marked by severe pain, hematuria, and risk of obstruction, can lead to emergency visits, hospitalizations, and surgical intervention. However, the burden extends well beyond acute management.

Recurrent stone formation is common. Studies indicate that approximately 50% of individuals experience a recurrence within 5 to 10 years after their first kidney stone episode (Shastri et al. 2023). This high relapse rate underscores the need for ongoing preventive strategies.

Emerging evidence suggests that KSD is associated with an increased risk of developing CKD. Stone formers, particularly those with recurrent episodes, are at higher risk for sustained reductions in glomerular filtration rate and elevated serum creatinine levels (Rule et al. 2009). The mechanisms may involve obstructive uropathy, recurrent infections, and direct renal parenchymal injury.

KSD often coexists with systemic metabolic conditions. Obesity, hypertension, diabetes mellitus, and metabolic syndrome are not only risk factors for stone formation but may also be exacerbated by it (Rahman et al. 2021). The interplay between these conditions complicates management and increases long-term morbidity.

Beyond physical health, KSD can significantly influence mental well-being. Recurrent pain episodes, dietary restrictions, and anxiety over potential recurrences contribute to psychological distress, including depression and decreased quality of life (Patel et al. 2017). These effects are particularly pronounced in younger patients and those with frequent stone episodes.

Given its increasing prevalence and systemic associations, KSD should be regarded not merely as a localized urological condition but as a chronic, relapsing disorder with multisystem implications. This perspective necessitates proactive, long-term management strategies that address both the physical and psychosocial dimensions of the disease.

### Economic burden and challenges for global health systems

KSD imposes a significant and growing financial burden on healthcare systems globally. This impact stems from both the acute nature of stone episodes and the chronic risk of recurrence, often requiring repeated diagnostics, surgical procedures, and long-term follow-up. In the USA, annual costs related to KSD are estimated to exceed $5 billion, a figure that has steadily risen alongside increasing incidence and broader utilization of advanced imaging and minimally invasive therapies (Hyams and Matlaga 2014).

Much of the direct cost is driven by emergency department visits for acute renal colic, frequent use of non-contrast CT scans for diagnosis, and interventions such as extracorporeal shock wave lithotripsy (SWL), ureteroscopy (URS), and percutaneous nephrolithotomy (PCNL). A single kidney stone episode can cost between $2000 and $10,000, and up to 20% of patients require repeat interventions within 5 years, significantly compounding long-term expenses (Hyams and Matlaga 2014; Vo et al. 2018).

Indirect costs, including lost productivity, caregiver burden, and reduced quality of life, are substantial. In the USA, indirect costs alone have been estimated to exceed $775 million annually, accounting for approximately 3.1 million lost workdays per year (Saigal et al.^2005^). Mental health impacts and lifestyle disruptions further add to the overall toll.

In low- and middle-income countries, limited access to timely diagnosis and appropriate care frequently results in complications such as obstructive uropathy, significantly increasing long-term costs and disability (Watson et al. 2022). Even in high-income nations, aging populations and rising prevalence among women are escalating healthcare expenditures. For instance, in Germany, kidney stone-related interventions dramatically surged by 270% between 2005 and 2016, markedly increasing per-patient costs (Heers et al. 2022).

Preventive strategies, such as metabolic evaluation, dietary counseling, pharmacological therapy, and telehealth follow-up, have been shown to significantly reduce recurrence and associated long-term costs (Fink et al. 2013). As KSD becomes increasingly prevalent worldwide, investment in preventive and standardized management approaches will be crucial for reducing its economic impact.

### Pathophysiology of kidney stone formation

Kidney stone formation is a dynamic and multifactorial process that reflects a complex interplay between urinary chemistry, renal physiology, genetic factors, and environmental influences. The pathophysiological cascade typically proceeds through several distinct but interrelated stages: supersaturation, nucleation, crystal growth, aggregation, and crystal retention (Table 1) (Evan et al. 2006; Khan 2013; Ryan and Ing 2018).

**Table 1:** Overview of major kidney stone types and their pathophysiological characteristics

Stone type	Composition	Prevalence (%)	Pathophysiological features
Calcium oxalate (CaOx)	Calcium + oxalate	65.9	Forms via free-particle or fixed-particle mechanisms; promoted by hypercalciuria, hyperoxaluria, and low urine volume; strongly linked to Randall’s plaques (Evan et al. 2006; Dawson and Tomson 2012)
Calcium phosphate (CaP)	Carbapatite/brushite	15.6	Develops in alkaline urine; brushite is resistant to fragmentation and linked to papillary damage and recurrent stone formation (Brinkman et al. 2021)
Uric acid	Uric acid	12.4	Arises in acidic urine (pH < 5.5); common in patients with obesity, diabetes, and metabolic syndrome due to impaired ammonia genesis and acid load (Sakhaee 2009; Wang et al. 2021)
Struvite	Magnesium ammonium phosphate	2.7	Infection stones associated with urease-positive bacteria (e.g. *Proteus* spp.); rapid growth forming staghorn calculi (Griffith and Musher, 1976 ; Griffith 1978; Tavichakorntrakool et al. 2012; Arabski et al. 2019 )

#### Supersaturation and nucleation

The initial step in stone formation is urinary supersaturation with lithogenic solutes such as calcium, oxalate, phosphate, uric acid, and cystine. Supersaturation is a thermodynamic condition in which the concentration of these solutes exceeds their solubility product, thus favoring crystallization (Evan et al. 2006; Alexander 2023). The degree of supersaturation determines the risk and rate of stone formation and is influenced by factors such as urine volume, pH, ionic strength, and temperature.

Nucleation occurs when solute molecules aggregate to form crystal nuclei. This can happen spontaneously (homogeneous nucleation) or be catalyzed by preexisting surfaces such as cell debris, organic matrices, or Randall’s plaques (heterogeneous nucleation). Randall’s plaques, which originate in the basement membranes of the thin loops of Henle, are primarily composed of interstitial calcium phosphate deposits that eventually erode into the papillary urothelium and serve as niduses for calcium oxalate overgrowth (Evan et al. 2006; Khan 2013).

#### Crystal growth and aggregation

Once nucleated, crystals grow by accretion of additional ions from the surrounding urine. The rate of growth is enhanced in environments with sustained supersaturation and limited inhibitory activity. Aggregation refers to the clumping of crystals into larger masses, a process influenced by urinary macromolecules, such as Tamm-Horsfall protein (uromodulin), osteopontin, and nephrocalcin. These proteins can act as both promoters and inhibitors of aggregation depending on their concentration, conformational state, and post-translational modifications (Stamatelou et al. 2003; Okumura et al. 2013; Tanaka et al. 2021).

#### Crystal retention and stone formation

Under normal circumstances, formed crystals are flushed out with urine. However, crystal retention may occur through adhesion to the urothelium or entrapment in renal tubular structures. Tubular plugging is a critical mechanism in the pathogenesis of brushite and calcium phosphate stones and has been associated with tubular cell injury, inflammation, and interstitial fibrosis (Evan et al. 2006; Ryan and Ing 2018; Wang et al. 2021).

The retention of crystals and their subsequent growth into clinically significant stones is further facilitated by defects in urine flow dynamics, low levels of natural inhibitors (e.g. citrate, magnesium), and alterations in urinary pH. Acidic urine promotes uric acid and cystine crystallization, while alkaline urine favors calcium phosphate precipitation (Sakhaee 2009; Srivastava and Schwaderer 2009).

#### Molecular and genetic factors

Genetic factors influence stone susceptibility via alterations in urinary solute transport, crystal handling, and inhibitor expression. Mutations in SLC3A1 and SLC7A9 cause cystinuria, while CLDN14 and CASR variants are associated with calcium stone risk (Thorleifsson et al. 2009; Halbritter et al. 2018; Palsson et al. 2019; Howles and Thakker 2020; Halbritter 2021; Singh et al. 2022). Additionally, molecular pathways involving reactive oxygen species, NLRP3 inflammasome activation, and renal tubular cell apoptosis have been implicated in stone pathogenesis (Wang et al. 2022).

### Risk factors for kidney stone formation (Table 2)

#### Non-modifiable risk factors

##### Genetic predisposition

Familial aggregation and Mendelian disorders such as cystinuria, primary hyperoxaluria, and distal renal tubular acidosis are linked to early-onset and recurrent stones (Halbritter et al. 2018; Howles and Thakker 2020; Halbritter 2021; Singh et al. 2022)(Table 2).

**Table 2:** Summary of non-modifiable and modifiable risk factors for kidney stone formation

Risk factor type	Specific risk factor	Pathophysiological impact
Non-modifiable	Genetic mutations (e.g. SLC3A1, SLC7A9, CLDN14)	Affect tubular solute handling and urine composition; increase risk of cystine and calcium stones
	Age and sex	Male predominance: hormonal shifts post-menopause raise risk in women
	Anatomical abnormalities	Medullary sponge kidney and horseshoe kidney promote urinary stasis
	Family history	Strong familial clustering of calcium stone disease
Modifiable	Low fluid intake	Leads to concentrated urine and increased supersaturation
	High sodium diet	Promotes calciuria by impairing calcium reabsorption
	Excessive animal protein	Increases acid load, calcium excretion, and lowers citrate
	High dietary oxalate	Promotes calcium oxalate crystallization
	Obesity and metabolic syndrome	Acidifies urine, promotes uric acid and calcium stones
	Diabetes mellitus	Associated with low urinary pH and hyperuricosuria
	Certain medications (e.g. topiramate, diuretics)	Alter urinary pH and solute excretion

##### Age and sex

Men are more commonly affected, though postmenopausal women experience increased risk due to estrogen decline (Yu and Yin 2017; Goldfarb et al. 2019).

##### Anatomical abnormalities

Congenital anomalies like medullary sponge kidney and horseshoe kidney promote urinary stasis and stone formation (Yohannes and Smith 2002).

#### Modifiable risk factors

##### Low fluid intake

Dehydration concentrates urine, promoting supersaturation. A 24-h urine volume < 2.0 L is associated with increased risk (Jones et al. 2021; Courbebaisse et al. 2023).

##### Dietary factors

High consumption of oxalate (spinach, nuts), sodium, animal protein, and fructose increases lithogenic potential. Conversely, inadequate dietary calcium paradoxically raises risk by allowing more oxalate absorption in the gut (Taylor et al. 2004).

##### Metabolic syndrome

Obesity, insulin resistance, and type 2 diabetes are linked to uric acid stone formation due to impaired ammonium excretion and acidified urine (Curhan et al. 1998; Maalouf 2011; Felizio and Atmoko 2022).

##### Comorbidities

Hyperparathyroidism, IBD, gout, and metabolic acidosis predispose individuals to specific stone types (Schaeffer et al. 2011; Liu et al. 2023b; Sáenz-Medina et al. 2023).

##### Medications

Topiramate, loop diuretics, and antiretroviral therapies can alter urine pH and solute excretion and thus can affect stone formation (Dawson-Hughes et al. 2004; Dawson and Tomson 2012; Dobrek 2020).

#### Lifestyle modifications and dietary prevention

##### Hydration and urine dilution

The cornerstone of stone prevention is maintaining a high urine volume. Clinical guidelines recommend fluid intake to achieve at least 2.5–3.0 L of urine output daily (Borghi et al. 1996). Water is ideal; citrus beverages (e.g. lemonade, orange juice) can increase urinary citrate.

##### Dietary adjustments

- Calcium: Ensuring adequate calcium intake (1000–1200 mg/day from food), which binds dietary oxalate and reduces its absorption (Menon 1993).
- Oxalate: Limiting high-oxalate foods and pairing them with calcium-containing meals (Prezioso et al. 2015).
- Sodium: Restricting to < 2300 mg/day to reduce urinary calcium excretion (Heilberg and Goldfarb 2013).
- Animal protein: Excess promotes uric acid production and reduces citrate; moderate protein intake is the best (Dawson-Hughes et al. 2004; Prezioso et al. 2015).
- Weight management and metabolic optimization: Lifestyle interventions, involving calorie restriction, physical activity, and glycemic control, are essential in reducing stone recurrence, especially in people with risk factors, such as overweight and diabetic individuals (Curhan et al. 1998; Felizio and Atmoko 2022).

### Mechanism of stone formation

The formation of kidney stones involves a complex interplay of genetic, metabolic, and environmental factors. Key processes include supersaturation of urine with stone-forming constituents, nucleation, crystal growth, aggregation, and retention within the kidneys. Understanding the mechanisms behind stone formation can help in developing preventive and therapeutic strategies.

#### Nucleation

The initial and critical stage in kidney stone formation is when free ions in the urine aggregate to form microscopic particles. This process can occur in the renal tubular fluid or on the surfaces of the extracellular matrix and renal cells. Nucleation is driven by the supersaturation of urine with stone-forming constituents such as calcium, oxalate, phosphate, uric acid, or cystine (Aggarwal et al. 2013). There are two types of nucleation: homogeneous and heterogeneous. Homogeneous nucleation occurs in a pure solution without any foreign particles, while heterogeneous nucleation occurs on pre-existing surfaces or particles, such as Randall’s plaques, which are calcium phosphate deposits in the renal papilla (Ratkalkar and Kleinman 2011). The balance between promoters and inhibitors in the urine significantly influences nucleation. Promoters, such as high levels of calcium and oxalate, facilitate nucleation, while inhibitors, such as citrate and magnesium, prevent it by binding to stone-forming ions and reducing their supersaturation (Ratkalkar and Kleinman 2011). Understanding the precise mechanisms of nucleation is essential for developing targeted therapies to prevent kidney stone formation.

#### Crystal growth and aggregation

Once nucleation occurs, the crystals grow and aggregate. During crystal growth, individual crystals increase in size as more stone-forming ions, such as calcium and oxalate, deposit onto the crystal surface. This process is influenced by factors such as urine supersaturation, pH, and the presence of inhibitors and promoters (Aggarwal et al. 2013). Aggregation involves the clustering of these growing crystals into larger conglomerates, which can eventually form a clinically significant stone (Michibata et al. 2024). The presence of macromolecules like osteopontin and Tamm-Horsfall protein in urine can inhibit crystal aggregation by binding to crystal surfaces and preventing them from sticking together (Rimer et al. 2017). Conversely, conditions that reduce the concentration of these inhibitors or increase the concentration of promoters, such as high urinary calcium or oxalate levels, can enhance crystal growth and aggregation.

#### Retention and fixation

Crystals that have formed and grown are retained within the kidney, leading to stone development. This process often involves the attachment of crystals to the renal epithelium or interstitial structures. One common site for crystal retention is Randall’s plaques, which are deposits of calcium phosphate located in the renal papilla (Evan 2010). These plaques serve as a nidus for calcium oxalate crystal attachment and growth. Additionally, crystals can become lodged in the ducts of Bellini, particularly in conditions like hyperoxaluria and distal renal tubular acidosis (Khan et al. 2021). The retention of crystals is facilitated by factors such as renal tubular injury, inflammation, and the presence of specific proteins that promote adhesion (Verkoelen and Verhulst 2007).

#### Further aggregation and secondary nucleation

The retained crystals undergo further aggregation and secondary nucleation, leading to the formation of larger stones. This process is modulated by the balance of promoters and inhibitors in the urine. Long-term accretion of additional elements, both crystalline and organic matrix, contributes to the growth of the clinical stone (Evan et al. 2015).

### Pathways of anti-urolithiasis drugs

Anti-urolithiasis drugs work through various mechanisms to prevent stone formation or promote stone dissolution. These mechanisms can be broadly categorized into the following pathways:

#### Inhibition of crystal nucleation and growth

Inhibition of crystal nucleation and growth is a critical pathway in the prevention of kidney stone formation. Anti-urolithiasis drugs that target this pathway work by reducing the supersaturation of urine with stone-forming constituents, thereby preventing the initial formation of crystals. Citrate is one of the most effective agents in this regard; it binds to calcium ions, reducing their availability to form calcium oxalate and calcium phosphate crystals (Hanchanale et al. 2015). This not only prevents nucleation but also inhibits the growth of any existing crystals by increasing their solubility. Magnesium also plays a significant role by competing with calcium for oxalate binding, thus reducing the formation of calcium oxalate crystals (Allam 2024).

#### Modulation of urine pH and promotion of crystal dissolution

Adjusting urine pH and enhancing crystal dissolution are essential approaches in the treatment of urolithiasis. Alkalinizing agents, such as potassium citrate and sodium bicarbonate, play a pivotal role by increasing the pH of urine, which enhances the solubility of uric acid and cystine stones, making them easier to dissolve (Pinheiro et al. 2013; Segall et al. 2024). This alkalinization process not only helps in dissolving existing stones but also prevents the formation of new ones by maintaining an optimal urinary environment. Thiol-containing drugs, such as tiopronin and D-penicillamine, are particularly effective against cystine stones. These drugs form soluble complexes with cystine, significantly reducing its concentration in the urine and promoting the dissolution of cystine crystals (Dolin et al. 2005; Moussa et al. 2020). By targeting both the dissolution of existing stones and the prevention of new stone formation through pH modulation, these drugs offer a comprehensive approach to managing kidney stones.

#### Reduction of stone-forming substances

This pathway works by decreasing the concentration of key stone-forming ions in the urine, thereby reducing the risk of crystal formation. Thiazide diuretics, for example, are effective in lowering urinary calcium levels by promoting calcium reabsorption in the renal tubules, which decreases calcium supersaturation and the likelihood of calcium stone formation (Li et al. 2020). Allopurinol, another important drug, inhibits xanthine oxidase, an enzyme involved in the production of uric acid. By reducing uric acid levels, allopurinol helps prevent the formation of uric acid stones (Goldfarb et al. 2013). Additionally, dietary modifications and supplements, such as increasing fluid intake and consuming magnesium, can further reduce the concentration of stone-forming substances in the urine.

### Emerging therapies and future directions

Advances in molecular biology and pharmacology have led to the development of novel anti-urolithiasis therapies. Some promising approaches include:

#### MicroRNA-based therapies

miRNAs are small non-coding RNAs that regulate gene expression. Certain miRNAs, such as miR-34a, have been shown to inhibit crystal adhesion and reduce stone formation. Targeting miRNAs offers a novel therapeutic strategy for urolithiasis (Wang et al. 2020).

### Conventional treatments for kidney stones

Conventional treatments for kidney stones are diverse, aiming to alleviate symptoms, remove calculi, and prevent recurrence (Joshi et al. 2021). These treatments are generally categorized into medical management, minimally invasive procedures, and surgical interventions, tailored to the stone’s size, composition, location, and the patient’s clinical profile. Recent advancements have further refined these approaches, improving both patient outcomes and the overall efficiency of treatment protocols. Additionally, public awareness campaigns and preventative measures have gained prominence, addressed the root causes of kidney stones, and reduced their incidence (Kronenberg et al. 2023; Singh et al. 2024; Wang et al. 2025).

### Medical management

Medical management is the first line of treatment for small, non-complicated kidney stones (Jiang et al. 2021). It focuses on promoting spontaneous passage, controlling pain, and preventing further stone formation. This approach is often supported by a combination of pharmacological therapies, lifestyle changes, and dietary interventions. Increasingly, patient education is recognized as a cornerstone of effective management, empowering individuals to make informed decisions about their health (Siener and Metzner 2023; Balawender et al. 2024).

#### Analgesics and symptom control

Pain associated with kidney stones can be debilitating. Nonsteroidal anti-inflammatory drugs (NSAIDs), such as ibuprofen and diclofenac, are commonly used to relieve pain and inflammation. In cases of severe pain, opioids may be prescribed under careful supervision. For patients with contraindications to NSAIDs, acetaminophen may serve as an alternative (Özdemir et al. 2023; Dönmez et al. 2024). Emerging research into novel pain management techniques, including nerve blocks and localized analgesics, is showing promise in providing effective relief. Additionally, non-pharmacological pain management methods, such as heat therapy and mindfulness practices, are being explored as complementary approaches (Chen et al. 2021; Freiwald et al. 2021; Brandel et al. 2022).

#### Medical expulsive therapy (MET)

For stones smaller than 10 mm, medical expulsive therapy (MET) can enhance spontaneous stone passage (Sayed et al. 2022; Song et al. 2024). Alpha-blockers like tamsulosin and calcium channel blockers such as nifedipine relax smooth muscles in the ureter, facilitating stone expulsion. Evidence suggests that MET is particularly effective for distal ureteral stones, with reduced pain episodes and time to stone clearance. Recent meta-analyses have highlighted the importance of patient adherence to MET regimens for optimizing outcomes. Advanced formulations of MET agents with prolonged release are being developed to simplify dosing schedules and improve patient compliance (Cui et al. 2019; Fontenelle and Sarti 2019; Ahmed et al. 2022; Ziaeefar et al. 2023).

#### Urine alkalization

For uric acid or cystine stones, urine alkalization is a key strategy. Potassium citrate or sodium bicarbonate is used to increase urinary pH, dissolve stones, and prevent recurrence. Studies have shown that significant stone dissolution rates occur when urine pH is maintained between 6.5 and 7.0. Advanced formulations of alkalizing agents with extended-release properties are being developed to enhance patient compliance and efficacy. Additionally, combining alkalization with dietary adjustments can further optimize results, highlighting the importance of a holistic approach (Guittet et al. 2020; Xue et al. 2021; Mousavi et al. 2024).

#### Dietary and lifestyle modifications

Medical management includes tailored dietary interventions, such as increased fluid intake to maintain a urine output of at least 2.5 L/day, reduced sodium consumption, and moderate protein intake. Calcium-rich foods are encouraged, while oxalate-rich foods (e.g. spinach and nuts) are limited for patients prone to calcium oxalate stones (Siener 2021; Balawender et al. 2024). Additionally, weight management and regular physical activity are emphasized, as obesity is a recognized risk factor for the formation of stones. Education programs targeting dietary compliance have demonstrated positive impacts on long-term stone prevention. Innovative tools, such as mobile apps for tracking fluid intake and dietary choices, are being adopted to support patients in adhering to their treatment plans (Mao et al. 2022; Aksenov et al. 2024; Katkam and Beddhu 2024).

### Minimally invasive procedures

When stones are too large or refractory to medical management, minimally invasive techniques are employed. These procedures are effective for stones less than 20 mm in size, with minimal morbidity. Advancements in imaging and instrumentation have further improved the precision and success rates of these interventions, making them increasingly accessible and patient-friendly (Cauni et al. 2023; Liu et al. 2023a; Setthawong et al. 2023).

#### Extracorporeal shock wave lithotripsy (ESWL)

ESWL is a non-invasive procedure that uses acoustic shock waves to fragment stones into smaller pieces, which can be passed through the urinary tract. It is particularly effective for renal pelvis and upper ureteral stones. However, its efficacy declines for stones larger than 20 mm or those with high density, as assessed by computed tomography (CT) (Yoon et al. 2021; Setthawong et al. 2023). Post-ESWL complications include hematuria and transient obstruction due to stone fragments. Newer-generation lithotripters with enhanced targeting capabilities are being introduced to improve outcomes and reduce complications. Moreover, combining ESWL with adjunctive medical therapies has been shown to enhance stone clearance rates (Seker et al. 2020; Yuan et al. 2021).

#### Ureteroscopy (URS)

Ureteroscopy is a widely utilized endoscopic procedure for stones in the ureter or kidney. A ureteroscope is inserted through the urethra to access the urinary tract, and stones are fragmented using laser lithotripsy or retrieved with baskets. URS is highly effective for lower ureteral stones, with high stone-free rates and minimal complications (Zheng et al. 2020; Rammah et al. 2025). Innovations such as single-use ureteroscopes and improved laser technologies are expanding the scope of URS, making it suitable for a broader range of stone types and sizes. Furthermore, the development of flexible ureteroscopes has enhanced access to hard-to-reach areas within the kidney, broadening the utility of this technique (Mazzucchi et al. 2022; Juliebø-Jones et al. 2023; Jing et al. 2024).

### Surgical interventions

For large, complex, or recurrent stones, surgical procedures may be necessary. These are typically reserved for cases where other treatments have failed or are contraindicated. Advances in surgical techniques and perioperative care have significantly reduced the risks associated with these interventions. Enhanced recovery protocols are being implemented to optimize patient outcomes and reduce hospital stays (Cassell III et al., 2020, Lei et al. 2023).

#### Percutaneous nephrolithotomy (PCNL)

PCNL is the gold standard for treating large stones (> 20 mm) or staghorn calculi (De Lorenzis et al. 2022). It involves creating a small incision in the patient’s back to access the kidney, followed by fragmentation and removal of stones using ultrasonic or laser devices. PCNL is associated with high stone clearance rates but carries risks, such as bleeding, infection, and injury to surrounding structures (Qin et al. 2022; Escobar Monroy et al. 2025). Recent innovations, such as miniaturized PCNL instruments and robotic-assisted techniques, have improved patient safety and recovery times (DiBianco and Ghani 2021; Zhu et al. 2023). Additionally, new imaging technologies, such as 3D navigation systems, are enhancing the precision of PCNL procedures (Bouchalakis et al. 2024).

#### Open and laparoscopic surgery

While rare due to advances in minimally invasive techniques, open or laparoscopic surgery may be necessary for exceptionally large or anatomically challenging stones. These approaches provide direct access to the kidney or ureter, ensuring complete stone removal (Paludo et al. 2020). Enhanced imaging systems and robotic platforms are increasingly being integrated into these surgeries, offering greater precision and reduced morbidity. Training programs focusing on laparoscopic techniques are equipping surgeons with the skills to handle complex cases more effectively (Güler et al. 2020; Abdel Raheem et al. 2021; Hasan et al. 2023).

### Adjunctive and post-treatment measures

Effective management of kidney stones extends beyond initial treatment, requiring measures to minimize recurrence. Post-treatment care is crucial to ensure long-term success and patient satisfaction. Integrating digital health tools, such as remote monitoring and telemedicine consultations, is making follow-up care more accessible and efficient (Talyshinskii et al. 2024; Ungerer et al. 2024).

#### Metabolic evaluation

A detailed metabolic evaluation, including urine and serum analysis, helps identify underlying abnormalities such as hypercalciuria, hyperoxaluria, or hypocitraturia. This informs targeted preventive strategies. Emerging diagnostic tools, such as genetic testing and advanced metabolic panels, are enhancing the ability to personalize treatment plans. Collaborations between nephrologists and dietitians are further refining the process of developing customized management strategies for patients (Gambaro et al. 2016; Ranjan et al. 2023; Shastri et al. 2023; Breeggemann et al. 2024).

#### Pharmacological prevention

Patients at high risk of recurrence may benefit from pharmacological agents, such as thiazide diuretics for calcium stones, allopurinol for uric acid stones, or potassium citrate for hypocitraturia. Recent studies have explored the role of novel pharmacological compounds and combination therapies in reducing stone recurrence rates (Adomako and Moe 2020; Bargagli et al. 2024; Cao et al. 2024; Leslie and Bashir 2024).

#### Follow-up imaging

Periodic imaging, using ultrasound or CT scans, is recommended to monitor for residual fragments or new formation of the stones, ensuring timely intervention if necessary. Advances in imaging technology, including low-dose CT scans, are reducing radiation exposure while maintaining diagnostic accuracy. The integration of AI algorithms into imaging systems is aiding in the early detection of recurrent stones and eventually improving patient outcomes (Rodger et al. 2018; Caglayan et al. 2022).

### Clinical review on anti-urolithiasis phytotherapy

Kidney stones, or urolithiasis, represent a significant health challenge globally, affecting a considerable portion of the population (Alirezaei et al. 2023). Statistics propose that between 5 and 15% of people worldwide will experience kidney stones at some point in their lives (Mehrabi et al. 2023), with reported occurrence rates reaching approximately 12% (Alirezaei et al. 2023). The prevalence of this condition has also witnessed a remarkable increase in recent decades (Ansari et al. 2024). Patients often suffer from severe pain and complications such as infection, obstruction, and potential kidney damage (Mehrabi et al. 2023). While conventional medical and surgical interventions like lithotripsy and ureteroscopy are available for stone management, they can be invasive or come with potential adverse events (Alirezaei et al. 2023). Additionally, a significant concern is the increased rate of stone recurrence, which can be as high as 50% within 5–10 years and 75% within 20 years (Ansari et al. 2024), highlighting the need for effective preventive and management strategies that minimize side effects (Mehrabi et al. 2023).

There is a growing global interest in exploring complementary and alternative medicine approaches, particularly phytotherapy or the use of herbal medicines, for managing kidney stones, to account for the limitations and challenges associated with the conventional treatments (Ansari et al. 2024). Traditional medicine systems, such as Traditional Persian Medicine (TPM) and Ayurveda, have long utilized various plants for addressing urinary tract disorders, including kidney stones (Ansari et al. 2024). Besides, herbal preparations are often perceived as having fewer side effects compared to synthetic drugs (Samandarian et al. 2023). However, the efficacy and safety of many traditional remedies require rigorous scientific investigation (Ardakani Movaghati et al. 2019).

Phytotherapy for renal calculi management is thought to exert its effects through various mechanisms. One key modality involves modulating urine composition to prevent stone formation or inhibit crystal growth. This can include increasing levels of inhibitors like citrate, which binds to calcium and prevents crystallization, or decreasing the concentration of stone-forming substances such as calcium or oxalate in the urine (Nisa and Astana 2019). Another mechanism involves decreasing the size or number of existing stones, potentially through dissolution (Ardakani Movaghati et al. 2019). Certain herbal compounds are also believed to help break down or reduce the physical dimensions of stones (Ansari et al. 2024). Furthermore, some plants act by decreasing stone number through promoting stone expulsion from the urinary tract (Aryaeefar et al. 2022). This expulsion effect can be facilitated by diuretic properties that increase urine flow, helping to flush out stones or fragments, or by antispasmodic effects that relax the smooth muscles of the ureter, allowing for easier passage. These various mechanisms of action may work synergistically to prevent, manage, and promote the clearance of kidney stones (Nisa and Astana 2019).

Numerous medicinal plants have been traditionally used and scientifically investigated for their potential in treating urolithiasis. Examples of widely studied herbs with reported anti-urolithic activity include *Nigella sativa* (black seed) (Shakeri et al. 2021), *Alhagi maurorum* (Aryaeefar et al. 2022), *Didymocarpus pedicellata* (often associated with the polyherbal formulation Cystone) (Nisa and Astana 2019), *Peganum harmala* (Shakeri et al. 2020), *Portulaca oleracea* (purslane) (Alirezaei et al. 2023), and *Tribulus terrestris* (Samandarian et al. 2023). Other plants mentioned for their potential include *Phyllanthus niruri*, *Strobilanthes crispa*, *Orthosiphon stamineus*, *Sonchus arvensis*, *Imperata cylindrica*, *Curcuma longa*, *Curcuma xanthorrhiza*, *Adiantum capillus-veneris*, *Stigma maydis* (corn silk), *Cucumis melo*, *Crateva nurvala*, *Musa x paradisiaca*, *Achyranthes aspera*, and *Hordeum vulgare* (Samandarian et al. 2023).

This systematic review aims to explore these therapeutic actions in detail and provide a comprehensive summary of findings from 14 clinical trials published within the last 10 years studying the efficacy and safety of various herbal interventions for kidney stone management. This review seeks to provide evidence-based insights into the role of phytotherapy as a potential alternative or complementary treatment strategy for patients with urolithiasis by analyzing the design, outcomes, and reported efficacy of these studies.

## Methodology

This systematic review was designed to investigate and summarize the clinical evidence surrounding the use of herbal remedies in the prevention and treatment of renal calculi. The review focused on evaluating randomized controlled trials that examined the effectiveness of various natural preparations, whether single-herb or polyherbal formulations, in reducing stone size, number, or recurrence. The work was conducted following the PRISMA 2020 guidelines for transparent and comprehensive reporting of systematic reviews. Additionally, the review process was guided by the methodological principles outlined in the Cochrane Handbook for Systematic Reviews of Interventions, ensuring a rigorous and standardized approach to literature search, study selection, data extraction, and quality assessment. The review also aimed to bridge clinical observations with underlying mechanisms of herbal action, offering an evidence-based perspective on the therapeutic value of phytotherapy in urolithiasis management.

### Search strategy

A comprehensive and systematic search strategy was established to identify relevant clinical trials studying the use of phytotherapy in the prevention and treatment of kidney stones. The search was conducted across multiple internationally recognized databases and clinical trial registries to ensure extensive coverage of the available literature. Specifically, the electronic databases searched included PubMed, Embase, Web of Science (WOS), and Cochrane Central Register of Controlled Trials (CENTRAL) to ensure the retrieval of high-quality, peer-reviewed, and clinically relevant evidence. These databases were chosen because they collectively provide robust reporting of biomedical literature, clinical pharmacology, traditional medicine, and evidence-based interventions. Besides, two major clinical trial registries, ClinicalTrials.gov and the World Health Organization International Clinical Trials Registry Platform (ICTRP), were searched to capture ongoing or recently completed but unpublished trials, ensuring that our review incorporates the most recent studies, both published and grey literature, with minimal publication bias.

The search terms were carefully organized around three conceptual domains: the condition, the intervention, and the study design. For the condition domain, terms such as “kidney stones,” “renal calculi,” “nephrolithiasis,” “urolithiasis,” and “ureteral stones” were used to capture the spectrum of kidney/urinary stone disease. While in the intervention domain, the strategy incorporated a wide range of descriptors including “phytotherapy,” “herbal medicine,” “natural remedies,” “plant extract,” “medicinal plants,” and “anti-urolithic.” These terms were selected to reflect both traditional and modern terminology used in the context of herbal interventions. Finally, the third domain targeted the study design, with terms such as “randomized controlled trial,” “RCT,” “clinical trial,” “interventional study,” and “prospective study” used to focus the search on high-quality clinical evidence. The final search strategy was constructed around those three key concepts: the disease condition (kidney stones), the intervention (herbal or phytotherapeutic agents), and the study design (clinical trials), as the following string: (“kidney stones” OR “renal calculi” OR “nephrolithiasis” OR “urolithiasis”) AND (“herbal medicine” OR “phytotherapy” OR “plant-based remedies” OR “natural remedies” OR “antiurolithiasis” OR “herbal extract” OR “medicinal plants”) AND (“randomized controlled trial” OR RCT OR “clinical trial” OR “controlled study” OR “interventional study”). All search queries were run using this strategy across the aforementioned databases without major modifications, when possible, to improve retrieval cohesiveness and sensitivity.

These terms, as shown, were finally systematically combined using Boolean operators (AND, OR) to structure the search logic. For example, condition-related terms were grouped with OR, intervention-related terms were grouped with OR, and these blocks were then connected with AND to ensure that each result addressed both the condition and the intervention of interest. We also made use of truncation and word variation techniques (e.g. using the asterisk symbol “*”) to capture multiple forms of a root word, such as “ureter*” to include both “ureteral” and “ureteric.” Additional filters were applied to limit the results to studies conducted in human subjects, published in the English language, and designed as clinical trials. The search was further restricted to studies published within the last 10 years to capture contemporary evidence relevant to current clinical practice. The full search strings used for each database, including combinations of MeSH terms and keywords, the final detailed search strategy, and database-specific considerations/filters, are documented in detail and included in the supplementary appendix for transparency and reproducibility. This structured approach ensured that the search was both comprehensive and reproducible, allowing for the identification of all potentially relevant studies for inclusion in the review.

### Eligibility criteria

A set of eligibility criteria was carefully developed before initiating the screening process, based on the internationally recognized PICOS framework: Population (P), Intervention (I), Comparator (C), Outcomes (O), and Study Design (S), to ensure the clinical relevance and methodological robustness of this review. These criteria were designed to guide the identification of studies most likely to provide reliable and applicable evidence on the role of herbal therapies in the prevention and treatment of kidney stones.

The population of interest included human participants of any age or sex who had a confirmed diagnosis of kidney or ureteral stones. Acceptable diagnostic methods included standard imaging modalities such as ultrasound, computed tomography (CT), X-ray of the kidneys, ureters, and bladder (KUB), or intravenous pyelography (IVP). Studies that did not explicitly report diagnostic confirmation using clinical imaging were excluded, as were those conducted only on animals or using in vitro models, due to concerns regarding translational relevance and physiological differences across species.

The intervention component of the PICOS model focused on the use of herbal therapies. Included studies had to evaluate either single-herb or polyherbal preparations, administered to manage renal stones through either dissolving existing stones, promoting their expulsion, or preventing their recurrence. These herbal products could be provided in various traditional or modern forms, such as capsules, decoctions, extracts, or powders, but had to contain whole plant material or crude herbal formulations. Trials involving isolated phytochemicals, synthetic agents, vitamin–mineral supplements, or dietary-only interventions were excluded in order to retain a clear focus on phytotherapeutic practices consistent with integrative and traditional medicine.

The eligible studies had to include a control group to allow for a fair comparison of treatment effects. Acceptable comparators included placebo, standard pharmacological regimens (such as potassium citrate, tamsulosin, or thiazide diuretics), no treatment, or a different herbal remedy. Studies lacking a comparator arm, including quasi-experimental studies (e.g. uncontrolled before-and-after studies) or observational studies (e.g. case reports, case series, or cross-sectional studies), were excluded due to their limited capacity to isolate the effect of the intervention and to minimize bias.

In terms of outcomes, included studies were required to report at least one clinically meaningful endpoint related to the burden or management of kidney stones. These outcomes could include quantitative changes in stone size or number, the rate or time of stone expulsion, recurrence rates, or changes in urinary composition (e.g. oxalate, citrate, calcium, pH). Studies that measured only surrogate endpoints, non-clinical biomarkers, or theoretical mechanisms without patient-relevant clinical outcomes were excluded. Moreover, trials that reported outcomes descriptively without presenting extractable statistical data, such as means, standard deviations, effect size percentages, or sample sizes, were excluded from the meta-analysis, although they were considered for qualitative synthesis if they offered relevant mechanistic insights.

Finally, only randomized controlled trials or prospective interventional studies published in peer-reviewed journals were included to ensure the quality and applicability of the evidence. Eligible studies had to be published in the English language and fall within the time range of January 2015 to March 2025. This filter was applied to capture recent developments in herbal medicine and ensure alignment with current clinical practices. In addition, grey literature, preprints, and studies published in abstract-only form were excluded due to the lack of peer review and limited methodological transparency. These eligibility criteria were carefully applied during the screening process to ensure that the included studies were directly relevant to the clinical question and capable of contributing meaningful data to both the qualitative synthesis and quantitative meta-analysis to achieve the review’s objective of evaluating the real-world utility and effectiveness of phytotherapy in kidney stone management, and the full detailed eligibility criteria is provided in the supplementary materials.

### Screening and study selection

Following the execution of the search strategy across four major databases and two clinical trial registries from 18th February 2025 to 3rd March 2025, all retrieved references were exported as “RIS” files and then combined and imported into Rayyan QCRI, a specialized online platform designed to facilitate systematic review screening. This platform allowed for blinded, independent screening of titles and abstracts by two reviewers, which ensured an unbiased assessment of eligibility and reduced selection bias during the initial phase.

A total of 546 records were identified through database and registry searches before screening, with individual inputs from PubMed (*n* = 24), Cochrane CENTRAL (*n* = 31), Web of Science (*n* = 23), Embase (*n* = 466), ClinicalTrials.gov (*n* = 1), and the WHO International Clinical Trials Registry Platform (ICTRP) (*n* = 1). These records were first filtered to remove non-relevant entries based on the pre-determined limits: 223 records published before January 2015, and 11 non-English language records were excluded. This left 310 records for the title and abstract screening stage.

During the title and abstract screening phase, each citation was independently reviewed by two researchers to determine its potential relevance based on the predefined eligibility criteria. In this phase, 280 records were excluded for the following reasons: 22 were duplicates, 101 involved non-human populations (animal models or cell lines), 123 were of an ineligible study design (such as review articles, observational cohort or case–control studies, cross-sectional studies, case series, and experimental pharmacokinetic or formulation quality assessments), and 34 were found to be out of scope, often discussing other nephro-/uro-logical disorders or unrelated herbal applications.

Following this stage, 30 full-text articles were subjected to a detailed eligibility assessment. That full-text screening was again conducted in duplicate by two independent reviewers, with discrepancies resolved through discussion and, when necessary, by consultation with a third expert. Sixteen papers were then removed from this collection, mostly because their methodology or content did not match the review’s goals. In particular, 3 studies were excluded due to ineligible populations (patients with unrelated comorbid conditions), 3 due to interventions that were either Ayurvedic, non-plant-based, or pharmacological, and 3 due to reporting outcomes outside the scope of urolithiasis (e.g. urinary tract infections, oxalosis, cystinuria). Additional exclusions included 2 trials with unavailable full-text results despite registration and completion, 2 studies using inappropriate comparators, and 1 record published only as a conference abstract with no accessible data.

**Fig. 1:**
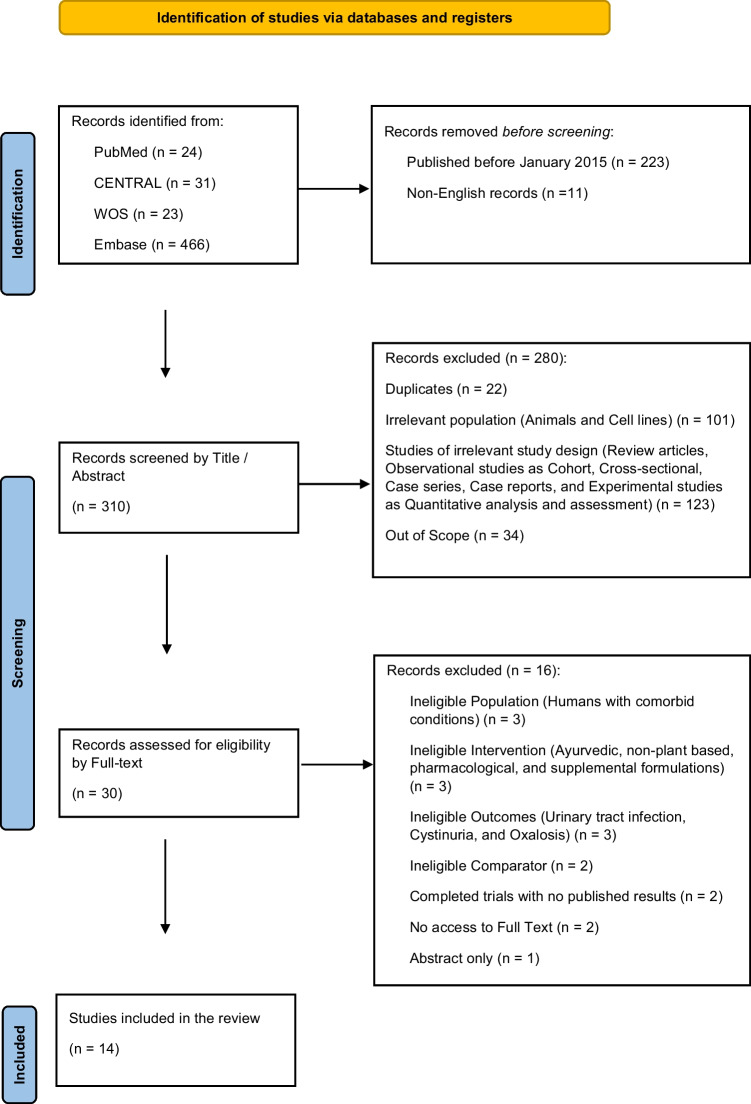
PRISMA flow diagram

Finally, 14 studies met all inclusion criteria and were considered suitable for both qualitative synthesis and, where applicable, quantitative meta-analysis. These studies formed the final evidence base of this literature and were comprehensively evaluated in terms of population characteristics, herbal interventions, outcome measures, and methodological quality. The complete screening process, including the rationale for study inclusion and exclusion, is visually represented in the following PRISMA 2020 flow diagram Fig. 1, ensuring full transparency and adherence to best reporting practices in systematic reviews.

### Data extraction

Data extraction was accomplished using a customized spreadsheet designed in Microsoft Excel 365, tailored specifically to capture the most relevant methodological and outcome-related information across all included trials. The extraction process was carried out systematically by two independent reviewers to ensure accuracy and completeness. Discrepancies were resolved through mutual discussion or, when necessary, by consulting a third expert.

Each study was thoroughly scanned, and the following details were documented: the study identifier, including first author, year of publication, and trial registration ID (if available), along with the primary aim or objective of the trial. The country of origin and, where available, the type of clinical setting (e.g. outpatient clinics, urology centres, academic hospitals) were also noted to provide context for the study environment. In addition, details on the study design, such as whether the trial was randomized, double-blind, single-blind, or open-label, were extracted, as were data on the total duration of the study, including any specific follow-up periods.

Furthermore, patient demographics and sample size distribution were documented, including the total number of participants, the allocation across treatment arms, and dropout or attrition rates. This data was critical for assessing the study power and interpreting the robustness of the reported outcomes. A comprehensive profile of the intervention arm was also noted, including the botanical composition of the herbal product, its formulation (e.g. capsule, decoction, extract), the dosage regimen, route of administration, and, when reported, methods of preparation or standardization. Similarly, parallel data were recorded for the comparator arm, which could include placebo, no treatment, standard pharmacological therapy, or another herbal regimen, with equal attention given to dosing and delivery method.

Moreover, the inclusion and exclusion criteria used by each study for recruiting patients were extracted to understand the context in which these trials were conducted. This helped evaluate population homogeneity and assess how generally the findings might be applicable. Detailed information was also collected regarding the outcomes measured, including stone size, stone number, stone expulsion rates, recurrence, and changes in urinary biochemical markers. For each outcome, data on measurement methods, assessment timepoints, and units of reporting were captured to allow for consistent comparison across studies.

Whenever available, the numerical results were extracted directly, including mean differences, standard deviations, confidence intervals, and *P*-values. These statistical parameters were essential for the subsequent meta-analysis, which was limited to trials that reported quantifiable and standardized outcome data. The conclusion of each study, along with the authors’ reported assessments of safety and efficacy, was also summarized to help put the findings in the larger clinical context.

Apart from these fundamental variables, studies were grouped based on thematic mechanistic classifications, which included treatments meant to alter the composition of the urine to stop stones from forming, dissolve calculi that already existed, or encourage the expulsion of stones by diuretic or antispasmodic effects. Herbal regimens were further categorized based on whether they involved single-herb or polyherbal formulations to investigate if therapeutic complexity affected results. Altogether, this structured and thorough data extraction approach ensured that the review included not only numerical outcomes for meta-analysis but also contextual and mechanistic insights that could facilitate a deeper qualitative synthesis of findings.

### Qualitative data synthesis

A structured qualitative synthesis was conducted in addition to numerical data pooling using meta-analysis to further understand the mechanistic diversity and formulation complexity of herbal therapies used in kidney stone treatment. We aimed to classify and interpret these effects in a systematic manner because we knew that phytotherapeutic interventions frequently work through a variety of mechanisms, from biochemical regulation to physical facilitation of stone expulsion.

The first level of synthesis focused on the formulation type. Interventions were initially classified into two broad groups: single-herb formulations, which rely on the activity of a single plant species, and polyherbal formulations, which combine multiple botanicals with complementary or synergistic mechanisms of action. This classification was particularly important because polyherbal combinations, often grounded in traditional medical systems such as Ayurveda, Persian medicine, or Unani, may exert a multifaceted effect that cannot be attributed to a single active compound. On the other hand, single-herb therapies allow for a clearer mechanistic attribution and standardization, making them easier to interpret pharmacologically. These two formulation types were therefore studied to explore whether patterns emerged in terms of efficacy, safety, or therapeutic target.

The second level of synthesis involved thematic classification based on the proposed or observed mechanisms of action. Those key biological processes that the herbal intervention was targeting, as stated by the authors or deduced from result measures and underlying reasoning, were thoroughly analyzed for each included study. This thematic framework led to the organization of studies into three mechanistic categories:

- Urinary composition modulation and stone formation prevention: This group included studies where the primary goal was to alter the biochemical environment of the urine in ways that would reduce the risk of stone formation. Interventions in this category aimed to increase urinary citrate levels, reduce urinary oxalate or calcium excretion, or improve urine pH balance. Such effects were often assessed via 24-h urine profiles and serum chemistry panels. The objective of these interventions was not necessarily to treat existing stones but to prevent their development or recurrence, a strategy aligned with long-term disease control.
- Stone dissolution and size reduction: Studies in this category focused on the capacity of the phytotherapeutic agent to chemically or enzymatically disintegrate stones that had already formed. Outcomes in these trials typically included reductions in stone diameter, volume, or surface area as measured by imaging techniques. This category was particularly relevant for therapies positioned as alternatives to shockwave lithotripsy or surgical removal and included both single-plant extracts and multi-component formulas with known litholytic properties.
- Stone number reduction via expulsion, diuretic, or antispasmodic effects: This group contained trials in which the main effect of the intervention was to facilitate the passage of stones through the urinary tract. Mechanisms included enhanced urine flow (diuresis), relaxation of ureteral smooth muscle (antispasmodic action), or reduction of edema and inflammation surrounding obstructing stones. These studies typically measured endpoints such as stone expulsion rate, number of stones passed, or reduction in stone burden. Many of the polyherbal formulations in this category drew upon traditional ethnomedicinal combinations known for their “stone-flushing” or urinary cleansing effects.

We were able to construct a mechanism-oriented representation of phytotherapeutic activity in urolithiasis by using this thematic classification for the extracted data. This approach enabled not only a deeper understanding of how herbal remedies might contribute to stone management but also provided a framework to compare their potential therapeutic roles across different stages of the disease, from prevention to active treatment and symptomatic relief.

### Risk of bias assessment

**Fig. 2:**
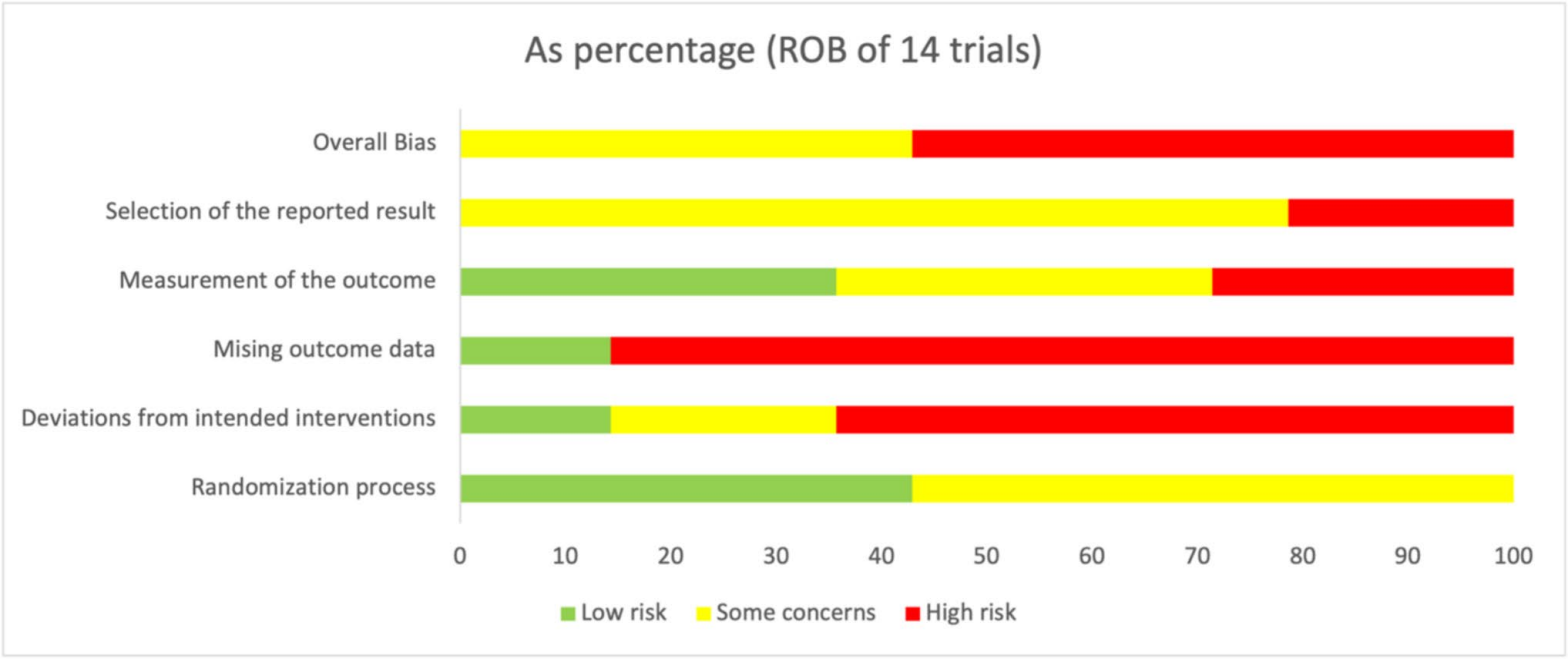
Risk of bias assessment as percentages

**Fig. 3:**
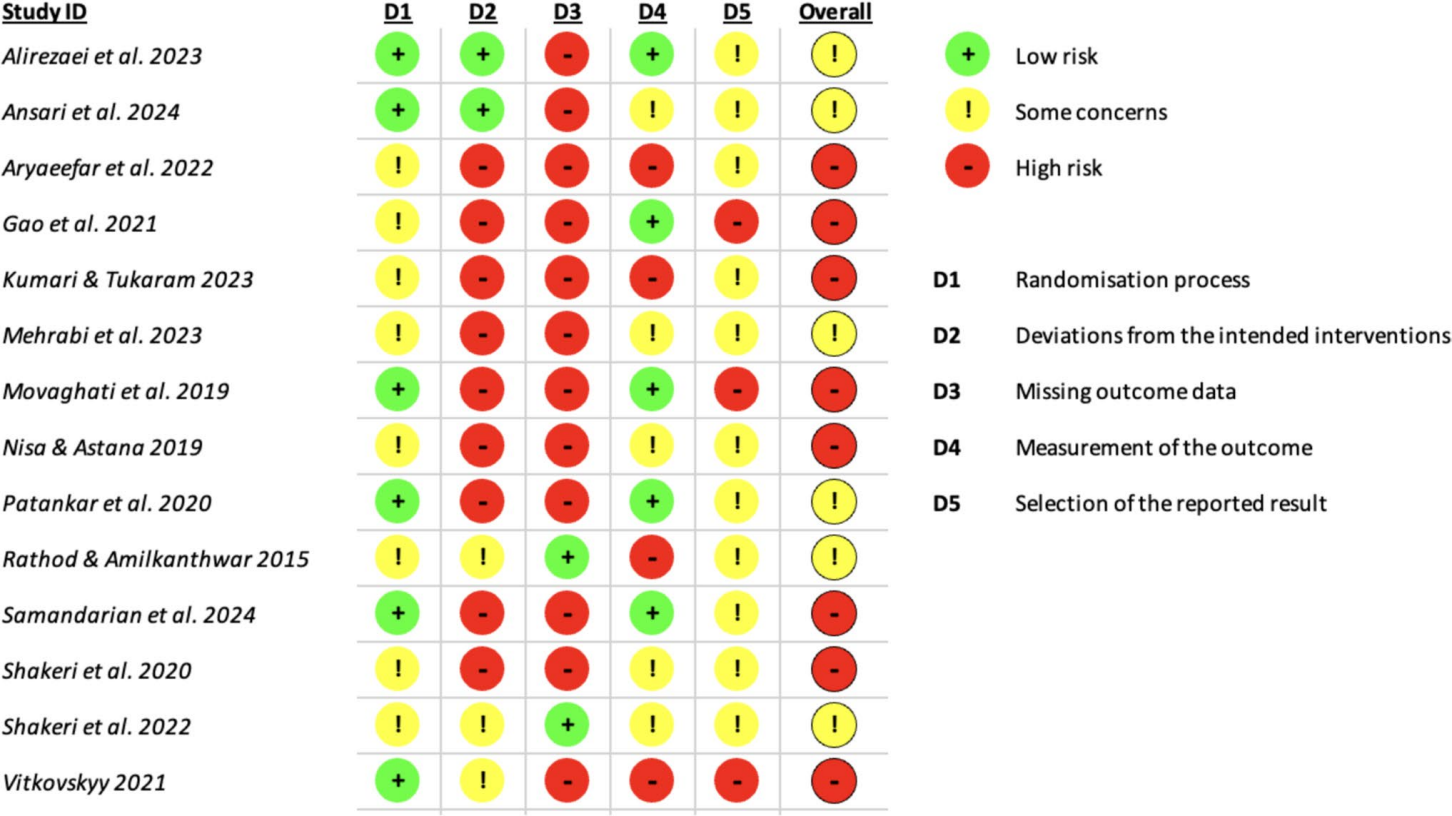
Risk of bias assessment domains’ judgement (for 14 trials)

The Cochrane Risk of Bias 2.0 (RoB 2) tool was employed to ensure a rigorous appraisal of the internal validity of the included randomized controlled trials (RCTs). This tool, grounded in the methodological standards outlined by the Cochrane Collaboration, evaluates potential sources of bias across five distinct domains: bias arising from the randomization process, deviations from intended interventions, missing outcome data, measurement of the outcome, and selection of the reported result. It is specifically designed to assess bias at the outcome level rather than across an entire study, allowing for a more targeted evaluation. The assessment was conducted using the RoB 2.0 beta macro-enabled Excel tool, following the structured signalling questions and algorithmic guidance provided by the Cochrane ROB_2 Crib sheet. This tool facilitates standardized judgments by guiding reviewers through domain-specific questions, eventually categorizing each domain as “low risk,” “some concerns,” or “high risk” of bias. The Excel version further supports transparency and replicability by generating visual risk-of-bias summaries (Figs. 2 and 3) suitable for systematic review reporting.

Across the 14 included studies, the domain-specific judgments revealed substantial heterogeneity in methodological quality. For the randomization process, 42.9% of studies were rated as having low risk of bias, whereas 57.1% raised some concerns, mainly due to insufficient reporting on allocation concealment or sequence generation methods. The domain of deviations from intended interventions showed higher methodological weaknesses, with 64.3% of studies classified as high risk, often due to inadequate blinding or protocol deviations not addressed by intention-to-treat analysis. Missing outcome data was the most problematic domain, with 85.7% of studies judged as high risk, typically due to incomplete follow-up, attrition without explanation, or differential loss to follow-up between arms. Outcome measurement raised a mix of concerns: 35.7% of trials were at low risk, while 35.7% had some concerns, and 28.6% were at high risk, mainly due to lack of blinding of outcome assessors or use of subjective outcome measures without validated tools. Finally, for the domain of selection of the reported result, while only 21.4% of studies were rated as high risk, the majority (78.6%) were found to have some concerns, often due to the absence of trial registration or incomplete reporting of pre-specified outcomes.

Overall, only 42.9% of studies were rated with “some concerns,” while 57.1% were at high risk of bias when aggregating across domains, as illustrated in Table 3. These findings emphasize the importance of cautious interpretation of pooled results, as many included trials were limited by methodological shortcomings that could influence outcome reliability.

**Table 3:** Included trials’ risk of bias assessment percentages

Total number of studies = 14	**Randomization process**	**Deviations from intended interventions**	**Missing outcome data**	**Measurement of the outcome**	**Selection of the reported result**	**Overall bias**
*Low*	42.9	14.3	14.3	35.7	0	0
*Some concerns*	57.1	21.4	0	35.7	78.6	42.9
*High*	0	64.3	85.7	28.6	21.4	57.1

### Summary of findings tables

Two structured summary of findings (SoF) tables (Tables 4 and 5) were created to improve the review’s conclusions’ interpretability and clarity. One table summarized the findings from the included clinical trials, while the second table synthesized data from the natural treatments themselves. These tables were created to combine the most important and clinically useful information from the included material in order to facilitate quick reference by physicians, researchers, and policymakers.

**Table 4:** Natural remedies in kidney stones management: summary of clinical evidence

Plant name	Mechanism(s) of action	Reported clinical outcomes	Reference
*Portulaca oleracea*	Increase urine citrate, decrease urine calcium; potential antioxidant, anti-inflammatory, muscle-relaxant effects	Significant increase in urine citrate and decrease in urine calcium levels; no significant effect on urine creatinine; no significant adverse events	Alirezaei et al. (2023)
*Alhagi maurorum*	Diuretic, spasmolytic, relaxation effect, antioxidant flavonoids, contains alkaloids	Sooner stone removal; high stone expulsion rate (67% at 3 weeks); effective as hydrochlorothiazide in reducing stone size/number (up to 70% diminished); improved pain; no significant side effects	Aryaeefar et al. (2022), Mehrabi et al. (2023)
*Nigella sativa*	Stone dissolution/size reduction, stone expulsion, diuretic, anti-pain; may reduce crystal formation/oxalate, inhibit crystal growth, antioxidant	Significant positive effects on disappearance or reduction of stone size; reduction in stone size and number comparable to tamsulosin; more substantial stone passage; significantly better pain control	Ardakani Movaghati et al. (2019), Shakeri et al. (2021)
*Peganum harmala*	Analgesic properties, contains alkaloids (quinazoline, beta-carboline)	Decreased urinary stone size and numbers comparable to tamsulosin; more significant decrease in pain score; efficacy comparable to tamsulosin; no significant side effects	Shakeri et al. (2020)
*Cucumis sativus*, *Cucurbita pepo*, *Apium graveolens*, *Foeniculum vulgare*, *Pimpinella anisum*,* Tribulus terrestris*	Traditional Persian Medicine formulation; decreased urinary calcium, increased urinary magnesium	Significant 60.73% decrease in stone size; effective in reducing calcium kidney stone size; no potential nephro/hepatotoxicity	Ansari et al. (2024)
*S. crispa*, *O. stamineus*, *S. arvensis*, *I. cylindrica*, *C. longa*, *C. xanthorrhiza*,* P. niruri*	Diuretic, litholytic, antilithogenic, antimicrobial, antioxidant, anti-inflammatory, antispasmodic	Significant reduction in size and number of stones; safe to use	Nisa and Astana (2019)
*Crateva nurvala*, *Musa x paradisiaca*, *Achyranthes aspera*,* Hordeum vulgare*	Indian traditional medicine formulation; likely litholytic and expulsive actions	Significant decrease in pain (VAS); significant decrease in surface area and density of calculi; significantly higher expulsion of calculi; better expulsion and size/density reduction than placebo	Patankar et al. (2020)
*Tribulus terrestris*, *Urtica dioica*, *Adiantum capillus-veneris*, *Stigma maydis*,* Cucumis melo*	Diuretic, antispasmodic, antioxidant	Significant increase in 24-h urine volume; significant reduction in stone size; significantly higher complete stone expulsion rate; potential treatment	Samandarian et al. (2023)
*Centaurii herba*, *Levistici radix*, *Rosmarini folium*	Moderate diuretic and antispasmodic action; improves urodynamics, contributes to speedy elimination of calculi/fragments post-ESWL	Speedy elimination of calculi fragments post-ESWL; higher percentage of patients with complete elimination of fragments at 2 weeks post-ESWL	Vitkovskyy (2021)

The first table focused on the phytotherapeutic agents assessed across the included trials. For each herbal remedy, the table presents its botanical name, proposed or documented mechanisms of action, and a concise overview of its quantitative clinical effects as reported in the trials. Mechanistic pathways were derived from both traditional ethnopharmacological knowledge and evidence reported within the included studies and were later grouped under themes such as urinary chemistry modulation, stone dissolution, and expulsion-promoting effects (e.g. diuretic, antispasmodic, or anti-inflammatory actions). Each plant was then linked to its respective clinical outcomes, with numerical results such as mean differences, confidence intervals, *P*-values, and rate of response highlighted where available. This summary table served as a bridge between traditional botanical knowledge and clinical data, illustrating how plant-based therapies are currently being validated in evidence-based medicine.

The second SoF table provided a structured overview of the included randomized clinical trials. For each trial, key methodological and clinical data were summarized, including the study aim, conclusion, country of origin, total duration of the study and follow-up, and total sample size, with a breakdown of patients per intervention and control arm. The identification of each arm, including the intervention name, dosage, route, and formulation, was also presented to support reproducibility and allow comparisons across studies. Besides, clinical outcomes were reported in a quantitative format, including effect sizes, confidence intervals, and *P*-values for primary and secondary endpoints such as stone size, number, and expulsion rates. This detailed compilation of results enabled a side-by-side comparison of trial efficacy and supported refined synthesis in both narrative and statistical forms.

Collectively, these SoF tables offered a comprehensive yet accessible overview of the review’s evidence base. The integration of phytotherapeutic mechanisms with clinical trial outcomes helped to provide how and to what extent herbal interventions may contribute to kidney stone management. These tables also suggest a valuable foundation for identifying knowledge gaps and informing future research on the efficacy, standardization, and safety of plant-based therapies for urolithiasis.

### Quantitative synthesis and meta-analysis

Meta-analyses were performed on two primary outcomes: stone size (measured in millimeters) and stone number (defined as the number of stones per patient) in order to objectively evaluate the clinical efficacy of herbal therapies in the treatment of kidney stones. These outcomes were chosen based on their availability in extractable numerical form appropriate for statistical pooling and their consistent clinical relevance across trials. These meta-analyses were conducted using RStudio, applying a standard approach to calculate mean differences (MD) and standardized mean differences (SMD) where appropriate, along with corresponding 95% confidence intervals (CIs). Data points were extracted from the included trials based on final endpoint values or change from baseline, and only those studies reporting means, standard deviations, and sample sizes for both intervention and comparator groups were considered eligible for inclusion. A fixed-effects model was applied as the default analytical method; however, in cases where substantial heterogeneity was detected, a random-effects model was believed to be more appropriate to account for variability across studies.

Out of the 14 trials included in the systematic review, only some reported outcomes in a statistically complete and standardized manner. Since the outcomes measured across the studies varied in their reporting methodologies, it was complicated to synthesize quantitative results and pooled conclusions from the meta-analysis of the 14 studies. Therefore, only seven trials were eligible for inclusion in the meta-analysis of stone size reduction, and four trials were included in the meta-analysis of stone number decrease. The remaining studies were excluded from the quantitative synthesis for various reasons. These included reporting outcomes in non-numerical terms (e.g. only percentages or descriptive results), using inconsistent measurement units without sufficient detail to allow for conversion, missing standard deviations or sample sizes, or presenting only qualitative outcomes such as “stone expulsion observed” without precise metrics. In some cases, data were reported as medians with interquartile ranges or ranges that could not be reliably transformed into mean and standard deviation without introducing bias.

**Fig. 4:**
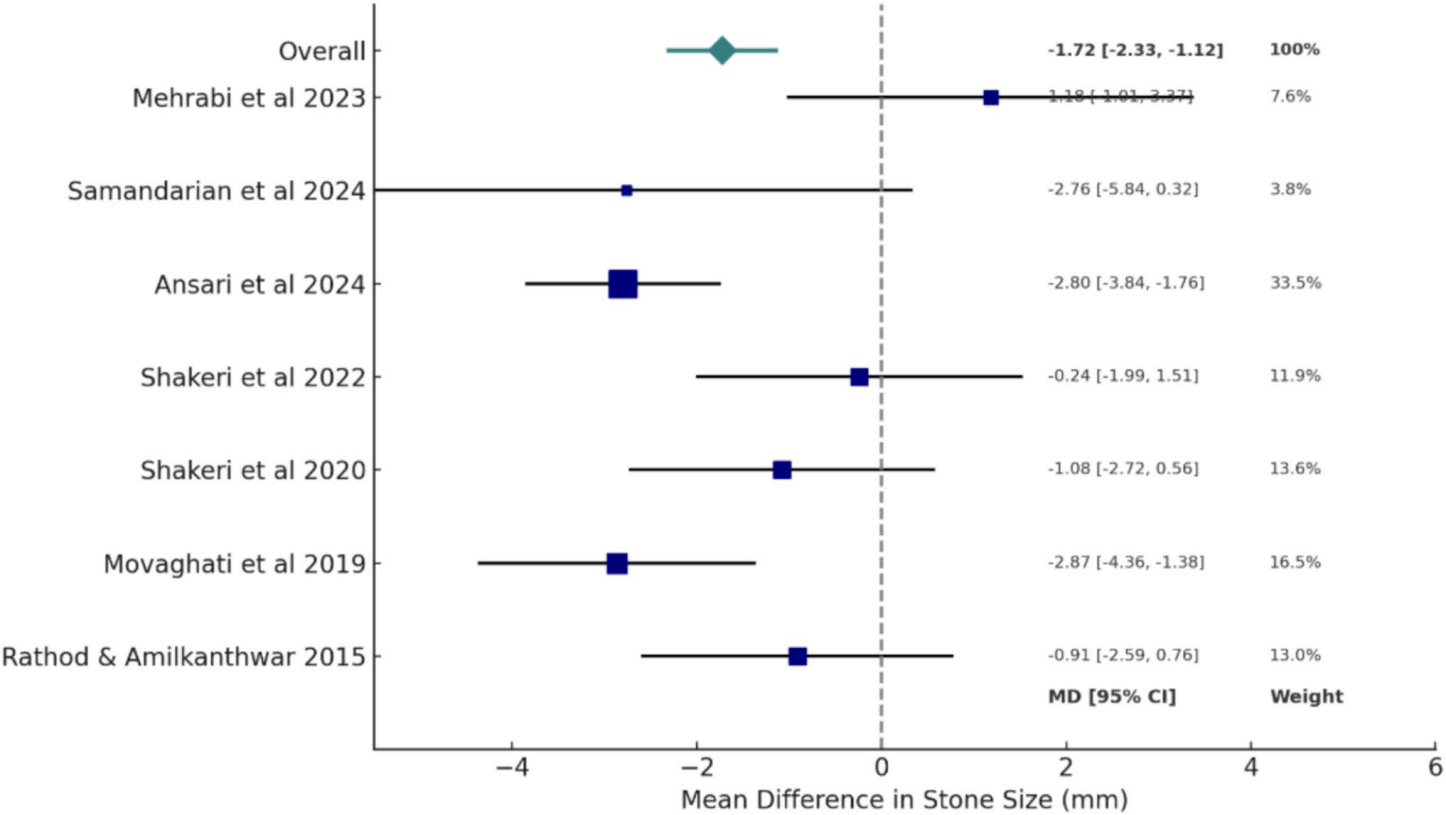
Forest plot: herbal interventions vs control on stone size

**Fig. 5:**
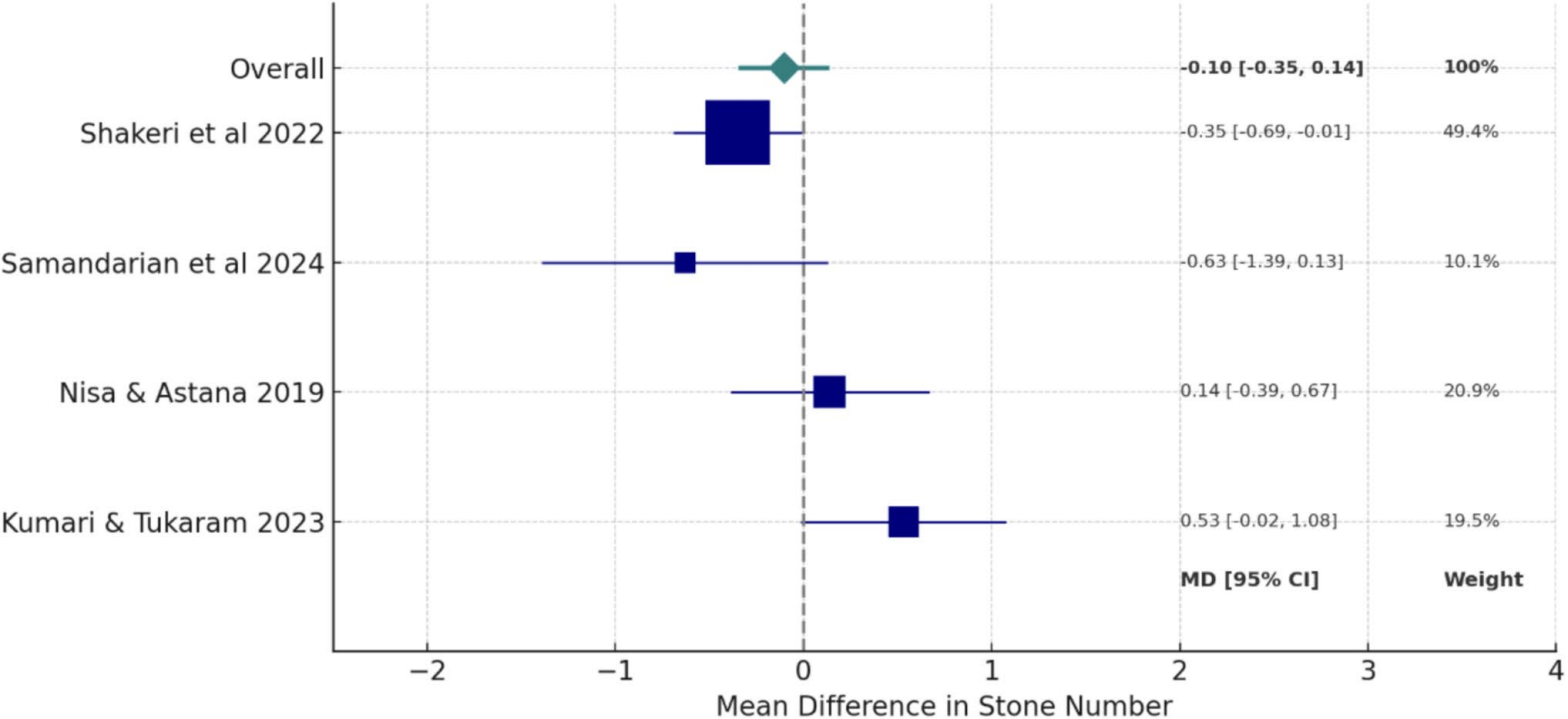
Forest plot: herbal interventions vs control on stone number

Furthermore, both the chi-squared test (χ^2^) and the *I*^2^ statistic were calculated to assess statistical heterogeneity across studies. The *I*^2^ value provided a quantitative estimate of inconsistency between study results, with thresholds of 25%, 50%, and 75% representing low, moderate, and high heterogeneity, respectively. When *I*^2^ exceeded 50%, sensitivity analyses were undertaken to explore potential sources of variation. These analyses involved excluding studies with outlier effect sizes or substantially different methodological characteristics, as well as reweighting studies based on their sample size and variance to evaluate the robustness of the overall effect estimates. Additionally, forest plots (Figs. 4 and 5) were generated for both outcomes, visually summarizing the individual and pooled effects of phytotherapeutic interventions on stone size and number. In each forest plot, the square markers represented the weighted mean of individual study effects, and horizontal lines denoted confidence intervals. The pooled effect was illustrated as a diamond at the top of the plot.

Overall, the meta-analytic component of the review provided an evidence-based quantitative summary of the clinical benefits of phytotherapy in kidney stone management, complementing the qualitative synthesis. This process also highlighted the need for more standardized outcome reporting in future phytotherapy trials to facilitate better data comparability and synthesis.

### Natural remedies in kidney stones management

Phytotherapy offers diverse approaches to managing kidney stones, with various plants and herbal formulations exhibiting unique therapeutic actions. The clinical trials included in this review investigate several plant-based interventions, each targeting different aspects of stone formation, growth, or expulsion through their distinctive pharmacological properties, as shown in Table 4.

#### Portulaca oleracea

Purslane, or *Portulaca oleracea*, is a medicinal plant that has been used for centuries and is known to have anti-inflammatory and antioxidant qualities. Although *P. oleracea*’s precise mode of action is still unclear, a number of possible aspects of its effects have been proposed based on its composition and observed characteristics. This plant’s potential antioxidant qualities are attributed to its abundance of antioxidant vitamins, including glutathione, β-carotene, α-tocopherol, and ascorbic acid. It has been suggested that these antioxidant effects could influence the formation of renal stones and calcium oxalate crystals. Additionally, *P. oleracea* shows promise in reducing inflammation. Additionally, this plant’s traditional uses and other research associate it with qualities like muscle-relaxant effects. Although the precise ways in which *P. oleracea* affects specific urinary parameters are not yet fully understood, studies have shown that administration of *P. oleracea* powder resulted in a significant increase in urine citrate and a decrease in urine calcium levels, which is consistent with its potential preventive effect on kidney stone formation. To date, no clinically important adverse events associated with *P. oleracea* use have been reported in studies. More research is recommended to further clarify its mechanism of action, safety, and efficacy (Alirezaei et al. 2023).

#### Alhagi maurorum

*Alhagi maurorum* is a plant traditionally used for various medical purposes, including the removal of kidney and bladder stones in regions such as Iran, Palestine, and Jordan (Aryaeefar et al. 2022). While the specific mechanism of action for its effects on kidney stones requires further clarification (Alirezaei et al. 2023), several potential properties have been proposed (Mehrabi et al. 2023). One suggested mechanism is a diuretic effect, which leads to increased urine volume and enhanced excretion of sodium and potassium (Aryaeefar et al. 2022). This action may lead to flushing the urinary tract (Rathod and Amilkanthwar 2015). The plant is also thought to have spasmolytic or relaxation properties (Mehrabi et al. 2023), which could contribute to the removal of stones by relaxing the smooth muscles of the urinary tract and possibly dilating the ureter (Aryaeefar et al. 2022). This relaxation effect may also help relieve the severe pain associated with stone passage. Key compounds suggested to participate in these effects include alkaloids, which may induce relaxation by releasing catecholamines, and antioxidant flavonoids, which might help expand the ureter (Aryaeefar et al. 2022). In addition, the antioxidant components within the plant may alleviate pain caused by stones and potentially protect kidney tissue. Other anticipated mechanisms include an inhibitory effect on urease and a reduction in acidity and crystallinity of urine by its methanol extract. Besides, studies using aqueous extracts suggest a potential for *A. maurorum* to help prevent calcium oxalate crystal formation by reducing oxalate and increasing urinary citrate, despite some findings indicating it has a limited effect on dissolving existing crystals. The plant’s hydroalcoholic extract is also associated with spasmolytic effects on smooth muscle and may have a dilating action on the urinary tract, supporting stone passage (Mehrabi et al. 2023).

#### Nigella sativa

Black seed, or *Nigella sativa*, is a medicinal plant that is used to treat kidney stones in Traditional Persian Medicine (Ardakani Movaghati et al. 2019). While its particular mechanisms require further clarification through dedicated studies (Ansari et al. 2024), several potential modes of action have been suggested based on its composition and preclinical research. According to its traditional uses, *N. sativa* has diuretic qualities and may also have antiurinary retention effects. This herb has been shown in preclinical research to have positive effects on kidney stones and related renal damage. Numerous extracts can considerably lower the quantity and size of kidney calcium oxalate deposits, according to animal research examining the effects of *N. sativa.* Additionally, the ethanolic extract showed promise in reducing calcium oxalate levels in urine. The alcoholic extract might be especially useful in preventing the kidneys and urinary tract from accumulating calcium oxalate crystals (Ardakani Movaghati et al. 2019).

Melanin, butanol components, and thymoquinone, a significant constituent, are important bioactive substances thought to contribute to these effects. It has been proposed that thymoquinone is effective for both preventing and fragmenting kidney stones. Its antimicrobial effects may also be relevant for preventing stones of bacterial origin, such as struvite stones (Ardakani Movaghati et al. 2019). Components like thymoquinone, butanol components, and melanin are also linked to the plant's antioxidant and anti-inflammatory properties, which are thought to be relevant to its effects on kidney stones. Studies using aqueous extracts of *N. sativa* have shown an ability to inhibit the growth of calcium oxalate monohydrate particles in vitro. Additionally, results from some studies evaluating urinary and tissue parameters have indicated a significant reduction in calcium crystal formation and oxalate excretion (Shakeri et al. 2021).

#### Peganum harmala

*Peganum harmala*, commonly known as wild rue or harmel, is a medicinal herb used in traditional medicine for various purposes, including treating renal diseases, disinfecting urinary tracts, and possibly having analgesic effects. The seeds of *P. harmala* are specifically recognized for their useful components for managing various renal diseases. Numerous bioactive substances are known to be present in that plant, including flavonoids and alkaloids like quinazoline and beta-carboline compounds. Moreover, preclinical studies and traditional medicine support the idea that *P. harmala* has multiple mechanisms of action related to kidney stones. One of these is the ability to crush stone. Its traditional use also suggests a diuretic effect, which increases urine volume and potentially assists in flushing stones and their passage. This plant is also characterized by analgesic properties, which could help improve the pain caused by the presence or movement of stones; this effect may be linked to the alkaloid extract and flavonoids present in the seeds. Furthermore, traditional uses indicate an ability to disinfect the urinary tract, which could be relevant in preventing stones associated with infection. While clinical studies have investigated the effects of *P. harmala*, observing outcomes such as a decrease in urinary stone size and number and a reduction in pain, these observations are consistent with the proposed mechanisms and properties of the plant. Thus, further research is recommended to fully clarify the specific mechanisms by which *P. harmala* exerts its effects on urinary stones (Shakeri et al. 2020).

Other than these aforementioned single-herb interventions, several polyherbal formulations recognized in traditional medicine systems have been clinically evaluated for their efficacy in kidney stone management, utilizing the potential synergistic effects of multiple plant ingredients.

#### Mofatet powder

The Mofatet powder is a polyherbal formulation from Traditional Persian Medicine (TPM), traditionally used for kidney and bladder stones. This formulation is composed of equal parts of seeds from *Cucumis sativus*, *Cucurbita pepo*, *Apium graveolens*, *Foeniculum vulgare*, and *Pimpinella anisum*, along with the fruit of *Tribulus terrestris*. While the specific mechanism of action of the Mofatet powder formulation needs further study to be fully understood, potential mechanisms have been linked to its constituents. For instance, the methanolic extract of *Cucurbita pepo* seeds has been suggested to potentially inhibit all stages of calcium oxalate stone formation, including nucleation, accumulation, and growth. Additionally, reports claim that *Tribulus terrestris* can dissolve kidney stones, which has been attributed to a protein isolated from it that can convert oxalate. Besides, *Apium graveolens* has diuretic effects that have been considered a factor in the dissolution of calcium stones. That is why observing a reduction in stone size in a clinical study using this formulation may indicate the solubilizing activity of the plant extract. Furthermore, multiple ingredients of Mofatet powder are stated to have renal protective effects, such as *Cucurbita pepo*, *Cucumis sativus*, *Foeniculum vulgare*, and *Pimpinella anisum*. Thus, this preparation was evaluated in the Ansari et al. clinical trial and implied to be effective in reducing calcium kidney stone size without potential nephro- or hepato-toxicity. Further detailed in vitro and in vivo studies are necessary to discover the specific mechanisms of action of this polyherbal formulation (Ansari et al. 2024).

#### Antiurolithic herbal formula (AHF)

The AHF is a polyherbal mixture composed of dried simplisia from the leaves of *Strobilanthes crispus*, *Orthosiphon stamineus*, and *Sonchus arvensis*, along with the roots of *Imperata cylindrica*, *Curcuma longa* (turmeric), *Curcuma xanthorrhiza* (temulawak), and *Phyllanthus niruri* (meniran). This formulation is hypothesized to exert its effects on urinary stones through the synergistic potential activity of its different ingredients. So, the proposed mechanisms of action for the AHF blend are multifaceted, including diuretic, antimicrobial, antioxidant, anti-inflammatory, antispasmodic, litholytic (stone dissolving), and antilithogenic (stone preventing) activities. The antilithogenic effect may involve increasing urolithiasis inhibitors by elevating urinary citrate excretion and decreasing urinary calcium and oxalate, leading to a reduction in the risk of stone formation. Each of the aforementioned unique mechanisms has been attributed to individual components or properties of that blended formula. For instance, *Orthosiphon stamineus* is known for its diuretic effect, and *Strobilanthes crispus* leaves are thought to have a strong diuretic activity related to potassium, helping to expel calcium oxalate stones. This diuretic action increases urine volume, which potentially assists in flushing the urinary tract. And for stone dissolution and prevention, *Sonchus arvensis* leaves are suggested to be effective in dissolving kidney stones, with water extracts potentially being more effective than alcohol extracts. *Orthosiphon stamineus* can also prevent the formation of calcium crystals. So, these ingredients may act at various stages of stone formation, including nucleation, crystal growth, aggregation, and retention (Nisa and Astana 2019).

Furthermore, the antimicrobial and antioxidant properties of the AHF are categorized as antilithogenic effects that help prevent stone formation. In particular, *Curcuma longa* and *Curcuma xanthorrhiza* are considered for their antioxidant and antimicrobial abilities. In general, the medicinal plants containing antioxidant compounds can prevent calcium oxalate precipitation and reduce oxalate excretion. In addition, the formula is proposed to possess anti-inflammatory activity, as regulation of inflammation is considered a potential strategy in stone management, and antispasmodic activity, which could support the passage of stones. Nonetheless, herbs like *Curcuma xanthorrhiza* and *Imperata cylindrica* are also associated with hepatoprotective effects, although this relates to the safety profile rather than the direct mechanism on stones. While clinical evaluation has been conducted, further detailed in vitro and in vivo studies are necessary to fully discover the specific mechanisms of action of this complex polyherbal formulation (Nisa and Astana 2019).

#### Subap Plus

The Subap Plus is an herbal preparation recognized in Indian traditional medicine and investigated for its anti-urolithiasis effects. This formulation is prepared from the extracts of *Crateva nurvala*, *Musa x paradisiaca*, *Achyranthes aspera*, and *Hordeum vulgare*. Besides, the anti-urolithiasis potential of these plants has been supported by in vitro and in vivo studies. They have shown that the mechanism of action of Subap Plus is suggested to involve a synergistic effect of its combined ingredients, since the plant extracts used in the formulation are thought to act at various stages of stone formation, including nucleation, crystal growth, aggregation, and retention. In particular, the mentioned constituents may contribute to lowering the rate of stone-forming salts/minerals and increasing urinary citrate excretion. *Hordeum vulgare*, for instance, is noted to have such actions, in addition to its anti-inflammatory properties. Beyond affecting stone formation, the mixture is also thought to possess litholytic activity, helping to break down or dissolve stones, which could further explain observed reductions in size and density of calculi. An expulsive action is also proposed, which may lead to a higher rate of stone passage (Patankar et al. 2020).

Furthermore, animal studies held on this preparation have shown significant restorative activity on injured renal cells caused by crystal deposition, which is referred to as a “reno-protective function.” This is considered critically important because cellular injury may play a role in promoting and progressing kidney stones. That is why regulating inflammation is also considered a potential therapeutic pathway in stone management. Besides, the formulation has shown favourable effects on urinary chemistry parameters relevant to stone formation, including effects on hypercalciuria, hyperoxaluria, hypocitraturia, hypomagnesuria, and the supersaturation index, based on unpublished data from another study by the same authors. Thus, the overall anticipated mechanisms encompass preventing stone formation at multiple stages, facilitating stone breakdown and passage, protecting kidney cells from injury, and potentially modulating urinary composition. The formulation is also suggested to have analgesic activity, which helps reduce pain caused by renal stones. However, further detailed studies are deemed necessary to fully reveal the specific mechanisms of action of this herbal formulation (Patankar et al. 2020).

#### A five-herb oral solution

This oral solution containing extracts of *Tribulus terrestris*, *Urtica dioica*, *Adiantum capillus-veneris*, *Stigma maydis* (corn silk), and *Cucumis melo* was clinically evaluated for the treatment of urolithiasis. Its potential therapeutic effects are suggested to be due to the combination of pharmacological actions exerted by its ingredients, including diuretic, anti-inflammatory, antispasmodic, antioxidant, pH-altering, and urinary ion-concentrating effects. Each of these specific mechanisms is attributed to its components; for example, the diuretic effect is mediated by herbs like *Tribulus terrestris*, *Urtica dioica*, and *Stigma maydis*. Animal studies emphasize the diuretic activity of *T. terrestris and U. dioica*, while *S. maydis* is recognized not only for its diuretic activity but also for its kaliuretic properties, which help in potassium excretion. These effects suggest the capacity to increase urine volume, which can help in flushing the urinary tract. This solution is also thought to exert antilithogenic effects, acting at various stages of stone formation as mentioned before: nucleation, crystal growth, aggregation, and retention. Moreover, *Urtica dioica* is believed to inhibit calcium and oxalate accumulation, preventing crystal growth, as proven by studies in rats showing reductions in urinary calcium, oxalate, and crystal deposition. *Adiantum capillus-veneris* is also reported to inhibit urinary crystallization and decrease crystal size and number, as emphasized by rat studies observing decreased blood calcium and urea, and reduced crystal counts in urine. Similarly, *Tribulus terrestris* has also shown activity against experimentally induced urolithiasis in rats, reducing crystal size and number (Samandarian et al. 2023).

In addition, this formulation may influence urinary composition by affecting ion concentrations and pH, addressing known risk factors like hypercalciuria, hyperoxaluria, hyperuricosuria, and hypocitraturia. Also, antioxidant properties are essential for counteracting crystal-induced kidney damage and preventing stone formation. This is because oxidative stress and inflammation play significant roles in urolithiasis pathogenesis, as exposure to calcium oxalate crystals can lead to reactive oxygen species production, tubular damage, and inflammation, promoting crystal aggregation. Fortunately, all five plants in this solution are noted for being rich in phenolic compounds and flavonoids, and their confirmed antioxidant properties could contribute to the formula’s effectiveness. Another potential mechanism is the antispasmodic effect on the smooth muscles of the urinary tract, which could facilitate stone elimination. These antispasmodic effects have been previously demonstrated for *Adiantum capillus-veneris* and *Tribulus terrestris*. Thus, the formula is suggested to have multifaceted activities recognized strategies in stone management (Samandarian et al. 2023).

#### BNO 1040

An herbal preparation containing a standardized BNO 1040 extract, derived from levage root (*Levistici radix*), rosemary leaves (*Rosmarini folium*), and aerial parts of centaury (*Centaurii herba*), has been studied for its effects, particularly following ESWL. This fixed combination of herbal ingredients provides a complex effect on the kidneys and urinary tract. Its proposed mechanisms of action include a moderate diuretic effect and antispasmodic action, which are believed to improve urodynamics and consequently contribute to a speedy elimination of calculi. Aside from these actions influencing stone passage, the preparation is also thought to act on non-specific factors involved in stone formation. This may involve reducing the saturation of urine with stone-forming salts/minerals and increasing the concentration of magnesium ions in the urine, which is considered a natural crystallization inhibitor. The herbal constituents can also affect indirect factors contributing to calculus formation, such as inflammation and disturbance of urodynamics. Additionally, they are thought to inhibit the development of pathogenic flora in the urine, which helps prevent the exacerbation of inflammatory processes during the elimination of stone fragments. This wide range of actions, including influencing urine composition, reducing spasms, increasing urine volume, and potentially reducing inflammation and infection, contributes to the preparation’s overall effect on the urinary tract calculi (Vitkovskyy 2021).

Combining the discussed suggested mechanisms of action for different herbal formulations offers an interesting insight into their several possibilities in treating urolithiasis. Besides, several common therapeutic modalities show up across the formulations addressed. Among these are diuretic effects, meant to increase urine volume so aiding in urinary tract flushing. In addition, frequently mentioned are antispasmodic actions, which represent a possibility to relax the urinary tract’s smooth muscles so enabling the passage of stones or fragments. Many preparations are also believed to affect urinary composition by reducing the concentration of stone-forming constituents like calcium and oxalate and raising the levels of crystallization inhibitors like citrate and possibly magnesium. Apart from these, other essential activities are suggested. Antioxidant properties are emphasized for their role in counteracting the oxidative stress and damage caused by crystal formation in the renal tubules, thus preventing stone pathogenesis. Some formulations propose anti-inflammatory effects, highlighting inflammation as a factor in renal calculi management. Litholytic effects, denoting the ability to break down or dissolve stones, are also recommended as explanations for observed reductions in stone size and density. Additional mechanisms, particularly related to specific preparations like reno-protective function through helping to restore renal cells damaged by crystal deposition, are noted as important. There is also a suggestion that inhibiting pathogenic flora in the urine could play a role in preventing complications during stone fragment elimination. The following table (Table 4) provides a comprehensive summary of the mechanistic approaches for the addressed single and poly herbal formulations.

*VAS* Visual Analogue Scale, *ESWL* Extracorporeal Shockwave Lithotripsy

### Clinical trials on herbal therapy for kidney stones

After having explored the plausible mechanisms by which different phytotherapeutic agents might exert therapeutic effects in urolithiasis, including actions like diuresis that increase urine flow, relaxation of the urinary tract via antispasmodic properties, alteration of the urinary chemical environment to inhibit crystal formation, reduction of oxidative stress, and possibly breakdown of the existing stones, the next crucial step in determining their clinical utility is to evaluate whether these mentioned hypothesized actions translate into real tangible patient benefits. The included clinical trials in this review provide the vital framework to transition from theoretical mechanism to witnessed outcome, since they examine a range of parameters intended to measure the clinical impact of the interventions. Such measures of stone expulsion and passage, changes in stone size and number, changes in urinary composition, and symptom relief, such as pain reduction, are often included in these evaluations, which often focus on important indicators that correspond with the assumed effects. Building on the mechanistic basis previously discussed, we can start exploring the degree to which these herbal preparations exhibit efficacy in treating urolithiasis by investigating the statistical and clinical data obtained from these trials.

#### Modulation of urine composition (preventive effect)

Moving beyond the possible underlying mechanisms of action, we now analyze how these herbal interventions result in quantifiable outcomes in the clinical setting, first concentrating on their impact on the urinary environment. Changing the physicochemical characteristics of urine to reduce the chance of stone formation is indeed an important approach in the management and prevention of urolithiasis. This can be accomplished by changing the concentration of chemicals that either encourage or prevent crystal nucleation, growth, and aggregation. Still, a major area of research is examining this modification of urine composition as a preventative measure. Within the included clinical trials, only two offer insights into this category of activity: the study by Alirezaei et al., which evaluated the effects of *Portulaca oleracea* extract on key 24-h urine indices in patients with renal stones (Alirezaei et al. 2023), and the trial conducted by Gao et al., focusing on the *Huayu Jianpi Fangshi* decoction’s impact on urinary calculus inhibitors and other parameters in patients who had been cured of urolithiasis (Gao et al. 2021). These RCTs provide practical evidence regarding the ability of specific herbal interventions to directly influence the chemical elements within the urine that are critical to stone pathogenesis.

Factors such as urinary levels of inhibitors like citrate and promoters like calcium are essential in the process of stone formation. One clinical trial that investigates this approach is the study by Alirezaei et al., which was a single-centre, randomized, double-blind, placebo-controlled clinical trial conducted at Shahid Modarres Educational Hospital in Tehran, Iran. It included 54 participants (28 in the *P. oleracea* group and 26 in the placebo group), who had a documented history of renal stones and normal kidney function and had completed the trial. Those participants were randomly assigned to receive either capsules containing 2 g of *Portulaca oleracea* powder mixed with brown sugar or identical placebo capsules made of starch and brown sugar, taken once daily for 8 weeks on an empty stomach. Outcome measurements, including 24-h urine levels of citrate, calcium, uric acid, creatinine, and sodium, were eventually assessed at baseline and again after the 8-week study period (Alirezaei et al. 2023).

The study results demonstrated statistically significant changes in the key urine chemistry parameters. After 8 weeks of intervention, the mean urine citrate level in the *P. oleracea* group (674.82 ± 94.56 mg/24 h) was significantly higher compared to the placebo group (579.19 ± 85.06 mg/24 h, *P* < 0.01). On the contrary, the mean urine calcium level in the *P. oleracea* group (176.32 ± 27.40 mg/24 h) was significantly lower than that in the control group (194.26 ± 25.17 mg/24 h, *P* = 0.016 or *P* = 0.02). Although urine creatinine levels were not significantly affected, these findings indicate a favourable shift in the urinary environment towards reduced stone formation risk. The trial also states that baseline differences in these parameters were not significant between the groups, allowing the observed changes to be attributed only to the intervention. Based on these significant modulations of urine citrate and calcium, which are known risk factors for nephrolithiasis, the study concludes that *Portulaca oleracea* may offer a preventive effect on kidney stone formation (Alirezaei et al. 2023).

Following on from the Alirezaei study, the second RCT within this category that contributes to our understanding of modulating urine composition for stone prevention is the primary research conducted by Gao et al. Their study examined the effects of *Huayu Jianpi Fangshi* decoction (HJFD), a traditional Chinese medicine (TCM) herbal formula recognized for its principles, particularly in preventing urolithiasis by influencing factors such as stone inhibitors and addressing underlying patient constitutions, including spleen malfunction and ureteral obstruction. It also aimed to confirm the decoction’s mechanism achieved by regulating the concentration and expression of calculus inhibitors and explore its TCM preventive mechanism. It was designed as a randomized, double-blinded, placebo-controlled clinical trial. It was conducted at the China Academy of Chinese Medical Sciences Guanganmen Hospital in Beijing, China, recruiting a population consisting of patients aged 18–60 years who had been previously diagnosed with and cured of upper urinary tract urolithiasis and also met specific TCM diagnostic criteria related to qi stagnation, blood stasis, spleen deficiency, and dampness obstruction. Initially, 60 eligible patients were randomized, with 30 assigned to the treatment group and 30 to the control group. However, 57 patients completed the trial (29 in the treatment group, 28 in the control group), receiving either the HJFD decoction or an identical placebo decoction, both taken orally two times per day. The detailed composition of the HJFD decoction included herbs such as *Rhizoma curcumae*, *Atractylodes macrocephala*, and *Lysimachia christinae*. All patients also received the same dietary education regarding urolithiasis prevention, with a total duration for the intervention and study follow-up of 4 weeks. Ultimately, outcome indicators were measured at baseline (time of enrolment) and again after 4 weeks of medication (Gao et al. 2021).

As addressed, the Gao et al. trial assessed various parameters, focusing on the main urinary calculus inhibitors and other urine composition factors. Statistically significant results concerning urine modulation included the following: After 4 weeks, the mean 24-h urinary citric acid level in the treatment group (297.48 ± 57.91 mg/L) was highly statistically significantly higher compared to the control group (244.75 ± 59.62 mg/L), with a difference between the groups being highly significant (*P* < 0.01). The study also observed a significant decrease in the urinary concentration of Tamm-Horsfall protein (THP) in the treatment group after 4 weeks compared to before treatment (*P* < 0.01), and the difference in urinary THP concentration between the groups after treatment was statistically significant (*P* < 0.05), with the treatment group having a lower level (6.37 ± 6.10 mg/L) than the control (10.83 ± 7.73 mg/L). Similarly, the concentration of inter-α-trypsin inhibitor heavy chain 3 (ITIH3) significantly decreased within the treatment group after 4 weeks (*P* < 0.01), and the difference between the treatment group (6.14 ± 4.46 mg/L) and the control group (9.51 ± 6.32 mg/L) after treatment was referred to as statistically significant (*P* < 0.05). There was also a significant increase in urinary pH after treatment in the HJFD group (*P* < 0.01), and this rise was statistically significantly higher than in the control group (*P* < 0.01). Additionally, a significant decrease in urinary calcium level was observed within the treatment group after treatment (*P* < 0.01), and the difference between the treatment group (6.90 ± 0.54) and the control group (7.57 ± 0.64) after treatment was highly statistically significant (*P* < 0.01). Urinary magnesium, oxalic acid, and uric acid levels did not show any significant differences between groups after the intervention. So, it was concluded that the HJFD can increase the excretion of urinary citric acid and downregulate the abnormal expressions of urinary THP and ITIH3, which are considered micromolecular and macromolecular calculus inhibitors, respectively. These findings, along with improvements in TCM symptoms and constitution, suggest that the decoction has a positive significance in the prevention of urolithiasis (Gao et al. 2021).

Reflecting upon the outcomes from both the Alirezaei et al. and Gao et al. trials, we can see preliminary evidence suggesting that phytotherapeutic interventions may be promising for preventing kidney stone formation by beneficially altering urine composition. The study of Alirezaei et al. on *Portulaca oleracea* indicated that its administration led to statistically significant increases in urine citrate and decreases in urine calcium, parameters critical for inhibiting crystal formation. Similarly, the Gao et al. trial investigating the HJFD decoction reported significant increases in urinary citric acid and pH, alongside significant reductions in urinary calcium, THP, and ITIH3 concentrations. These collective observations from both RCTs aim towards a favourable shift in the urinary environment, potentially making it less favourable to the nucleation, growth, and aggregation of stone-forming crystals. However, it is crucial to consider the inherent limitations acknowledged by the authors themselves. Both were single-centre studies with relatively small sample sizes (54 in Alirezaei and 57 in Gao) and short intervention durations (8 weeks and 4 weeks), which hinders the generalizability and the ability to definitively assess long-term stone recurrence rates. Both also highlighted the need for further evaluation of safety and efficacy and a more in-depth mechanistic investigation. Therefore, while these initial findings are encouraging, they serve primarily as a foundation, emphasizing the need for more robust, multi-centre trials with larger cohorts and extended follow-up periods to confirm these effects, evaluate long-term clinical benefits, and fully understand the mechanisms by which these interventions influence stone prevention.

#### Stone dissolution (size reduction)

After meticulously discussing interventions targeted at preventing newly formed stones by altering urinary chemistry, we would address the potential for natural or traditional medicine to directly affect existing kidney stones through dissolution or size reduction. This represents a unique therapeutic modality, acting on actively diminishing the physical burden of pre-existing calculi rather than just modifying the environment in which stones form. Since calcium-based kidney stones constitute the majority of stone types and there is currently no proven medical treatment with a well-established and efficient method for dissolving them, it is an exclusively crucial area of research. Within the selected studies, three RCTs specifically explore this activity: the study by Ansari et al. (2024), which evaluated the efficacy of Mofatet powder, a polyherbal formulation from Traditional Persian Medicine, in patients with calcium kidney stones; the trial conducted by Ardakani Movaghati et al. (2019), which assessed the renal stone-dissolving efficacy of *Nigella sativa* (black seed); and the trial by Nisa and Astana (2019), which compared the AHF against a commercial lithotripsic agent, using changes in stone size and number as key outcome measures. The objective of each of these investigations was to find out whether the interventions in question could result in a measurable reduction in the size or total elimination of pre-existing stones.

Focusing on the Ansari et al. trial as one of the studies exploring stone dissolution, it investigated the safety and efficacy of Mofatet powder, a polyherbal formulation derived from TPM, specifically for the treatment of calcium kidney stones. This formulation contains the seeds/fruits of six previously stated plants: *Cucumis sativus*, *Cucurbita pepo*, *Apium graveolens*, *Foeniculum vulgare*, *Pimpinella anisum*, and *Tribulus terrestris*. The study was conducted as a randomized, double-blinded, placebo-controlled clinical trial at clinics associated with Shiraz University of Medical Sciences in Shiraz, Iran, including patients aged over 18 diagnosed with renal stones 5 mm or larger located in the lower pole of the kidney, confirmed to be calcium stones. Out of 70 surveyed, 51 patients were randomized (26 to the drug group, 25 to the placebo group) using a computer-generated block randomization method with sealed envelopes to ensure allocation concealment, but 49 patients completed the study (26 in the drug group and 23 in the placebo group). Participants in the treatment group received 500 mg capsules of the Mofatet powder extract twice daily, administered orally, while the control group received identical placebo capsules. The Mofatet capsules were prepared by boiling the powdered herbal mixture to obtain an aqueous extract, which was then concentrated, freeze-dried, and encapsulated to standardize the dosage. The intervention and follow-up period lasted for 5 weeks, allowing for measurement of outcome parameters, including stone size and various biochemical markers in blood and urine, which were evaluated at two time points: before the intervention (baseline) and after 5 weeks of treatment. The declared baseline characteristics, including average stone size (8.15 ± 1.64 mm in the drug group and 7.95 ± 2.12 mm in the placebo group), were comparable between the groups (Ansari et al. 2024).

The key finding of the Ansari et al. trial was the significant impact on existing stone size, confirmed by the imaging results after 5 weeks, which showed a highly statistically significant decrease in stone size in the drug group compared to their baseline (*P* < 0.001), while no significant change was observed in the placebo group (*P* = 0.39). Additionally, the mean reduction in stone size in the drug group was 3 mm, representing a 60.73% decrease. The difference in stone size change between the drug and placebo groups was also highly statistically significant (*P* < 0.001). Besides, the study reported the disappearance of stones in two patients in the drug group, with none in the placebo group. Regarding urine composition, the drug group showed a significant decrease in 24-h urine calcium concentration after 5 weeks compared to baseline (*P* = 0.02), and this decrease was also statistically significant compared to the placebo group after treatment (*P* = 0.02). Concurrently, urinary magnesium significantly increased in the drug group after 5 weeks compared to baseline and also compared to the placebo group after treatment (*P* < 0.001). Also, urine specific gravity in the drug group after treatment was significantly lower than in the placebo group (*P* = 0.015). Comparatively, no significant changes were observed in 24-h urine volume within or between groups. Likewise, evaluation of renal and hepatic function tests (BUN, creatinine, AST, ALT) showed no significant changes in either group before or after treatment, or between groups after treatment, suggesting no observable nephro/hepatotoxicity. Importantly, no clinically significant adverse reactions were observed during the trial. The study eventually concluded that Mofatet powder was effective in reducing the size of calcium kidney stones and was safe, supporting its potential as a treatment for nephrolithiasis. These observed changes in urinary calcium, magnesium, and specific gravity were highlighted as potential mechanisms related to reducing supersaturation and inhibiting crystal formation (Ansari et al. 2024).

For the second trial, conducted by Ardakani Movaghati et al., it targeted the use of black seed (*Nigella sativa* L.) to investigate its potential for dissolving existing kidney stones. It was held in Mashhad, Iran, specifically at the urology clinic of Emam Reza Hospital, and designed as a randomized, triple-blind, placebo-controlled clinical trial. Sixty patients with kidney stones larger than 5 mm were initially enrolled for this research and randomized into two parallel groups: 30 patients assigned to the black seed group and 30 to the placebo group using simple block randomization. However, 27 patients in the black seed group and 26 in the placebo group were included in the final analysis for some key outcomes. Those allocated in the active group received 500 mg capsules of ground black seed powder twice daily (total 1000 mg/day), taken orally with honey syrup, while the control group received identical placebo starch capsules, also with honey syrup. Both groups were also given dietary advice for kidney stone patients for the period of the intervention and follow-up that lasted for 10 weeks, with outcome measures assessed at baseline and after the 10-week treatment period (Ardakani Movaghati et al. 2019).

The primary outcome measured in the Movaghati RCT was the change in renal stone size using sonography. At baseline, the average stone sizes were comparable between the groups (6.20 ± 1.65 mm in the black seed group and 6.41 ± 1.51 mm in the placebo group, *P* = 0.296), proving that a non-statistically significant discrepancy existed between the two enrolled groups. After 10 weeks, the results showed a highly statistically significant decrease in stone size within the black seed group (*P* = 0.000), whereas the placebo group showed no significant change (*P* = 0.098). Furthermore, the difference in the mean stone size between the black seed and placebo groups after the study was highly significant (*P* = 0.000), indicating a much greater reduction in the black seed group. Beyond just size reduction, the study reported that 44.4% of patients in the black seed group completely excreted their stones, compared to only 15.3% in the placebo group, a statistically significant difference (*P* = 0.035). The probability of stone excretion was approximately three times higher with black seed (relative risk = 2.88, 95% CI = 1.06–7.81). Additionally, 51.8% of patients in the black seed group experienced a reduction in stone size, compared to just 11.5% in the placebo group. On the contrary, stone size increased in 15.3% of the placebo group patients, while this was not reported for the black seed group. Secondary outcomes included changes in urine pH and serum calcium, whereas the urine pH significantly decreased in the black seed group after intervention compared to baseline (*P* = 0.046) and was significantly lower compared to the placebo group after treatment (*P* = 0.002). Conversely, serum calcium significantly increased within the black seed group after 10 weeks (*P* = 0.001), but this change was not significantly different compared to the placebo group after treatment (*P* = 0.732). Considering safety, renal and hepatic function tests showed no significant adverse changes, and while one patient in the black seed group was excluded due to hydronephrosis and increased blood pressure, the authors cite previous studies demonstrating the safety of black seed at this dose and its tendency not to increase blood pressure, suggesting this individual case might not be directly attributable to the intervention. Overall, the study concluded that *Nigella sativa* L. had significant positive effects on the disappearance or reduction of kidney stone size compared to placebo (Ardakani Movaghati et al. 2019).

And for the third study, investigating the dissolution power and reducing renal calculi size, led by Nisa and Astana, evaluated an AHF against a commercial polyherbal lithotripsic (CPL) agent. It was located in Indonesia at the medicinal plant and traditional medicine research and development centre, and designed as a purposive randomized open-label clinical study with end-blinded observation. Out of 307 screened patients, 200 adults with urolithiasis and stones smaller than 20 mm were enrolled and randomized by computer into two groups, and eventually 97 patients in the AHF group and 94 in the CPL group were analyzed. The AHF consisted of dried simplisia from seven previously mentioned plants: *Sonchus arvensis*, *Orthosiphon stamineus*, *Strobilanthes crispus*, *Imperata cylindrica*, *Curcuma xanthorrhiza*, *Curcuma domestica*, and *Phyllanthus niruri*. Subjects in the AHF group were instructed to boil 10 g of *S. arvensis*, 6 g of *O. stamineus*, 4 g of *S. crispus*, 5 g of *I. cylindrica*, 5 g of *C. xanthorrhiza*, 4 g of *C. domestica*, and 3 g of *P. niruri* in 1 L of water for 15 min and drink the filtered water twice daily. While the control CPL group received capsules containing extracts from some similar plants (*O. stamineus*, *S. crispa*, *S. arvensis*, *P. niruri*) plus *Plantago major*, taking one capsule four times daily. The intervention and observation period lasted for 8 weeks, whereas stone size and number, alongside safety parameters (liver and renal function), were assessed at baseline and the end of the study (8th week), with some parameters also checked at the 4th week. Besides, baseline demographic and stone characteristics were similar between the groups (Nisa and Astana 2019).

The efficacy outcomes in the Nisa and Astana study primarily concentrated on changes in stone size and number, showing results that the AHF group experienced a significant size reduction in both single and multiple stones after 8 weeks compared to baseline (*P* < 0.05 for single stones, *P* = 0.007 for multiple stones). The CPL group also showed a significant size reduction for single stones (*P* = 0.006), but not for multiple stones (*P* = 0.071). Crucially, the difference in the mean size reduction of single stones was significantly greater in the AHF group (mean reduction 4.53 ± 6.81 mm) compared to the CPL group (mean reduction 0.49 ± 9.57 mm) (*P* = 0.009). For multiple stones, the size reduction difference between groups was not statistically significant (*P* = 0.798). And regarding the number of stones, the AHF group presented a significant decrease in the number of both single and multiple stones after 8 weeks compared to baseline (*P* < 0.05 for both). In contrast, the CPL group exhibited no significant change in the number of stones. The study noted a significant difference between the two groups at 8th week concerning the number of stones and described that the total difference in stone dissolution or reduction was greater in the AHF group, suggesting AHF could dissolve stones about twice as effectively as CPL (ratio 1.9:1). Both groups also showed a significant reduction in pain measured by Visual Analog Scale (VAS) from baseline, though there was no significant difference in VAS reduction between the groups at the end of the study (Nisa and Astana 2019).

Moreover, safety was assessed by monitoring liver function (SGOT, SGPT) and renal function (BUN, creatinine, potassium), indicating that the AHF was safe, with biochemical parameters for liver and renal function remaining within normal limits throughout the 8-week intervention in both groups. So, there were no statistically significant differences in these parameters between the AHF and CPL groups at any time point, nor significant changes from baseline within each group, except for a significant decrease in SGPT levels in the AHF group at the 4th week compared to baseline. The study eventually concluded that the Antiurolithic Herbal Formula was both safe and effective for treating urolithiasis, and that the observed efficacy is attributed by the authors to the synergistic litholytic, diuretic, and potentially antioxidant and anti-inflammatory activities of the herbal ingredients (Nisa and Astana 2019).

These three trials collectively offer promising initial evidence that certain traditional and herbal therapies may possess the potential for the non-invasive management of existing kidney stones. The polyherbal Mofatet powder demonstrated a notable reduction in calcium stone size, exceeding 60% over just 5 weeks, even leading to complete disappearance in two patients. Separately, research on black seed (*Nigella sativa* L.) indicated its effectiveness in increasing the rate of complete stone excretion (44.4% vs 15.3% placebo) and achieving greater stone size reduction compared to placebo over 10 weeks, with a stone excretion likelihood approximately three times higher. Furthermore, the AHF showed a significant advantage over the CPL comparator in reducing single stone size and the overall number of stones, suggesting AHF might dissolve stones about twice as effectively as CPL. Importantly, safety assessments across these trials generally revealed no significant negative effects on kidney or liver function from the tested interventions. However, these findings are preliminary and limited by factors such as the relatively short duration of the interventions, ranging from 5 to 10 weeks, which restricts the evaluation of long-term outcomes and potential recurrence. Some studies also noted the need for larger patient populations to confirm the results obtained. Additionally, the reliance on imaging methods like ultrasonography and KUB, while practical, was regarded as potentially less accurate and objective than more advanced techniques like CT scans for precise stone size measurement. Therefore, future research is recommended to aim for larger clinical trials with extended follow-up periods to confirm efficacy and assess recurrence, employ more objective imaging modalities whenever reasonable, and potentially explore concentrated extracts or variations in dosing or formulation to enhance effectiveness, particularly for larger stones.

#### Stone expulsion (diuretic and antispasmodic)

We now shift our focus from treatments that mainly aim to dissolve or reduce the size of stones to a complementary approach to managing urolithiasis: facilitating existing stones to pass and be expelled. This method frequently utilizes characteristics like diuretic effects to boost urine flow and physically remove stones or fragments and antispasmodic effects to relax the urinary tract’s smooth muscles, which may help the stone’s elimination. The results of nine clinical trials will be discussed to investigate the evidence for herbal and traditional interventions functioning through these mechanisms. These include studies by Aryaeefar et al., Kumari and Tukaram, Mehrabi et al., Patankar et al., Rathod and Amilkanthwar, Samandarian et al., Shakeri et al. (2020), Shakeri et al. (2021), and Vitkovskyy. Each of these trials investigates various formulations and their potential to aid in the expulsion of urinary tract stones.

The first study examining stone expulsion, by Aryaeefar et al., investigated the potential of a unique traditional remedy, *Alhagi maurorum*, in Neyshabur, Iran, within a clinical hospital setting, and focused on patients experiencing renal colic due to ureteral stones. The researchers implemented a randomized, single-blind clinical trial design involving 110 patients whose data were ultimately analyzed after randomization into groups. These participants, consisting of 75 men and 35 women, were divided into either a control group (*n* = 55) or an intervention group (*n* = 55). The control group received routine treatment, which consisted of a once daily 0.4 mg tamsulosin capsule and a 100 mg diclofenac suppository taken every 12 h, while patients in the intervention group received this same routine treatment in addition to the herbal intervention (Aryaeefar et al. 2022).

This *Alhagi maurorum* distillate was administered orally at a dose of 150 cc per day, divided into three doses (50 mL each). This specific preparation, purchased from a licensed company, had its concentration confirmed at 1.5 mg/100 mL. Both the intervention and control regimens were followed for 4 weeks, with patients being followed up weekly. The primary outcome measured was stone excretion and the time it took for the stone to pass. Statistical analysis using Cox regression revealed that the time of stone excretion was significantly shorter in the intervention group compared to the control group. Precisely, the analysis adjusted for variables like stone size, location, and history of disease showed a hazard ratio (HR) of 5.51 (95% CI, 2.81, 10.8) with a highly significant *P*-value (< 0.001), indicating that patients receiving *Alhagi maurorum* were much more prone to expel their stones sooner than those on routine treatment alone. Thus, the administration of the distillate was associated with approximately a 4-day reduction in stone removal time. Supportively, the study reported that none of the patients experienced any complications or side effects with the use of the *Alhagi maurorum*, and tolerance in terms of taste was described as desirable. Besides, baseline characteristics, including mean age (intervention, 40.12 ± 11.6 years; control, 42.83 ± 8.1 years; *P* = 0.16) and mean stone size (intervention, 5.24 mm; control, 5.06 mm; *P* = 0.35), were found to be comparable between the two groups (Aryaeefar et al. 2022).

The same plant was studied by Mehrabi et al., to be compared with a conventional medication commonly used for kidney and ureteral stones. This trial was a randomized, prospective study carried out in Yasuj, Iran, within a clinical hospital setting, specifically at a urology clinic, and initially assessed 80 patients over 18 years of age with kidney and upper ureteral stones ranging in size from 4 to 10 mm. Using a block random allocation method, patients were divided into two groups: one receiving hydrochlorothiazide (control) and the other receiving the herbal intervention, *Alhagi maurorum*. Only 76 patients completed the trial and were analyzed, with 40 in the *Alhagi maurorum* group and 36 in the hydrochlorothiazide group, due to four patients being lost to follow-up in the control group. Baseline characteristics, including mean age, sex, mean number of stones, mean size of stones, serum urea, and serum creatinine levels, were reported as similar between the two groups before the intervention, emphasizing the elimination of any confounding factors. For instance, before treatment, the mean age was 45.1 ± 12.77 years in the *Alhagi* group and 46.17 ± 14.42 years in the hydrochlorothiazide group (*P* = 0.73). Also, the mean stone size before treatment was mentioned as 13.3 ± 9.163 mm in the *Alhagi* group and 10.79 ± 6.82 mm in the hydrochlorothiazide group (*P* = 0.178) (Mehrabi et al. 2023).

The intervention group received a hydroalcoholic aerial extract of *Alhagi maurorum*, which was prepared from the aerial parts of the plant using 70% ethanol alcohol. Patients in this group received 1 g per day of the extract, administered orally in two 500 mg capsules taken separately every 12 h with water. While the control group received hydrochlorothiazide tablets (50 mg), one tablet every night. Both groups also received oral diclofenac sodium for pain relief and were instructed to drink ample water and exercise. The duration of the intervention was 2 weeks, with patients being visited again after this period to record and analyze follow-up tests and ultrasound results. Outcome measurements were taken before the intervention and after the 2-week treatment period (Mehrabi et al. 2023).

The study’s key findings focused on comparing the outcomes between the two groups after the 2-week intervention, whereas after treatment, the mean number of stones was 1.33 ± 0.88 in the *Alhagi* group and 0.94 ± 0.79 in the hydrochlorothiazide group (*P* = 0.052), demonstrating no statistically significant difference between groups. Similarly, the mean stone size after treatment was 6.93 ± 4.15 mm in the *Alhagi* group and 5.75 ± 5.71 mm in the hydrochlorothiazide group (*P* = 0.314), again with no statistically significant difference observed between the groups. While the size and number of stones decreased after intervention within both groups, the comparison between the groups did not indicate a statistically significant difference. Regarding treatment efficacy (defined as stone removal or residual stones < 4 mm), complete efficacy was achieved in 8 (20%) of the *Alhagi* group and 14 (38.9%) of the hydrochlorothiazide group, with a *P*-value of 0.305, representing no statistically significant difference in overall efficacy between the two treatments. The study also found that taking either treatment had no significant effect on renal function tests (serum urea and creatinine levels), with *P*-values for urea being 0.351 and for creatinine being 0.588 after treatment when comparing between groups. Remarkably, no specific complications or side effects were observed in either group. The authors concluded that *Alhagi maurorum* is as effective as hydrochlorothiazide for the treatment of kidney and ureteral stones within the 4–10 mm range, with no significant complications. They also recommend that more detailed studies with larger sample sizes and longer durations are needed to confirm these effects and understand the efficacy on different stone sizes and materials (Mehrabi et al. 2023).

Another trial, accomplished by Kumari and Tukaram, studied an Ayurvedic approach to urolithiasis. This open-labelled, placebo-controlled clinical trial was conducted in Jamnagar, Gujarat, India, at the Outpatient and Inpatient departments of Shalya Tantra of IPGT and RA hospital, aiming to evaluate the effect of two traditional formulations on Mutrashmari (urolithiasis). A total of 43 patients were initially randomized using a computer-generated method into two groups, and after accounting for loss to follow-up, 39 patients were analyzed: 20 patients in the trial group (Group A) and 19 patients in the placebo control group (Group B). The study included patients aged 18–70 years with renal and ureteric stones up to 10 mm, while excluding those with chronic renal failure, gross hydronephrosis, or stones larger than 10 mm, among other conditions (Kumari and Tukaram 2022).

The intervention in Group A consisted of two preparations: *Palasha Kshara* and *Ashmarihara Kwatha*. *Palasha Kshara* is an alkali prepared from the ash of the whole *Butea monosperma* plant, while *Ashmarihara Kwatha* is a decoction made from a combination of 13 different plant parts, including roots, fruits, seeds, stem bark, and leaves, from plants such as *Bergenia ligulata*, *Carica papaya*, *Asparagus racemosus*, *Tribulus terrestris*, *Crataeva nurvala*, *Cucumis sativus*, *Desmostachya bipinnata*, *Saccharum spontaneum*, *Tectona grandis*, *Oryza sativa*, *Boerhavia diffusa*, *Tinospora cordifolia*, *Achyranthes aspera*, *Nardostachys jatamansi*, and *Hyoscyamus niger.* These preparations were procured from the Pharmacy of Gujarat Ayurved University and authenticated. Furthermore, patients in Group A received a 500 mg capsule of *Palasha Kshara* orally three times a day after meals and 40 mL of *Ashmarihara Kwatha* twice daily after meals. On the other hand, the control group (Group B) received a 500 mg placebo capsule (granulated wheat) orally after meals. Both groups were advised to take 3–4 L of water over 24 h and were provided with a recommended diet chart and followed up for the treatment duration that was 60 days (2 months). Patients were also assessed at 2-week intervals up to 2 months, with a follow-up 1 month after treatment completion to check for recurrence and adverse effects (Kumari and Tukaram 2022).

Regarding outcomes, the study evaluated both subjective complaints and objective parameters. In Group A, treatment showed statistically significant results in relieving pain (Vedana), increased frequency of burning micturition (Sadaha Mutrata) (*P* < 0.0001 for pain, *P* < 0.001 for increased frequency of micturition), though not for haematuria (*P* = 0.125). Compared to placebo (Group B), Group A demonstrated a significant difference in the relief of pain (*P* < 0.00001) and increased frequency of micturition (*P* = 0.0064), but not for haematuria (*P* = 0.61) or burning micturition (*P* = 0.96) when comparing the effect between groups. Objectively, Group A showed statistically significant improvements in stone size, position, number of stones, and the presence of hydronephrosis/hydroureter (HN/HU). Concerning stone size, the mean difference was 0.950 with a *P* < 0.0001. In Group B, only the position of the stone demonstrated a significant change, while stone size, number, and HN/HU did not. Comparing the groups, there was a significant difference in results for all objective parameters measured, including stone size (*P* = 0.0005), position (*P* = 0.0345), number (*P* = 0.0333), and HN/HU (*P* = 0.0106). Specifically, in Group A, 29.73% of kidney stones and 75% of ureteric stones were expelled, and 51.35% of kidney stones and 25% of ureteric stones decreased in size. In contrast, in the placebo group, only 20% of kidney stones were expelled, 37.14% decreased in size, while a substantial 37.14% increased in size, and all ureteric stones (100%) increased in size. The researchers concluded that *Palasha Kshara* and *Ashmarihara Kwatha* were effective in both the symptomatic management and the expulsion of small-sized stones (< 10 mm) (Kumari and Tukaram 2022).

Similarly, Rathod and Amilkanthwar evaluated the effectiveness of an Ayurvedic preparation, *Kadalikshar*, in managing Mutrashmari through conducting an RCT. This trial took place at a Government Ayurved College in Nanded, Maharashtra, India, with a total of 60 patients diagnosed with Urolithiasis, confirmed by ultrasound sonography (USG), who were selected and equally divided into two groups of 30 each. Those participants of either sex, aged 16 to 60 years, with stones up to 20 mm in size were included, while patients with renal function impairment, pregnant or lactating women, or those with certain systemic conditions like hypertension (HTN), diabetes mellitus (DM), ischemic heart disease (IHD), congestive heart failure (CCF), tuberculosis (TB), or Asthma were excluded. The intervention group received *Kadalikshar* in a 500 mg capsule form, taken orally twice a day before meals. It was prepared from the dried, burned ash of Kadali (*Musa Sapientum*) stems, which were then filtered and evaporated to obtain a powder. On the other hand, the control group received *Gokshuradi Yog*, a traditional formulation consisting of *Gokharu*, *Erandmul*, *Wagharimul*, *Bruhatimul*, and *Talimkhana churna* mixed with honey and curd, also taken orally twice a day before meals. The treatment was administered for 28 days in both groups, with follow-up assessments conducted on the 0th, 7th, 14th, 21st, and 28th days, with a final assessment on the 45th day (Rathod and Amilkanthwar 2015).

The study assessed both subjective symptoms (pain, burning micturition, haematuria) and objective criteria (size, site, and number of stones). Regarding stone size, measured by USG, the mean size at the beginning of the study was 6.423 ± 2.799 mm in the Trial group and 6.394 ± 2.570 mm in the Control group. At the end of the 28-day treatment period, the mean size in the Trial group was 2.189 ± 3.448 mm, while in the Control group it was 3.102 ± 3.158 mm, displaying a reduction in size from baseline (before treatment–after treatment), which was of a mean of 4.309 ± 2.655 mm in the Trial group and 3.311 ± 2.444 mm in the Control group. Furthermore, statistical analysis (unpaired *t*-test) found a significant difference in the mean reduction of stone size between the groups (*P* < 0.05), indicating the Trial drug was more effective. Evaluation of stone number also revealed that in the Trial group, 33.96% of stones dissolved, 22.64% were expelled, and 41.33% were significantly reduced in size. While in the Control group, 11% dissolved, 9% were expelled, and 60.37% were significantly reduced. Overall, *Kadalikshar* was found to be more effective in promoting the dissolution and expulsion of stones than *Gokshuradi Yog*. Concerning subjective symptoms, while chi-squared tests at individual follow-ups were often not statistically significant, indicating both drugs provided some relief, the overall results showed higher percentages of patients were relieved in the Trial group compared to the Control group for pain (73.33% vs 53.33%), burning micturition (79.16% vs 65%), and haematuria (76.46% vs 66.66%). And based on the clinical assessment, 53.33% of patients in the Trial group were completely relieved, compared to 33.33% in the Control group. Conclusively, the effectiveness of Kadalikshar is attributed to its traditional Ayurvedic properties and its alkaline nature (pH 12.2), which may help dissolve stones and act as a diuretic to facilitate expulsion, which is very effective in managing urolithiasis, revealing superiority over Gokshuradi Yog (Rathod and Amilkanthwar 2015).

Patankar et al. had also conducted a clinical trial in a tertiary care hospital in Pune, India, between September 2010 and December 2013. This prospective, randomized, double-blind, placebo-controlled, parallel-group study aimed to investigate the effects of an herbal formulation on renal calculi, recruiting initially 120 patients who were screened, but 84 were randomized. However, 65 patients completed the trial: 34 assigned to receive the herbal treatment and 31 to receive the placebo. Eligible participants were individuals with asymptomatic renal calculi measuring between 4 and 9 mm, confirmed by a non-contrast CT scan. Yet, exclusions encompassed patients requiring immediate surgical intervention or those with significantly abnormal baseline liver or kidney function tests. The herbal intervention, Subap Plus capsules, was prepared from extracts of *Crateva nurvala*, *Musa x paradisiaca*, *Achyranthes aspera*, and *Hordeum vulgare*, herbs traditionally associated with properties like anti-urolithiatic, diuretic, antispasmodic, and anti-inflammatory activity. Patients in the treatment group received one 500 mg capsule of this formulation orally twice daily after meals for 6 months. Comparatively, the control group received identically appearing capsules containing lactose, following the same dosage schedule. Besides, the follow-up occurred monthly to monitor for pain and stone expulsion, with a final CT scan performed at the end of the 6-month treatment period or upon reported expulsion (Patankar et al. 2020).

The study measured various outcome parameters at baseline and the end of the 6-month intervention period, focusing on stone characteristics and symptoms. One of which was stone expulsion, occurring in seven patients (20.6%) in the herbal treatment group compared to only one patient (3.2%) in the placebo group; the difference was found to be statistically significant (*P* = 0.03). Regarding stone size, assessed by surface area, the mean change revealed a significant reduction in the treatment group from a baseline of 27.6 ± 15 to 21.7 ± 13.2 sq. mm post-treatment (*P* = 0.0243 within the group). In contrast, the placebo group displayed a slight, non-significant increase in mean surface area from 31.5 ± 16.5 to 32 ± 16.1 sq. mm (*P* = 0.28 within the group). The difference in the magnitude of change between the two groups was highly statistically significant (*P* < 0.005). Stone density, measured in Hounsfield units (HU), also presented a beneficial trend in favour of the treatment group, decreasing from a mean of 834.3 ± 357.3 to 740.9 ± 338 HU (*P* = 0.062 within the group). The placebo group, however, experienced a significant increase in mean stone density from 901.9 ± 314.7 to 1059.6 ± 405.9 HU (*P* = 0.008 within the group). Also, the difference in density change between the groups was statistically significant (*P* < 0.001). And for Pain, assessed by the Visual Analog Scale (VAS), significantly decreased in the herbal group from 6.9 ± 1.6 at baseline to 1.8 ± 0.9 post-treatment (*P* < 0.0001). Comparatively, the placebo group showed only a minor, non-significant change in mean VAS score from 7.2 ± 1.5 to 6.8 ± 1.5 (*P* < 0.161), leading to a difference in pain reduction between the groups that was highly significant (*P* < 0.0001). These findings suggest that the herbal formulation effectively promotes stone expulsion and favourably alters stone characteristics (reducing size and density) while also significantly relieving pain, hence presenting a promising outcome for patients with small, asymptomatic renal calculi. Favourably, the study concluded that the herbal formulation demonstrated anti-urolithiatic and analgesic activity without major adverse events (Patankar et al. 2020).

A randomized, single-blind, placebo-controlled clinical trial was conducted in Isfahan, Iran, from October 2019 to October 2020, by Samandarian et al. to assess the efficacy of an oral solution containing five herbal extracts in treating urolithiasis. Initially, 109 patients were evaluated, with 89 meeting the inclusion criteria, and 62 consenting to participate, but 54 patients eventually completed the study, divided equally into a drug group (*n* = 27) and a placebo group (*n* = 27). Those participants eligible for the study were aged 18 years or older, had symptomatic or asymptomatic kidney stones up to 10 mm in size confirmed by ultrasonography, and were referred from Nephrology or Urology clinics. Conversely, exclusions involved conditions like malignancy with bone metastasis, hyperthyroidism, hyperparathyroidism, psychosis, impaired renal function (serum creatinine above 1.4 mg/dL), structural urinary tract disorders or active UTI, uncontrolled gout or hyperuricemia, recent use of other traditional remedies for urolithiasis, pregnancy, or lactation. The herbal intervention was an oral drop solution prepared as mentioned before from the hydroalcoholic extracts of *Tribulus terrestris* leaf, *Urtica dioica* root, *Adiantum capillus‑veneris* leaf, *Stigma maydis* (silks), and *Cucumis melo* seeds, standardized to 1.2 mg phenolic content per mL. Patients in the drug group received 60 drops of this solution orally three times daily for 4 weeks, alongside standard treatment, while the placebo group received an identically appearing oral solution prepared with the same solvents and given at the same dose and duration. Besides, patients in both groups were advised to maintain a high fluid intake (at least 2 L/day urinary volume) and follow nutritional recommendations, including salt and protein restrictions (Samandarian et al. 2023).

The study effects measured included stone number and size reduction by ultrasonography, and complete stone expulsion. At the end of the 4-week intervention, the outcomes exhibited a significant positive impact in the group receiving the herbal solution in terms of the parameters previously stated. Regarding stone parameters, the mean stone size decreased significantly in the drug group compared to the placebo group (*P* = 0.049). Specifically, the percent reduction in stone size showed a mean change of − 37.60 ± 74.77 in the drug group compared to − 7.55 ± 69.47 in the placebo group. While the mean number of stones decreased significantly within the drug group from 1.70 ± 1.03 to 1.11 ± 1.25 (*P* = 0.009), this change was not statistically significant when compared directly to the placebo group at the end of the study (*P* = 0.093). Crucially, the number of cases with complete stone expulsion was significantly higher in the drug group (12 cases, 44.44%) compared to the placebo group (4 cases, 14.81%) (*P* = 0.017). Although 24-h urine volume increased significantly within the drug group (*P* = 0.003 within group), the change was not statistically significant compared to the placebo group (*P* = 0.236 between groups). Other measured urinary parameters like calcium, sodium, citrate, oxalate, urea, creatinine, and uric acid did not display significant changes compared to baseline or significant differences between the groups. Finally, the study advocates that potential mechanisms for the herbal solution’s effectiveness include a diuretic effect mediated by components like *T. terrestris*, *U. dioica*, and *S. maydis*, supported by the increase in urine volume in the drug group. Furthermore, the significantly higher rate of stone expulsion in the drug group could be attributed, in part, to potential antispasmodic effects on the urinary tract smooth muscles from herbs like *A. capillus-veneris* and *T. terrestris*. The conclusion drawn is that oral consumption of this herbal solution effectively leads to stone size reduction and stone expulsion in patients with urolithiasis, proposing its potential as a supplemental treatment (Samandarian et al. 2023).

Another RCT testing oral *Peganum harmala* seed, which is traditionally known for its analgesic and diuretic properties, contributing to its effect on pain relief and potentially aiding stone passage, versus tamsulosin. It was held at a urology clinic in Iran between June 2018 and May 2019, although the intervention was for 2 weeks. This study enrolled 80 patients aged over 18 with kidney and ureteral stones sized between 4 and 10 mm who were candidates for medical therapy without indications for immediate intervention or severe pain, and excluded patients with conditions like cardiovascular/pulmonary disease, coagulopathy, uncontrolled hypertension, pregnancy, allergy, or contraindications to pain medication. Those patients were randomly allocated to one of two groups, with 40 patients initially in each arm. The intervention group received oral *Peganum harmala* seed prepared as a capsule at a dose of 50 mg/kg/day once daily after a meal for 2 weeks, while the control group received an oral capsule of tamsulosin 0.4 mg per night for the same 2-week period. Both groups were advised to have high fluid intake (10–12 glasses/day), exercise, and receive diclofenac sodium for pain control if needed. Patients were also revisited after 2 weeks, and urinary tract sonography was performed to assess changes in stone size and number and residual stones. Besides, pain severity was measured using a VAS. Findings indicated that mean stone size decreased in both groups after treatment, with mean sizes after treatment being 4.07 ± 3.66 mm in the *P. harmala* group and 5.15 ± 3.63 mm in the *tamsulosin* group. However, there were no significant differences between the two groups regarding mean stone size after treatment (*P* = 0.21), or in the change in size from baseline (*P* = 0.314 between groups before treatment, 0.21 after treatment). Also, the confidence intervals for the difference in mean stone size between groups confirmed the non-significance as they included zero, both before treatment (− 1.165 to 6.181) and after treatment (− 0.637 to 2.79). Similarly, no significant differences were observed between groups regarding stone numbers (*P* = 0.052 based on mean number of stones) or overall treatment efficacy (complete/partial response combined was 77.5% for *P. harmala* and 77.8% for tamsulosin, P = 0.06). Importantly, while pain score decreased significantly in both groups, the reduction was more significant in the *P. harmala* group (*P* = 0.002). So, the trial concluded that both *P. harmala* seed and tamsulosin effectively decreased urinary stone size and numbers without significant side effects, but *P. harmala* was significantly more effective in reducing pain (Shakeri et al. 2020).

Likewise, Shakeri et al. (2021) accomplished another RCT testing another medicinal plant, oral *Nigella sativa* seeds, versus tamsulosin. It was conducted in the same settings, following the same inclusion/exclusion criteria, but from March 2018 to March 2019. Its included patients were also 40 patients initially in each arm; however, the results presented appear to include data from 39 patients per group, as one patient in each arm was excluded due to lack of follow-up. The groups received oral *Nigella sativa* seeds, prepared as capsules, at a dose of 1 g every 12 h (total 2 g/day) after each meal with water, as the intervention, while tamsulosin was used for the control, as previously stated in the other trial. They were also advised to use diclofenac sodium for pain control and increase fluid intake with exercise. At the end of the 2-week treatment period, patients were similarly evaluated with urinary tract sonography to assess stone size and number, and pain severity was measured using a VAS. The findings implied that there were no significant differences between the groups in the mean size of stones after treatment (*Nigella*, 4.97 ± 4.33 mm vs. tamsulosin, 5.21 ± 3.63 mm, *P* = 0.39), although stone size decreased within both groups. Equally, stone numbers also decreased in both groups with no significant difference between them after treatment (*P* = 0.52). However, the pain score significantly declined in both groups after intervention, with the reduction being more significant in the *Nigella sativa* group (*P* = 0.001). Most notably, the overall efficacy of treatment (defined as complete or partial response/expulsion) was significantly higher in the *Nigella* group (78.5% or 79.5%) compared to the tamsulosin group (61.6% or 61.5%) (*P* = 0.005). Thus, the study concluded that while both *Nigella sativa* seeds and tamsulosin reduced urinary stone size and numbers without a significant difference between them, *Nigella sativa* demonstrated greater effectiveness in both stone passage/efficacy and pain control, supporting its potential use as an alternative treatment for urinary stones (Shakeri et al. 2021).

Finally, Vitkovskyy examined the effect of stone expulsion when introducing a natural herb to patients, particularly after ESWL. This study was a randomized clinical trial conducted at a urology clinic in Ukraine, using an herbal preparation based on lovage, rosemary, and centaury. It involved 150 patients aged 18–65 years with calcium oxalate urolithiasis and single stones sized 0.8–1.3 cm in the kidneys or 0.5–0.9 cm in the ureters, who had undergone ESWL, while excluding those with certain conditions like severe renal impairment, large calculus size (> 1.3 cm), or other stone compositions. Those patients were randomly divided into two equal groups, with 75 patients in the main group and 75 in the control group. The main group received standard recommendations plus an oral herbal preparation containing standardized BNO 1040 extract (based on lovage root, rosemary leaves, and aerial parts of centaury), whose dose was 2 tablets or 50 drops three times a day, unlike the control group, which received standard recommendations only. Standard recommendations for both groups included diet, adequate fluid intake (2–3 L/day), exercise therapy, and access to antispasmodic drugs (drotaverine hydrochloride) and painkillers (dexketoprofen) as needed, along with the ESWL procedure itself. The treatment with the herbal preparation lasted for 12 months, while follow-up evaluations occurred at several time points, including days 7, 14, 30, 45, and after a year. Consequently, the study duration, from the first visit to the 1-year follow-up, spanned approximately 12 months (Vitkovskyy 2021).

The findings demonstrated that the use of the BNO 1040 herbal preparation in conjunction with ESWL contributed to a more rapid and safe elimination of stone fragments. Specifically, complete elimination of fragments was observed significantly faster in the main group (mean time of 9.81 days) compared to the control group (12.23 days), with a statistically significant difference (*P* = 0.011). Additionally, by 14 days after ESWL, 94.7% of patients in the main group had achieved complete elimination of stone residuals, compared to only 76% in the control group, a difference that was also statistically significant (*P* = 0.042). While both groups experienced reduced pain and leukocyturia, the proportion of these complications was lower in the main group, although the difference in renal colic, in particular, did not reach statistical significance. Furthermore, recurrent stone formation within a year was observed in fewer patients in the main group (6.7%) compared to the control group (16%); however, this difference was not statistically significant due to the limited number of events. Besides, the trial highlights that the plant components of BNO 1040 are known to possess diuretic and antispasmodic actions, which can improve urodynamics and aid in faster calculus elimination. It also records that these constituents can potentially modulate the urinary environment by reducing saturation with stone-forming substances with increasing magnesium ion concentration and influencing inflammatory factors. Despite the study being conducted post-ESWL and primarily evaluating the impact on fragment expulsion and recurrence, the observed outcomes of faster elimination and potentially reduced recurrence are consistent with these acknowledged characteristics of the plants, which can affect both stone passage and modulate the urinary environment. In conclusion, the study revealed that the herbal preparation is effective and safe, accelerating fragment elimination after ESWL and potentially helping to prevent early recurrent stone formation, which still needs further investigation (Vitkovskyy 2021).

Given the range of clinical trials we have covered, which have examined everything from herbal extracts to traditional preparations for kidney stone expulsion and overall kidney stone management, it is evident that there is promising potential for them to enhance or complement conventional care, with several studies demonstrating beneficial effects on stone passage, size reduction, or symptom relief. For instance, trials investigating *Alhagi maurorum* distillate, the herbal blend (BNO 1040), the formulation called Subap Plus, *Kadalikshar*, and the mixture of *Tribulus terrestris*, *Urtica dioica*, *Adiantum capillus-veneris*, *Stigma maydis*, and *Cucumis melo* extracts, all reported improved stone expulsion rates or faster passage compared to control or placebo groups. Additionally, treatments such as *Nigella sativa*, *Kadalikshar*, Subap Plus, and other multi-herbal preparations have revealed positive effects in reducing stone size or density, as well as providing better pain control. However, a recurring theme across these studies, despite their varied approaches and specific findings, lies in their limitations, which naturally pave the way for vital future research. Some of the studies were constrained by relatively small sample sizes and limited study or follow-up durations, which impacted the statistical power to detect subtle but potentially important differences or long-term outcomes, such as recurrence prevention. Issues concerning study design, such as the absence of blinding in some cases or the reliance on self-reported data for certain variables, such as fluid intake or dietary habits when patients were not hospitalized, were also noted as potential influences on the results. Furthermore, some studies emphasized the need for a deeper understanding of the mechanism of action, advocating that future work should clarify the specific active constituents, their optimal dosages, and their effects on metabolic parameters or the urinary environment beyond simple diuretic or antispasmodic actions. Therefore, the collective recommendations are strongly directed towards conducting larger, well-controlled (ideally double-blinded) trials with longer follow-up periods, incorporating detailed metabolic examinations and potentially analyzing expelled stone compositions to build a more robust evidence base for integrating these promising therapies into standard urological practice.

In addition to the published clinical trials included and discussed, a registered study investigating the use of herbal interventions in kidney stone treatment was identified in our search, but had not yet published its final results at the time of this review. It is listed in ClinicalTrials.gov (ID: NCT03924596) and investigates the use of Frankincense or *Olibanum Boswellia* (Luban) in patients with kidney stones, using ultrasound to monitor changes in stone size and number as it categorizes calculi into radiopaque stones (calcium oxalate) and radiolucent stones (uric acid). This trial met the eligibility criteria for our review, being randomized, conducted in human subjects with urolithiasis, and focused on the impact of herbal preparation, but was eventually excluded due to the absence of publicly available results. Once published, their inclusion may strengthen or refine the current conclusions regarding the efficacy of phytotherapy in managing nephro/urolithiasis.

### Quality assessment of herbal intervention trials

Assessing the risk of bias within clinical trials is an essential step in understanding the reliability and validity of the findings. This approach helps us assess the likelihood that systematic errors, which can result from a number of study design and execution factors, including the randomization technique, blinding protocols, handling of missing data, and outcome measurement, will impact the study’s findings. So, we can interpret the reported efficacy and safety data of each trial more intelligently by carefully weighing these factors, which gives us a deeper and more comprehensive understanding of the quality of the evidence each trial provides. As shown in the following figure (Fig. 2) illustrating the distribution of risk of bias judgments across the five RoB 2.0 domains for the 14 included trials, it was evident that the quality assessment process is crucial as the chart highlights a predominance of high risk scores in domains related to missing outcome data and deviations from intended interventions, in contrast to the randomization process demonstrating comparatively higher methodological integrity. Since the missing outcome data and deviations from intended outcomes were the main reasons, several included studies were judged to have a high risk of bias, and the internal validity may be impacted by these methodological limitations. Therefore, we interpreted the results cautiously, taking into account these potential sources of bias during synthesis.

#### Low risk judgement

Upon reviewing the included trials, it seems that a number of them appear to have adopted robust methodologies aimed at reducing the risk of bias. For example, the Alirezaei et al. study investigating *Portulaca oleracea* (purslane) utilized a randomized, double-blind, placebo-controlled design (Alirezaei et al. 2023). The allocation was also achieved using computer-generated random numbers and a balanced block randomization method with sealed envelopes to conceal the assignment sequence. And importantly, the investigators, nurses, participants, laboratory staff, and supervisors were all stated to be blinded to the treatment assignment. The statistical analysis was also conducted using the intention-to-treat approach, which accounts for dropouts (Alirezaei et al. 2023). Similarly, the Movaghati et al. trial on *Nigella sativa* L. seeds used a randomized, double-arm, double-blind, placebo-controlled design. Simple block randomization was also used, and both participants and investigators were blinded, with similar-looking placebo capsules employed. Its primary outcome measure, change in renal stone size, was assessed by a blinded radiologist using sonography (Ardakani Movaghati et al. 2019). Additionally, the Patankar et al. trial evaluating the mixture herbal formulation (Subap Plus) for asymptomatic renal calculi followed a prospective, randomized, double-blind, placebo-controlled design with a parallel group structure. Block randomization was also implemented, and the principal investigator and enrolled patients were blinded. Besides, independent healthcare professionals handled randomization, enrolment, assignment, dispensing, and assessment, which further strengthens their robust methodology (Patankar et al. 2020). These aforementioned studies, by transparently reporting methods, like double-blinding and allocation concealment, provide a higher degree of confidence that the final observed outcomes are less likely to be due to systematic errors in the study process.

#### Some concern judgement

Conversely, some studies present potential risks of bias that require careful consideration. The Aryaeefar et al. study examining *Alhagi maurorum* distillate on ureteral stone expulsion was conducted as a randomized, single-blind clinical trial. The patients were not blinded to the intervention, but the data collector and analyzer were, which might have affected how subjectively they reported their symptoms or perceived the passage of stones. The authors themselves mentioned limitations, including the inability to evaluate food and drinking habits and the use of self-reported variables, which may not accurately reflect measures (Aryaeefar et al. 2022). Likewise, the trial by Gao et al. on *Huayu Jianpi Fangshi* decoction was a randomized, double-blind, placebo-controlled single-centre trial. Despite the blinding of TCM syndrome raters, the study was constrained by its single-centre design and small sample size. Additionally, the data analysis was conducted using the per-protocol approach (Gao et al. 2021), which can introduce selection bias if patients with specific outcomes disproportionately drop out from one group. “Purposive randomized open-label, end-blinded observation” was also the design employed in the Nisa and Astana study that compared a commercial polyherbal lithotripsic with an antiurolithic herbal formula. Although an independent radiologist evaluated the results and investigators were blinded to treatment allocation, the “open-label” design suggests that participants and possibly other people involved in daily care were not blinded, which could introduce performance or detection bias, particularly for subjective outcomes like pain (Nisa and Astana 2019). Similarly, the Samandarian et al. clinical trial evaluating a multi-herbal solution was randomized, single-blinded, and placebo-controlled, with a small sample size of 27 patients per group and a short duration of 4 weeks (Samandarian et al. 2023). Being single-blind can have an impact on results because it means that only one party, likely the assessor or investigator, but not both, was unaware of the treatment, whereas participants were most likely aware. The above factors imply that when interpreting the outcomes of these trials, a moderate degree of caution is necessary.

#### High risk judgement

A higher risk of bias exists in a number of the included RCTs because of incomplete reporting of key methodological details or particular design choices. For instance, the Kumara and Tukaram study comparing *Ashmarihara Kwatha* and *Palasha Kshara* to a placebo was an open-labelled placebo-controlled clinical trial, which poses a considerable risk of bias if “open-labelled” denotes that participants or researchers are not blinded, especially when it comes to subjective outcomes like pain relief or self-reported stone expulsion. Additionally, the comparison was against conservative management, including increased water intake (3–4 L), which is known to influence stone passage (Kumari and Tukaram 2022). In the same manner, the Shakeri et al. trial comparing *Alhagi maurorum* aerial extract to hydrochlorothiazide (Shakeri et al. 2020) and the other two trials comparing herbal interventions (*Peganum harmala* or *Nigella sativa*) to tamsulosin were defined as randomized clinical trials, but did not report their blinding status. Hence, when two potentially effective treatments are compared without blinding, performance or detection bias may arise. Furthermore, despite providing results tables, the Rathod and Amilkanthwar study comparing *Kadalikshar* and *Gokshuradi Yog* lacks details on the study design, randomization strategy, or blinding (Rathod and Amilkanthwar 2015). This makes a risk of bias assessment challenging from the provided data alone, but it also raises concerns. Finally, the trial evaluating the BNO 1040, a standardized herbal treatment, after ESWL by Vitkovskyy states follow-up practices and outcome measures but does not explicitly mention randomization or blinding processes (Vitkovskyy 2021), which are critical for minimizing bias in such a comparative study. Conclusively, the results of these studies should be interpreted very cautiously in the absence of clear reporting of these bias mitigation approaches, since systematic errors rather than the intervention alone may have contributed to the final observed effects.

In conclusion, the risk of bias assessment across these trials, as depicted in the previous figure (Fig. 3), reveals different degrees of confidence in their findings. This traffic-light grid shows variation in methodological quality, with domains like randomization process (D1) displaying relatively more studies with low risk or some concerns, while missing outcome data (D3) and deviations from intended interventions (D2) were most commonly rated as high risk. In general, studies that use rigorous strategies like allocation concealment and double-blinding provide more trustworthy results. On the other hand, research with limitations like open-label design, single-blinding, per-protocol analysis, or inadequate reporting of bias mitigation techniques necessitates a more cautious interpretation of their results. The highest risk of bias is found in trials that do not provide clear explanations of the randomization and blinding procedures, which might overestimate the perceived efficacy of the interventions. Mostly, small sample sizes, short-timed intervention or follow-up periods, single-centre designs, and reliance on less accurate imaging methods like sonography are common limitations observed in all these studies. Future research would strongly benefit from adhering to standard reporting guidelines to document randomization, allocation concealment, and blinding procedures. Additionally, the generalizability and clinical relevance of the results would be improved by using multi-centre trials with larger sample sizes and longer follow-up times. This long-term duration is essential to fully evaluate the risk profile of the investigated interventions, because many of the included studies were insufficient and inconsistent in reporting adverse effects, which may underrepresent potential safety concerns associated with herbal and nutraceutical therapies Any prospective studies in this field would also be more valid if objective outcome measures were used whenever feasible and proper statistical analysis (such as intention-to-treat) was used.

### Herbal medicine key clinical evidence

Herbal therapies represent a notable area of interest in the management of urolithiasis, especially given the high prevalence of kidney stones and the limitations or potential side effects associated with conventional treatments (Alirezaei et al. 2023). That is why traditional medicine systems have widely utilized various plants for treating urinary stones (Aryaeefar et al. 2022). The clinical evidence presented across these studies suggests that various medicinal plant interventions may offer therapeutic benefits through several introduced mechanisms. These include diuretic effects that increase urine volume and flow to help expel stones (Aryaeefar et al. 2022), antispasmodic or muscle-relaxant properties that could promote stone passage, direct effects on the stones, such as dissolution or preventing growth (Ansari et al. 2024), and positive modulatory impacts on urine chemistry, like increasing citrate (an inhibitor of stone formation) or decreasing calcium and oxalate (stone-forming substances) (Ansari et al. 2024). Furthermore, these effects include anti-inflammatory and antioxidant properties, which are claimed to play a role in protecting kidney tissue and inhibiting crystal aggregation (Alirezaei et al. 2023). A significant aspect emphasized in several studies is the favourable safety profile of these phytotherapeutic treatments, with few or no significant adverse effects mentioned and generally stable or improved markers of hepatic and renal function (Mehrabi et al. 2023).

Moreover, the clinical trials provided crucial evidence supporting the potential efficacy of herbal mixtures acting synergistically to manage urolithiasis. Several poly-herbal interventions demonstrated positive synergistic effects on stone clearance, whereas studies involving Mofatet powder (Ansari et al. 2024), a multi-herbal solution (Samandarian et al. 2023), Subap Plus (Patankar et al. 2020), an anti-urolithic herbal formula (Nisa & Astana 2019), and Kadalikshar (Rathod & Amilkanthwar 2015) stated statistically significant reductions in stone size or number, or increased expulsion rates compared to controls. Additionally, significant pain relief was observed with some of these multi-herbal preparations as well as the single ones, particularly *Nigella sativa*, which showed a more significant pain score reduction than tamsulosin (Shakeri et al. 2020) and Subap Plus (Patankar et al. 2020). Other than expulsion and pain, some included trials reported a positive influence on stone risk factors by altering urine composition, such as *Portulaca oleracea* increasing citrate and decreasing calcium (Alirezaei et al. 2023), or Mofatet powder decreasing calcium and increasing magnesium (Ansari et al. 2024). Nonetheless, other medicinal herbs, as *Nigella sativa* and *Alhagi maurorum*, demonstrated efficacy comparable to conventional pharmacological drugs (tamsulosin or hydrochlorothiazide, respectively) in reducing stone size and number (Mehrabi et al. 2023). Also, their safety profile or specific advantages in pain relief or overall efficacy (complete/partial response) were highlighted (Shakeri et al. 2020). Although these findings are promising and align with traditional uses, many of the studies displayed limitations, such as small sample sizes and the need for further research to fully understand the mechanisms, optimal dosages, and long-term effects (Alirezaei et al. 2023). However, the collective evidence as presented in the next SoF table (Table 5 suggests that herbal treatments offer a valuable and potentially well-tolerated approach as an alternative or complementary modality in urolithiasis management. This table provides a thorough summary of the main features and findings of each of the 14 included randomized controlled trials in terms of the purpose, design, sample size, intervention, comparator arms, primary outcomes evaluated, and key findings of each study. A quick comparison of the phytotherapeutic agents used, research environments, and clinical efficacy outcomes across various populations and methodologies is made easier by this tabulated synthesis, as it supports the discovery of common patterns in herbal interventions for the treatment of urolithiasis, and the full detailed data extraction table is provided in the supplementary materials.

**Table 5:** Clinical trials on herbal anti-urolithiasis therapy: summary of findings (SoF table)

Study ID	Study aim	Country	Study design	Intervention duration†	Sample size‡	Intervention vs. comparator	Outcomes/results*	Conclusion
Alirezaei et al. (2023)	To evaluate the effect of *Portulaca oleracea* powder on urinary and inflammatory markers in patients with nephrolithiasis and normal kidney function	Iran	Double-blinded placebo-controlled RCT	8 weeks	54 (herb 28 vs. placebo 26)	2 g of P. oleracea powder vs. 2 g of starch powder once per day	- Urine citrate: significantly higher in *P. oleracea* group vs. placebo (*P* < 0.01) - Urine calcium: significantly lower in *P. oleracea* group vs. placebo (*P* = 0.016) - ESR: significantly higher in *P. oleracea* group vs. placebo (*P* < 0.01)	*P. oleracea* may help prevent kidney stone formation by increasing urinary citrate and reducing urinary calcium. It may also influence inflammatory markers. Further studies are needed to confirm efficacy and safety
Ansari et al. (2024)	To evaluate the efficacy and safety of Mofatet powder (TPM formulation) in reducing calcium stone size, modulating urinary/serum parameters, and assessing nephro/hepatotoxicity	Iran	Double-blinded placebo-controlled RCT	5 weeks	49 (drug 26 vs. placebo 23)	500 mg Mofatet powder capsule vs. 500 mg roasted starch capsule twice per day	- Stone size: significant reduction in the drug group (*P* < 0.001), mean reduction ~ 3 mm (~ 61%) - Urine biochemistry significantly changed in the drug group vs. placebo: 5-week urine calcium decreased (*P* = 0.02); 5-week urinary magnesium increased (*P* < 0.001); 24-h urine specific gravity decreased (*P* = 0.015) - Serum calcium: significantly increased in the drug group (*P* < 0.001) - Safety: no significant adverse events reported	Mofatet powder appears safe and effective in reducing calcium-based stones and may exert a preventive effect by modulating urinary calcium and magnesium. Further trials with larger samples and longer follow-up are recommended to validate findings and explain mechanisms
Aryaeefar et al. (2022)	To evaluate the effect of *Alhagi maurorum* distillate on stone expulsion time and treatment tolerability in patients with ureteral stones	Iran	Single-blinded RCT	4 weeks	110 (herb 55 vs. control 55)	50 ml Alhagi maurorum distillate thrice per day vs. 0.4 mg tamsulosin capsule + 100 mg diclofenac suppository twice per day	- Stone expulsion time: 67% of patients in the intervention group passed stones by week 3 in a shorter duration (4 days) vs. control (*P* < 0.001) - Tolerability and safety: no adverse effects reported and high patient tolerance	*Alhagi maurorum* distillate significantly accelerated ureteral stone expulsion and was well-tolerated without adverse events. It may be used as adjunctive therapy for small ureteral stones. Further studies are needed to confirm efficacy, determine optimal dosage, and explore underlying mechanisms
Gao et al. (2021)	To evaluate the preventive effect and mechanism of the Huayu Jianpi Fangshi decoction (HJFD) from TCM in urolithiasis prevention focusing on modulating stone inhibitors/promoters	China	Double-blinded placebo-controlled RCT	4 weeks	57 (drug 26 vs. placebo 28)	HJFD decoction vs. placebo decoction twice per day	- Significant change favouring treatment group vs. control: citric acid increased (*P* < 0.01); urinary pH increased (*P* < 0.01); THP decreased (*P* < 0.05); calcium increased (*P* < 0.01) - No significant change in urinary magnesium, OPN, or ITIH3 (*P* > 0.05)	HJFD demonstrated potential in urolithiasis prevention by enhancing urinary stone inhibitors and reducing promoters. Further validation in clinical settings is needed
Kumari and Tukaram (2022)	To evaluate the effectiveness of Palasha Kshara and Ashmarihara Kwatha in managing urolithiasis and generate evidence-based support for Ayurvedic therapy	India	Open-labelled placebo-controlled RCT	8 weeks	39 (drug/A 20 vs. placebo/B 19)	500 mg Palasha Kshara capsule + 40 mL of *Ashmarihara Kwatha* thrice daily (Group A) vs. 500 mg granulated wheat capsule thrice a day (Group B)	- Symptom relief: significant improvement in pain (*P* < 0.00001) and urinary frequency (*P* = 0.0064); insignificant difference in haematuria (*P* = 0.61) and burning micturition (*P* = 0.96) - Stone metrics: significant reductions in stone size (*P* = 0.0005), number (*P* = 0.0333), and position (*P* = 0.0345) - Expulsion rates: Group A expelled 75% of ureteric and 29.7% of renal stones vs. 0% and 20% in the placebo - Safety: no safety concerns reported	The Ayurvedic combination (Palasha Kshara and Ashmarihara Kwatha) is a promising integrative option for urolithiasis management by showing superior efficacy over placebo in relieving symptoms, reducing stone burden, and promoting expulsion of small stones (< 10 mm)
Mehrabi et al. (2023)	To compare the efficacy of *Alhagi maurorum* extract vs. hydrochlorothiazide in eliminating kidney and ureteral stones and evaluate their impact on renal function	Iran	Open-labelled RCT	2 weeks	76 (herb 40 vs. drug 36)	500 mg *Alhagi maurorum* capsule twice per day vs. 50 mg Hydrochlorothiazide tablet once per day	- Both groups showed reduced stone size and number - No significant difference between groups in stone size (*P* = 0.314), number (*P* = 0.052), or renal markers - Stone clearance (> 70%) achieved in both groups - No significant adverse effects reported	*Alhagi maurorum* is a promising herbal alternative showing comparable efficacy to hydrochlorothiazide in reducing kidney and ureteral stones, with good tolerability and no adverse renal effects
Ardakani Movaghati et al. (2019)	To evaluate the efficacy and safety of Nigella sativa (black seed) in dissolving kidney stones, and its effects on urine/blood biochemical markers	Iran	Double-blinded placebo-controlled RCT	10 weeks	53 (herb 26 vs. placebo 27)	500 mg Black seed (*Nigella sativa*) capsule vs. 500 mg starch capsules twice per day	- Significant stone size reduction in the black seed group vs placebo (*P* < 0.05) - Complete stone excretion significantly higher (RR = 2.88; *P* = 0.035) - Urine pH improved (*P* = 0.002); no change in serum calcium - Black seed: 44.4% excretion, 51.8% size reduction vs. placebo: 15.3% excretion, 11.5% size reduction (*P* < 0.05)	*N. sativa* significantly enhanced stone reduction and expulsion compared to placebo, especially for smaller stones. It is safe and well-tolerated although further studies are needed for larger stones and long-term outcomes
Nisa and Astana (2019)	To compare the efficacy and safety of AHF vs. CPL in patients with urolithiasis, using stone size/number and liver/kidney function	Indonesia	Open-labelled RCT (end-blinded)	8 weeks	191 (AHF 97 vs CPL 94)	AHF (boiled in 1 L water for 15 min) twice per day vs. CPL capsule once per day	- Stone number significantly reduced in the AHF group at week 8 (*P* < 0.05) - No significant between-group difference in stone size reduction (*P* = 0.798) - VAS score (pain) did not differ significantly - No significant changes in liver/renal function (SGOT, SGPT, BUN, creatinine, potassium)	- AHF is safe and effective in treating urolithiasis and significantly reducing stone number better than CPL, though differences in size reduction and pain relief were not statistically significant
Patankar et al. (2020)	To evaluate the safety and efficacy of the herbal formulation Subap Plus (IP) in patients with asymptomatic renal calculi (4–9 mm)	India	Double-blinded placebo-controlled RCT	24 weeks	65 (herb 34 vs placebo 31)	Subap Plus (IP) 500 mg capsule vs. placebo capsule twice per day	- Significant reduction in stone surface area (*P* < 0.005) and density (HU) (*P* < 0.001) in treatment group vs. placebo - Higher stone expulsion rate in treatment group (*P* = 0.03) - Greater pain reduction based on VAS scores (*P* < 0.0001) - No major adverse events reported	Subap Plus showed anti-urolithiatic and analgesic effects by reducing stone size, density, and pain and improving expulsion. It may be considered for managing small, non-obstructive renal calculi and preventing recurrence
Rathod and Amilkanthwar (2015)	To evaluate the efficacy of Kadalikshar in reducing stone size, promoting expulsion, and alleviating urolithiasis-related symptoms	India	Double-blinded placebo-controlled RCT	4 weeks	60 (herb/trial 30 vs control/placebo 30)	500 mg Kadalikshar capsule vs. Gokshuradi Yog mixture twice per day	- Greater mean stone size reduction in the trial group (4.23 mm) vs. control (3.29 mm) over 28 days - Significant benefit in kidney stones (χ^2^ = 6.705, *P* < 0.05); not significant in ureteric/mixed stones - Higher stone resolution in the trial group: 34% dissolved, 23% expelled vs. 11% and 9% in controls, *P* < 0.05 - Greater symptom relief in trial group: pain (73% vs. 53%), burning micturition (80% vs. 65%), and haematuria (53% vs. 33%), *P* < 0.05	Kadalikshar was more effective than Gokshuradi Yog in reducing stone size, facilitating expulsion, and relieving urolithiasis-related symptoms. It may be a cost-effective and well-tolerated alternative in Ayurvedic management
Samandarian et al. (2023)	To evaluate the efficiency of an oral solution containing five herbal plant extracts in managing urolithiasis and its potential effects on kidney stones and 24-h urine indices	Iran	Single-blinded placebo-controlled RCT	4 weeks	54 (drug/herb 27 vs. placebo 27)	Herbal solution (*Tribulus terrestris*, *Urtica dioica*, *Adiantum capillus‑veneris*, *Stigma maydis* (corn silk), and *Cucumis melo*) vs. 60 drops placebo solution thrice per day	- Significant stone size reduction in intervention group vs. placebo (*P* = 0.049) - Stone number decreased within the treatment group (*P* = 0.009); between-group difference not significant (*P* = 0.093) - Higher complete stone expulsion in the drug group: 44.4% vs. 14.8% (*P* = 0.017) - Increased 24-h urine volume in the drug group (*P* = 0.003); no other significant urinary changes - Mild heartburn in two patients; no serious adverse events reported	The five-extract herbal solution demonstrated potential benefits in reducing stone size and enhancing expulsion with good tolerability. Though promising as an adjunct therapy, further studies with larger sample sizes and longer duration are required
Shakeri et al. (2020)	To compare efficacy and safety of oral *Peganum harmala* seed vs. tamsulosin in relieving pain and promoting expulsion of renal and ureteral stones	Iran	RCT	2 weeks	76 (herb/seed 40 vs. drug/tamsulosin 36)	50 mg/kg/d. *Peganum harmala* seed capsule once per day vs. 0.4 mg Tamsulosin capsule once per day	- No significant difference in stone size or number between groups (*P* > 0.05), though size reduction was numerically greater in *P. harmala* - Complete stone expulsion: 25% (*P. harmala*) vs. 38.9% (tamsulosin), *P* = 0.06 (not significant) - Pain scores decreased more significantly in *P. harmala* group (*P* = 0.002) - No serious adverse effects reported; one mild case of rash/itching	*Peganum harmala* was as effective as tamsulosin in managing urinary stones, with superior pain reduction and good tolerability. Larger, longer-term trials with optimized dose are necessary to better explore its multi-action profile
Shakeri et al. (2021)	To compare efficacy of *Nigella sativa* seeds vs. tamsulosin on expulsion and pain relief of ureteral and renal stones smaller than 10 mm	Iran	RCT	2 weeks	78 (herb/seed 39 vs. drug/tamsulosin 39)	1 gm *Nigella sativa* seed capsule twice per day vs. 0.4 mg Tamsulosin capsule once per day	- No significant difference in post-treatment stone size or number between groups (*P* > 0.05) - Pain significantly improved in both groups (*P* = 0.001), with greater reduction in the *Nigella sativa* group - Overall treatment efficacy higher in the *Nigella sativa* group with complete response of 41% vs. 23.1% and failure rate of 23.1% vs. 38.5% (*P* = 0.005) - No significant adverse effects reported	*Nigella sativa* may be a safe and effective alternative to tamsulosin, with better pain control and higher overall treatment response, despite no difference in stone size or number reduction
Vitkovskyy (2021)	To evaluate the effect of BNO 1040 herbal extract on improving ESWL outcomes and preventing early recurrence in patients with urolithiasis	Ukraine	Open-labelled RCT	24 weeks	150 (herb/main 75 vs. control 75)	2 tabs/50 drops BNO 1040 (Canephron® N.) thrice per day vs. standard care taken as instructed (Diet, ESWL, adequate fluid intake, antispasmodic drugs, painkillers /antispasmodics for pain, exercise therapy)	- Faster stone fragment elimination in the intervention BNO1040 group (9.81 vs. 12.23 days, *P* = 0.011) - Higher complete elimination by day 14 (94.7% vs. 76%, *P* = 0.042) - Lower leukocyturia (10.7% vs. 22.7%) and pain incidence (6.7% vs. 10.7%), though not statistically significant (*P* > 0.05) - Lower recurrence over 1 year (6.7% vs. 16%, *P* < 0.05) - No serious adverse events reported	BNO 1040 herbal extract appears safe and may improve fragment elimination and reduce recurrence post-ESWL. Further studies are needed to confirm long-term effects

*AHF* antiurolithic herbal formula, *CPL* commercial polyherbal lithotripsic, *HJFD* Huayu Jianpi Fangshi decoction, *HU* Hounsfield units, *TCM* Traditional Chinese Medicine, *TPM* Traditional Persian Medicine, *UL* urolithiasis

*All *P*-values refer to between-group comparisons unless otherwise specified. Significance is considered at *P* < 0.05

†Intervention duration is reported in weeks for consistency across studies. Where studies reported only the treatment period (not the full study duration), this was used

‡Sample sizes represent final analyzed numbers unless stated otherwise

### Quantitative evidence on herbal efficacy from meta-analysis

A meta-analysis was conducted to quantitatively evaluate the impact of herbal treatments on important clinical outcomes, especially changes in stone size and stone count, to complement the qualitative synthesis. These pooled studies consisted of only trials reporting sufficient statistical data, such as means, standard deviations, and sample sizes. More conclusive insights on the therapeutic consistency and direction of phytotherapy outcomes in kidney stone management were given by the visual and statistical summary of effect sizes across studies presented by the produced forest plots.

#### Stone size reduction

The quantitative synthesis of seven clinical trials evaluating the effect of herbal interventions on stone size revealed a pooled mean difference (MD) of − 1.72 mm (95% CI, − 2.33 to − 1.12), favouring the intervention groups over controls. This negative MD signifies that, on average, the stone size was significantly reduced in patients receiving phytotherapy compared to those who received placebo or standard care. Importantly, the analysis demonstrated no significant heterogeneity across studies after calculating the following parameters (Chi^2^ = 1.24, df = 6, *P* = 0.975; *I*^2^ = 0%), suggesting a high degree of consistency in treatment effect despite variations in herbal formulations and trial settings. Also, studies like Ansari et al. (Patankar et al. 2020) and Ardakani Movaghati et al. (2019) stated larger reductions in stone size, which substantially contributed to the overall pooled effect owing to their weight. Furthermore, the consistent direction and magnitude of these effects across geographically and methodologically diverse trials highlight the therapeutic potential of phytotherapy in stone dissolution or reduction modalities. The shown forest plot (Fig. 4) illustrates that most individual studies aligned on the left side of the non-significance (null) line, visually reinforcing the statistical finding of a reduction in stone burden with herbal intervention. The diamond representing the pooled estimate also does not cross the line of no effect, further confirming the robustness of this conclusion.

#### Stone number reduction

Similarly, the meta-analysis of four trials measuring the outcome of stone number reduction showed a pooled mean difference of − 0.10 (95% CI, − 0.35 to 0.14) in favour of herbal interventions, as depicted in this forest plot (Fig. 5). Although this effect was not statistically significant at the conventional 0.05 significance level, it still suggests a trend toward a reduction in the number of stones in patients treated with herbal remedies compared to control groups. The strength of this analysis lies in the absence of heterogeneity across included studies, where the calculated parameters are as follows (Chi^2^ = 0.10, df = 3, *P* = 0.992; *I*^2^ = 0%), indicating that the intervention effect was consistent across different formulations and methodologies. Notably, Shakeri et al. (2021) and Samandarian et al. (2023) reported potential reductions in stone counts, with Shakeri et al. contributing the largest weight due to a relatively large sample size and lower variance. While the overall effect did not reach statistical significance, the direction of effect in most studies supported the promising benefit of herbal therapy in reducing the recurrence or burden of multiple stones, mostly through mechanisms such as diuresis, antispasmodic action, or improved urinary flow.

#### Clinical implications

These findings collectively highlight the evolving role of phytotherapy as a promising modality or adjunctive therapy in the management of urolithiasis. Besides, the robust and consistent evidence for the already addressed outcome, stone size reduction, suggests that certain herbal formulations may serve as effective non-invasive options, either as adjuncts or alternatives to conventional pharmacological therapy. Although the evidence for stone number reduction is less conclusive, the observed trends and high consistency across studies warrant further investigation, particularly in larger, longer-term trials. Also, while these included trials yielded directionally consistent results, the high risk of bias observed in the domains of missing outcomes and intervention deviations suggests that these findings should be interpreted with caution. So, clinicians and researchers should balance both the mechanistic rationale and clinical efficacy against the overall certainty of evidence when evaluating herbal interventions, especially given their shown potential to improve patient outcomes with a favourable safety and cost profile.

## Conclusions

Urolithiasis, or kidney stone disease, remains a common and recurrent condition that imposes significant physical discomfort and healthcare burden globally. It refers to the formation of hard mineral deposits anywhere along the urinary tract, most frequently in the kidneys, where these stones develop when minerals like calcium and oxalate become highly concentrated in urine, often due to inadequate hydration, dietary factors, or genetic predisposition. Its recurrence is a significant concern, with rates reaching up to 50% within 10 years, especially in individuals with risk factors like family history, metabolic disorders, or previous stone episodes. While conventional preventive measures, implying increased fluid intake, dietary adjustments (such as reducing salt and animal protein, ensuring adequate dietary calcium, and limiting high-oxalate foods), and pharmacotherapy, represent the cornerstone of management, the high recurrence rates highlight the need for integrative, sustainable solutions, since kidney stones can cause severe pain, urinary symptoms, and, in some cases, complications such as obstruction or infection. This systematic review has consolidated evidence from 14 clinical trials evaluating herbal interventions, many of which demonstrated statistically significant benefits in reducing stone size, number, and recurrence risk, as well as in improving urinary biochemical parameters. Several single-herb and polyherbal preparations, including *Nigella sativa*, *Portulaca oleracea*, *and Alhagi maurorum*, along with traditional formulations from Ayurveda and Traditional Persian Medicine, were found to be both efficacious and well-tolerated, offering potential adjunctive strategies in both preventive and therapeutic contexts. Hence, nutraceuticals, including certain dietary plants and phytochemicals, have shown promise in reducing stone formation by exerting multifactorial mechanisms, involving modulation of urinary pH, reduction of stone-forming ions, inhibition of crystallization, antioxidant activity, diuretic effects, and support of urinary health. Importantly, most studies reported no serious adverse events, supporting their safety profile. Collectively, these findings advocate for a holistic, patient-centered approach that integrates evidence-based herbal interventions with conventional medical management and lifestyle modifications. While findings from the included clinical trials indicate encouraging effects of herbarbal interventions on stone prevention and treatment, the evidence remains emerging and requires further well-designed, large-scale trials to confirm optimal dosing, long-term efficacy, and mechanisms of action, as well as ensure rigorous clinical validation. Nonetheless, merging clinically supported phytotherapy into standard urolithiasis care may help lower recurrence rates, enhance urinary health in stone formers, improve patient quality of life, and offer cost-effective options, especially in low-resource settings.

## Data Availability

All source data for this work (or generated in this study) are available upon reasonable request.

## References

[CR1] Abdel Raheem A, Alowidah I, Hagras A, Gameel T, Ghaith A, Elghiaty A, Althakafi S, Al-Mousa M, Alturki M (2021) Laparoscopic ureterolithotomy for large proximal ureteric stones: surgical technique, outcomes and literature review. Asian J Endosc Surg 14:241–249 32875735 10.1111/ases.12861

[CR2] Adomako E, Moe OW (2020) Uric acid and urate in urolithiasis: the innocent bystander, instigator, and perpetrator. Seminars in nephrology. Elsevier, pp 564–573 10.1016/j.semnephrol.2020.12.003 PMC8127876 33678311

[CR3] Aggarwal KP, Narula S, Kakkar M, Tandon C (2013) Nephrolithiasis: molecular mechanism of renal stone formation and the critical role played by modulators. BioMed Res Int 2013:292953 24151593 10.1155/2013/292953 PMC3787572

[CR4] Ahmed MZ, Ahmed N, Asif M, Shahid MW, Channa AA, Cheema NA (2022) To compare the efficacy of nifedipine and tamsulosin 0.4 mg in expulsion of lower ureteric stones. Pak J Med Health Sci 16:1040–1040

[CR5] Aksenov LI, Streeper NM, Scales CD Jr (2024) Leveraging behavioral modification technology for the prevention of kidney stones. Curr Opin Urol 34:14–19 10.1097/MOU.0000000000001142 PMC10842369 37962162

[CR6] Alexander RT (2023) Kidney stones, hypercalciuria, and recent insights into proximal tubule calcium reabsorption. Curr Opin Nephrol Hypertens 32:359–365 37074688 10.1097/MNH.0000000000000892

[CR7] Alirezaei A, Toudeshki KK, Nouri SB, Fazeli SA, Hatami F, Miladipour A, Montazeri-Ghominezhad SP (2023) The effects of *Portulaca oleracea* extract on 24-hour urine indices in patients with renal stone: a double-blind randomized placebo-controlled clinical trial. J Ren Inj Prev 12:e32240–e32240

[CR8] Allam EA (2024) Urolithiasis unveiled: pathophysiology, stone dynamics, types, and inhibitory mechanisms: a review. Afr J Urol 30:34

[CR9] Ansari R, Karimzade I, Nimrouzi M, Ezatzadegan S, Hosseini MM, Zarshenas MM (2024) Safety and efficacy of a polyherbal formulation from traditional Persian medicine in patients with calcium kidney stones: a randomized, double-blinded clinical trial. J Res Med Sci 29:12 38524751 10.4103/jrms.jrms_670_22 PMC10956567

[CR10] Arabski M, Stabrawa I, Kubala-Kukuś A, Gałczyńska K, Banaś D, Piskorz Ł, Forma E, Bryś M, Różański W, Lipiński M (2019) The correlation of crystalline and elemental composition of urinary stones with a history of bacterial infections: TXRF, XRPD and PCR-DGGE studies. Eur Biophys J 48:111–118 30483831 10.1007/s00249-018-1338-7 PMC6330562

[CR11] Ardakani Movaghati MR, Yousefi M, Saghebi SA, Sadeghi Vazin M, Iraji A, Mosavat SH (2019) Efficacy of black seed (*Nigella sativa* L.) on kidney stone dissolution: a randomized, double-blind, placebo-controlled, clinical trial. Phytother Res 33:1404–1412 30873671 10.1002/ptr.6331

[CR12] Aryaeefar MR, Khakbaz A, Akbari S, Movahedi A, Gazerani A, Bidkhori M, Moeini V (2022) Effect of *Alhagi maurorum* distillate on ureteral stone expulsion: a single-blind randomized trial. J Herb Med 34:100567

[CR13] Balawender K, Łuszczki E, Mazur A, Wyszyńska J (2024) The multidisciplinary approach in the management of patients with kidney stone disease—a state-of-the-art review. Nutrients 16:1932 38931286 10.3390/nu16121932 PMC11206918

[CR14] Bargagli M, Trelle S, Bonny O, Fuster DG (2024) Thiazides for kidney stone recurrence prevention. Curr Opin Nephrol Hypertens 33:427–432 38606682 10.1097/MNH.0000000000000990 PMC11139243

[CR15] Borghi L, Meschi T, Amato F, Briganti A, Novarini A, Giannini A (1996) Urinary volume, water and recurrences in idiopathic calcium nephrolithiasis: a 5-year randomized prospective study. J Urol 155:839–843 8583588

[CR16] Bouchalakis A, Somani BK, Lima E, Rassweiler-Seyfried M-C, Mamoulakis C, Tokas T (2024) Navigation systems and 3D imaging in percutaneous nephrolithotripsy: improving outcomes and safety. Curr Opin Urol 34:105–109 37889519 10.1097/MOU.0000000000001136

[CR17] Brandel MG, Lin C, Hennel D, Khazen O, Pilitsis JG, Ben-Haim S (2022) Mindfulness meditation in the treatment of chronic pain. Neurosurg Clin N Am 33:275–279 35718396 10.1016/j.nec.2022.02.005

[CR18] Breeggemann MC, Harris PC, Lieske JC, Tasian GE, Wood KD (2024) The evolving role of genetic testing in monogenic kidney stone disease: spotlight on primary hyperoxaluria. J Urol 212:649–659 39093847 10.1097/JU.0000000000004147

[CR19] Brinkman JE, Large T, Nottingham CU, Stoughton C, Krambeck AE (2021) Clinical and metabolic correlates of pure stone subtypes. J Endourol 35:1555–1562 33573466 10.1089/end.2020.1035

[CR20] Caglayan A, Horsanali MO, Kocadurdu K, Ismailoglu E, Guneyli S (2022) Deep learning model-assisted detection of kidney stones on computed tomography. Int Braz J Urol 48:830–839 35838509 10.1590/S1677-5538.IBJU.2022.0132 PMC9388181

[CR21] Cao C, Li F, Ding Q, Jin X, Tu W, Zhu H, Sun M, Zhu J, Yang D, Fan B (2024) Potassium sodium hydrogen citrate intervention on gut microbiota and clinical features in uric acid stone patients. Appl Microbiol Biotechnol 108:51 38183479 10.1007/s00253-023-12953-y PMC10771603

[CR22] Cassell III A, Jalloh M, Ndoye M, Mbodji M, Gaye O, Thiam NM, Diallo A, Labou I, Niang L, Gueye S (2020) Surgical management of urolithiasis of the upper tract–current trend of endourology in Africa. Res Rep Urol 225–238 10.2147/RRU.S257669 PMC7352378 32754452

[CR23] Cauni V-M, Dragutescu M, Mihai B, Gorecki G-P, Ples L, Sima R-M, Persu C (2023) Mini-perc for renal stones—a single center experience and literature review. Diagnostics 13:1083 36980392 10.3390/diagnostics13061083 PMC10047343

[CR24] Chen T, Zhu Z, Du J (2021) Efficacy of intercostal nerve block for pain control after percutaneous nephrolithotomy: a systematic review and meta-analysis. Front Surg 8:623605 33585552 10.3389/fsurg.2021.623605 PMC7876386

[CR25] Chewcharat A, Curhan G (2021) Trends in the prevalence of kidney stones in the United States from 2007 to 2016. Urolithiasis 49:27–39 32870387 10.1007/s00240-020-01210-w

[CR26] Coe FL, Parks JH, Asplin JR (1992) The pathogenesis and treatment of kidney stones. N Engl J Med 327:1141–1152 1528210 10.1056/NEJM199210153271607

[CR27] Courbebaisse M, Travers S, Bouderlique E, Michon-Colin A, Daudon M, De Mul A, Poli L, Baron S, Prot-Bertoye C (2023) Hydration for adult patients with nephrolithiasis: specificities and current recommendations. Nutrients 15:4885 38068743 10.3390/nu15234885 PMC10708476

[CR28] Cui Y, Chen J, Zeng F, Liu P, Hu J, Li H, Li C, Cheng X, Chen M, Li Y (2019) Tamsulosin as a medical expulsive therapy for ureteral stones: a systematic review and meta-analysis of randomized controlled trials. J Urol 201:950–955 30694932 10.1097/JU.0000000000000029

[CR29] Cupisti A, Meola M, D’Alessandro C, Bernabini G, Pasquali E, Carpi A, Barsotti G (2007) Insulin resistance and low urinary citrate excretion in calcium stone formers. Biomed Pharmacother 61:86–90 17184967 10.1016/j.biopha.2006.09.012

[CR30] Curhan GC, Willett WC, Rimm EB, Speizer FE, Stampfer MJ (1998) Body size and risk of kidney stones. J Am Soc Nephrol 9:1645–1652 9727373 10.1681/ASN.V991645

[CR31] D’Ambrosio V, Capolongo G, Goldfarb D, Gambaro G, Ferraro PM (2022) Cystinuria: an update on pathophysiology, genetics, and clinical management. Pediatr Nephrol. 10.1007/s00467-021-05342-y 34812923 10.1007/s00467-021-05342-y

[CR32] Das P, Gupta G, Velu V, Awasthi R, Dua K, Malipeddi H (2017) Formation of struvite urinary stones and approaches towards the inhibition—a review. Biomed Pharmacother 96:361–370 29028588 10.1016/j.biopha.2017.10.015

[CR33] Daudon M, Jungers P (2004) Drug-induced renal calculi: epidemiology, prevention and management. Drugs 64:245–275 14871169 10.2165/00003495-200464030-00003

[CR34] Dawson-Hughes B, Harris SS, Rasmussen H, Song L, Dallal GE (2004) Effect of dietary protein supplements on calcium excretion in healthy older men and women. J Clin Endocrinol Metab 89:1169–1173 15001604 10.1210/jc.2003-031466

[CR35] Dawson CH, Tomson CR (2012) Kidney stone disease: pathophysiology, investigation and medical treatment. Clin Med 12:467 10.7861/clinmedicine.12-5-467 PMC4953772 23101150

[CR36] De Lorenzis E, Zanetti SP, Boeri L, Montanari E (2022) Is there still a place for percutaneous nephrolithotomy in current times? J Clin Med 11:5157 36079083 10.3390/jcm11175157 PMC9457409

[CR37] DiBianco JM, Ghani KR (2021) Precision stone surgery: current status of miniaturized percutaneous nephrolithotomy. Curr Urol Rep 22:1–11 10.1007/s11934-021-01042-0 33576896

[CR38] Dobrek Ł (2020) Kidney stone disease with special regard to drug-induced kidney stones—a contemporary synopsis. Wiad Lek 73:2031–2039 33148855

[CR39] Dolin DJ, Asplin JR, Flagel L, Grasso M, Goldfarb DS (2005) Effect of cystine-binding thiol drugs on urinary cystine capacity in patients with cystinuria. J Endourol 19:429–432 15865542 10.1089/end.2005.19.429

[CR40] Dönmez S, Tekin E, Erdem AB, Şener A, Yilmaz M, Ünal E (2024) Efficacy comparison of ibuprofen 400 mg and 800 mg in the treatment of renal colic: prospective randomized double-blind clinical study. Bezmialem Science. 10.14235/bas.galenos.2024.78095

[CR41] Edvardsson VO, Indridason OS, Haraldsson G, Kjartansson O, Palsson R (2013) Temporal trends in the incidence of kidney stone disease. Kidney Int 83:146–152 22992468 10.1038/ki.2012.320

[CR42] Edvardsson VO, Ingvarsdottir SE, Palsson R, Indridason OS (2018) Incidence of kidney stone disease in Icelandic children and adolescents from 1985 to 2013: results of a nationwide study. Pediatr Nephrol 33:1375–1384 29626242 10.1007/s00467-018-3947-x

[CR43] Escobar Monroy R, Proietti S, De Leonardis F, Gisone S, Scalia R, Mongelli L, Gaboardi F, Giusti G (2025) Complications in percutaneous nephrolithotomy. Complications 2:5

[CR44] Evan A, Lingeman J, Coe F, Worcester E (2006) Randall’s plaque: pathogenesis and role in calcium oxalate nephrolithiasis. Kidney Int 69:1313–1318 16614720 10.1038/sj.ki.5000238

[CR45] Evan AP (2010) Physiopathology and etiology of stone formation in the kidney and the urinary tract. Pediatr Nephrol 25:831–841 19198886 10.1007/s00467-009-1116-y PMC2839518

[CR46] Evan AP, Worcester EM, Coe FL, Williams J, Lingeman JE (2015) Mechanisms of human kidney stone formation. Urolithiasis 43:19–32 25108546 10.1007/s00240-014-0701-0 PMC4285570

[CR47] Felizio J, Atmoko W (2022) Medical management of kidney stones: a review. Bali Med J 11:127–136

[CR48] Fink HA, Wilt TJ, Eidman KE, Garimella PS, MacDonald R, Rutks IR, Brasure M, Kane RL, Ouellette J, Monga M (2013) Medical management to prevent recurrent nephrolithiasis in adults: a systematic review for an American College of Physicians clinical guideline. Ann Intern Med 158:535–543 23546565 10.7326/0003-4819-158-7-201304020-00005

[CR49] Fontenelle LF, Sarti TD (2019) Kidney stones: treatment and prevention. Am Fam Physician 99:490–496 30990297

[CR50] Freiwald J, Magni A, Fanlo-Mazas P, Paulino E, Sequeira de Medeiros L, Moretti B, Schleip R, Solarino G (2021) A role for superficial heat therapy in the management of non-specific, mild-to-moderate low back pain in current clinical practice: a narrative review. Life 11:780 34440524 10.3390/life11080780 PMC8401625

[CR51] Gambaro G, Croppi E, Coe F, Lingeman J, Moe O, Worcester E, Buchholz N, Bushinsky D, Curhan GC, Ferraro PM (2016) Metabolic diagnosis and medical prevention of calcium nephrolithiasis and its systemic manifestations: a consensus statement. J Nephrol 29:715–734 27456839 10.1007/s40620-016-0329-y PMC5080344

[CR52] Gao X-S, Pan J, Pang R, Liu B, Song S-Q (2021) Mechanism of Huayu Jianpi Fangshi decoction in urolithiasis prevention: a randomized trial. Ann Palliat Med 10:4320327–4324327 10.21037/apm-20-2295 33832307

[CR53] Goldfarb DS, Avery AR, Beara-Lasic L, Duncan GE, Goldberg J (2019) A twin study of genetic influences on nephrolithiasis in women and men. Kidney Int Rep 4:535–540 30993229 10.1016/j.ekir.2018.11.017 PMC6451147

[CR54] Goldfarb DS, MacDonald PA, Gunawardhana L, Chefo S, McLean L (2013) Randomized controlled trial of febuxostat versus allopurinol or placebo in individuals with higher urinary uric acid excretion and calcium stones. Clin J Am Soc Nephrol 8:1960–1967 23929928 10.2215/CJN.01760213 PMC3817901

[CR55] Griffith DP (1978) Struvite stones. Kidney Int 13:372–382 351265 10.1038/ki.1978.55

[CR56] Griffith DP, Musher Dá (1976) Urease: principal cause of infection stones. Urolithiasis research. Springer, pp 451–454

[CR57] Guittet C, Roussel-Maupetit C, Manso-Silván M, Guillaumin F, Vandenhende F, Granier L (2020) Innovative prolonged-release oral alkalising formulation allowing sustained urine pH increase with twice daily administration: randomised trial in healthy adults. Sci Rep 10:13960 32811843 10.1038/s41598-020-70549-2 PMC7434908

[CR58] Güler Y, Erbin A, Ozmerdiven G, Yazici O (2020) Comparison of retrograde intrarenal surgery and laparoscopic surgery in the treatment of proximal ureteral and renal pelvic stones greater than 15 mm. Folia Med 62:490–496 10.3897/folmed.62.e48934 33009767

[CR59] Halbritter J (2021) Genetics of kidney stone disease—polygenic meets monogenic. Nephrol Ther 17:S88–S94 10.1016/j.nephro.2020.02.003 33910705

[CR60] Halbritter J, Seidel A, Müller L, Schönauer R, Hoppe B (2018) Update on hereditary kidney stone disease and introduction of a new clinical patient registry in Germany. Front Pediatr 6:47 29564324 10.3389/fped.2018.00047 PMC5850730

[CR61] Hanchanale VS, Myatt A, Somani B, Nabi G, Biyani CS (2015) Citrate salts for preventing and treating calcium containing kidney stones in adults. Cochrane Database Syst Rev 10:CD010057 10.1002/14651858.CD010057.pub2 PMC9578669 26439475

[CR62] Hasan O, Reed A, Shahait M, Crivellaro S, Dobbs RW (2023) Robotic surgery for stone disease. Curr Urol Rep 24:127–133 36394772 10.1007/s11934-022-01131-8

[CR63] Heers H, Stay D, Wiesmann T, Hofmann R (2022) Urolithiasis in Germany: trends from the national DRG database. Urol Int 106:589–595 34883491 10.1159/000520372 PMC9248299

[CR64] Heilberg IP, Goldfarb DS (2013) Optimum nutrition for kidney stone disease. Adv Chronic Kidney Dis 20:165–174 23439376 10.1053/j.ackd.2012.12.001

[CR65] Hoffman A, Braun MM, Khayat M (2021) Kidney disease: kidney stones. FP Essent 509:33–38 34643363

[CR66] Howles SA, Thakker RV (2020) Genetics of kidney stone disease. Nat Rev Urol 17:407–421 32533118 10.1038/s41585-020-0332-x

[CR67] Hyams ES, Matlaga BR (2014) Economic impact of urinary stones. Transl Androl Urol 3:278 26816777 10.3978/j.issn.2223-4683.2014.07.02 PMC4708578

[CR68] Jiang P, Xie L, Arada R, Patel RM, Landman J, Clayman RV (2021) Qualitative review of clinical guidelines for medical and surgical management of urolithiasis: consensus and controversy 2020. J Urol 205:999–1008 33284671 10.1097/JU.0000000000001478

[CR69] Jing Q, Liu F, Yuan X, Zhang X, Cao X (2024) Clinical comparative study of single-use and reusable digital flexible ureteroscopy for the treatment of lower pole stones: a retrospective case-controlled study. BMC Urol 24:149 39026274 10.1186/s12894-024-01541-5 PMC11256421

[CR70] Jones P, Karim Sulaiman S, Gamage KN, Tokas T, Jamnadass E, Somani BK (2021) Do lifestyle factors including smoking, alcohol, and exercise impact your risk of developing kidney stone disease? Outcomes of a systematic review. J Endourol 35:1–7 32808537 10.1089/end.2020.0378

[CR71] Joshi A, Tallman JE, Calvert JK, Brewer T, Miller NL, Yang L, Asplin JR, Hsi RS (2021) Complementary and alternative medicine use in first-time and recurrent kidney stone formers. Urology 156:58–64 34293376 10.1016/j.urology.2021.05.084

[CR72] Juliebø-Jones P, Keller EX, Haugland JN, Æsøy MS, Beisland C, Somani BK, Ulvik Ø (2023) Advances in ureteroscopy: new technologies and current innovations in the era of tailored endourological stone treatment (TEST). J Clin Urol 16:190–198

[CR73] Katkam N, Beddhu S (2024) Steps for stopping kidney stones: physical activity triumphant over genetics. pp S0272–6386 (0224) 00893-X 10.1053/j.ajkd.2024.07.001 PMC11898788 39162673

[CR74] Khan SR (2013) Reactive oxygen species as the molecular modulators of calcium oxalate kidney stone formation: evidence from clinical and experimental investigations. J Urol 189:803–811 23022011 10.1016/j.juro.2012.05.078 PMC5683176

[CR75] Khan SR, Canales BK, Dominguez-Gutierrez PR (2021) Randall’s plaque and calcium oxalate stone formation: role for immunity and inflammation. Nat Rev Nephrol 17:417–433 33514941 10.1038/s41581-020-00392-1

[CR76] Kronenberg P, Cerrato C, Juliebø-Jones P, Herrmann T, Tokas T, Somani BK (2023) Advances in lasers for the minimally invasive treatment of upper and lower urinary tract conditions: a systematic review. World J Urol 41:3817–3827 37906263 10.1007/s00345-023-04669-5

[CR77] Kumari M, Tukaram D (2022) Management of mutrashmari (urolithiasis) with Palasha Kshara and Ashmarihara Kwatha: an open-labelled placebo-controlled clinical trial. AYU (Int Quart J Res Ayurved) 43:54–59 10.4103/ayu.AYU_225_19 PMC10468020 37655175

[CR78] Kustov A, Moryganov M, Strel'nikov A, Zhuravleva N, Airapetyan A (2016) Quantitative mineralogical analyzes of kidney stones and diagnosing metabolic disorders in female patients with calcium oxalate urolithiasis. Urologiia 11–15 28247696

[CR79] Lei J, Huang K, Dai Y, Yin G (2023) Evaluating outcomes of patient-centered enhanced recovery after surgery (ERAS) in percutaneous nephrolithotomy for staghorn stones: an initial experience. Front Surg 10:1138814 37025266 10.3389/fsurg.2023.1138814 PMC10071039

[CR80] Leslie S, Bashir K (2024) Hypocitraturia and renal calculi. StatPearls

[CR81] Li D-f, Gao Y-l, Liu H-c, Huang X-c, Zhu R-f, Zhu C-t (2020) Use of thiazide diuretics for the prevention of recurrent kidney calculi: a systematic review and meta-analysis. J Transl Med 18:1–12 32111248 10.1186/s12967-020-02270-7 PMC7048029

[CR82] Liu M, Hou J, Xu F, Du H, Liu J, Li N (2023a) Minimally invasive nephrolithotomy versus retrograde intrarenal surgery in surgical management of lower calyceal stones: a systematic review with meta-analysis. Int J Surg 109:1481–1488 37037590 10.1097/JS9.0000000000000394 PMC10389464

[CR83] Liu M, Wu J, Gao M, Li Y, Xia W, Zhang Y, Chen J, Chen Z, Zhu Z, Chen H (2023b) Lifestyle factors, serum parameters, metabolic comorbidities, and the risk of kidney stones: a Mendelian randomization study. Front Endocrinol 14:1240171 10.3389/fendo.2023.1240171 PMC10560039 37810889

[CR84] Liu Y, Chen Y, Liao B, Luo D, Wang K, Li H, Zeng G (2018) Epidemiology of urolithiasis in Asia. Asian J Urol 5:205–214 30364478 10.1016/j.ajur.2018.08.007 PMC6197415

[CR85] Maalouf NM (2011) Metabolic syndrome and the genesis of uric acid stones. J Ren Nutr 21:128–131 21195936 10.1053/j.jrn.2010.10.015 PMC3053068

[CR86] Mao W, Zhang L, Sun S, Wu J, Zou X, Zhang G, Chen M (2022) Physical activity reduces the effect of high body mass index on kidney stones in diabetes participants from the 2007–2018 NHANES cycles: a cross-sectional study. Front Public Health 10:936552 35844866 10.3389/fpubh.2022.936552 PMC9283863

[CR87] Mazzucchi E, Marchini GS, Berto FCG, Denstedt J, Danilovic A, Vicentini FC, Torricelli FCM, Battagello CA, Srougi M, Nahas WC (2022) Single-use flexible ureteroscopes: update and perspective in developing countries. A narrative review. Int Braz J Urol 48:456–467 34786927 10.1590/S1677-5538.IBJU.2021.0475 PMC9060176

[CR88] Mehrabi S, Beigi P, Salehpour Z (2023) Comparison of the effect of hydroalcholic extract of *alhagi maurorum* and hydrochlorothiazide on excretion of 4–10 mm kidney and ureteral stones in adults: a randomized prospective study. Adv Pharmacol Pharm Sci 2023:6624981 37609006 10.1155/2023/6624981 PMC10442181

[CR89] Menon M (1993) A prospective study of dietary calcium and other nutrients and the risk of symptomatic kidney stones. J Urol 150:563–564 8326599

[CR90] Michibata U, Maruyama M, Tanaka Y, Yoshimura M, Yoshikawa HY, Takano K, Furukawa Y, Momma K, Tajiri R, Taguchi K (2024) The impact of crystal phase transition on the hardness and structure of kidney stones. Urolithiasis 52:57 38563829 10.1007/s00240-024-01556-5 PMC10987347

[CR91] Mousavi A, Takele R, Limbrick Ba, Thaker KN, Scotland KB (2024) Oral dissolution therapy of uric acid stones: a systematic review. Soc Int Urol J 5:284–299

[CR92] Moussa M, Papatsoris AG, Abou Chakra M, Moussa Y (2020) Update on cystine stones: current and future concepts in treatment. Intractable Rare Dis Res 9:71–78 32494553 10.5582/irdr.2020.03006 PMC7263987

[CR93] Nelli MAMLF (2004) Epidemiology of nephrolithiasis today. Urol Int 72:1–5 15133324 10.1159/000076582

[CR94] Nevo A, Levi O, Sidi A, Tsivian A, Baniel J, Margel D, Lifshitz D (2020) Patients treated for uric acid stones reoccur more often and within a shorter interval compared to patients treated for calcium stones. Can Urol Assoc J 14:E555 32520701 10.5489/cuaj.6259 PMC7673824

[CR95] Nisa U, Astana PRW (2019) Evaluation of antiurolithic herbal formula for urolithiasis?: a randomized open label clinical study. Asian J Pharma Clin Res 12:88–93

[CR96] Okumura N, Tsujihata M, Momohara C, Yoshioka I, Suto K, Nonomura N, Okuyama A, Takao T (2013) Diversity in protein profiles of individual calcium oxalate kidney stones. PLoS ONE 8:e68624 23874695 10.1371/journal.pone.0068624 PMC3706363

[CR97] Özdemir M, Çiğşar G, Bağcioğlu M, Çiftçi H, Gunal E (2023) Comparison of the analgesic effects of intravenous dexketoprofen, ibuprofen and fentanyl in patients suffering from renal colic pain in the emergency department. Eurasian J Emerg Med 22(1):18–23

[CR98] Pak CY, Poindexter JR, Adams-Huet B, Pearle MS (2003) Predictive value of kidney stone composition in the detection of metabolic abnormalities. Am J Med 115:26–32 12867231 10.1016/s0002-9343(03)00201-8

[CR99] Palsson R, Indridason OS, Edvardsson VO, Oddsson A (2019) Genetics of common complex kidney stone disease: insights from genome-wide association studies. Urolithiasis 47:11–21 30523390 10.1007/s00240-018-1094-2

[CR100] Paludo AdO, Gorgen ARH, Araldi M, Tavares PM, Batezini NS, Rosito TE (2020) Laparoscopic pielolitotomy: an option for the management of pelvic kidney stones. Int Braz J Urol 46:489–490 32167729 10.1590/S1677-5538.IBJU.2019.0148 PMC7088487

[CR101] Patankar SB, Mujumdar A, Bernard F, Supriya P (2020) Safety and efficacy of an herbal formulation in patients with renal calculi-a 28 week, randomized, double-blind, placebo-controlled, parallel group study. J Ayurveda Integr Med 11:62–67 30709687 10.1016/j.jaim.2018.08.001 PMC7125359

[CR102] Patel N, Brown RD, Sarkissian C, De S, Monga M (2017) Quality of life and urolithiasis: the patient-reported outcomes measurement information system (PROMIS). Int Braz J Urol 43:880–886 28792186 10.1590/S1677-5538.IBJU.2016.0649 PMC5678519

[CR103] Pinheiro VB, Baxmann AC, Tiselius H-G, Heilberg IP (2013) The effect of sodium bicarbonate upon urinary citrate excretion in calcium stone formers. Urology 82:33–37 23602798 10.1016/j.urology.2013.03.002

[CR104] Prezioso D, Strazzullo P, Lotti T, Bianchi G, Borghi L, Caione P, Carini M, Caudarella R, Gambaro G, Gelosa M (2015) Dietary treatment of urinary risk factors for renal stone formation. A review of CLU working group. Arch Ital Urol Androl 87:105–120 26150027 10.4081/aiua.2015.2.105

[CR105] Qin P, Zhang D, Huang T, Fang L, Cheng Y (2022) Comparison of mini percutaneous nephrolithotomy and standard percutaneous nephrolithotomy for renal stones> 2cm: a systematic review and meta-analysis. Int Braz J Urol 48:637–648 34786926 10.1590/S1677-5538.IBJU.2021.0347 PMC9306366

[CR106] Rahman IA, Nusaly IF, Syahrir S, Nusaly H, Mansyur MA (2021) Association between metabolic syndrome components and the risk of developing nephrolithiasis: a systematic review and Bayesian meta-analysis. F1000Res 10:104 34804491 10.12688/f1000research.28346.1 PMC8577060

[CR107] Rammah AM, Khaled F, Zamel S, Elkady A, Morsy S, Abdelwahab M (2025) The efficacy of flexible ureteroscopy for large volume stones and hazards of ureteral access sheath usage: a prospective randomized study. Arab J Urol 23:62–69 39776554 10.1080/20905998.2024.2398378 PMC11703511

[CR108] Ranjan SK, Mittal A, Mirza AA, Kumar S, Panwar VK, Navriya S, Mandal AK, Mammen KJ (2023) Metabolic evaluation of first-time uncomplicated renal stone formers: a prospective study. Curr Urol 17:36–40 37692144 10.1097/CU9.0000000000000169 PMC10487292

[CR109] Rathod R, Amilkanthwar R (2015) The effect of Kadalikshar in the management of mutrashmari (urolithiasis). Int J Res Ayurveda Pharm. 10.7897/2277-4343.06363

[CR110] Ratkalkar VN, Kleinman JG (2011) Mechanisms of stone formation. Clin Rev Bone Miner Metab 9:187–197 22229020 10.1007/s12018-011-9104-8 PMC3252394

[CR111] Rimer JD, Kolbach-Mandel AM, Ward MD, Wesson JA (2017) The role of macromolecules in the formation of kidney stones. Urolithiasis 45:57–74 27913854 10.1007/s00240-016-0948-8 PMC5253101

[CR112] Rodger F, Roditi G, Aboumarzouk OM (2018) Diagnostic accuracy of low and ultra-low dose CT for identification of urinary tract stones: a systematic review. Urol Int 100:375–385 29649823 10.1159/000488062

[CR113] Romero V, Akpinar H, Assimos DG (2010) Kidney stones: a global picture of prevalence, incidence, and associated risk factors. Rev Urol 12:e86 20811557 PMC2931286

[CR114] Rule AD, Bergstralh EJ, Melton LJ III, Li X, Weaver AL, Lieske JC (2009) Kidney stones and the risk for chronic kidney disease. Clin J Am Soc Nephrol 4:804–811 10.2215/CJN.05811108 PMC2666438 19339425

[CR115] Ryan LE, Ing SW (2018) Idiopathic hypercalciuria: can we prevent stones and protect bones. Cleve Clin J Med 85:47–54 29328898 10.3949/ccjm.85a.16090

[CR116] Sáenz-Medina J, San Román J, Rodríguez-Monsalve M, Durán M, Carballido J, Prieto D, Gil Miguel Á (2023) Hospitalization burden of patients with kidney stones and metabolic comorbidities in Spain during the period 2017–2020. Metabolites 13:574 37110232 10.3390/metabo13040574 PMC10142441

[CR117] Saigal CS, Joyce G, Timilsina AR, Project UDiA (2005) Direct and indirect costs of nephrolithiasis in an employed population: opportunity for disease management? Kidney Int 68:1808–1814 16164658 10.1111/j.1523-1755.2005.00599.x

[CR118] Sakhaee K (2009) Recent advances in the pathophysiology of nephrolithiasis. Kidney Int 75:585–595 19078968 10.1038/ki.2008.626 PMC3088505

[CR119] Samandarian S, Soltani R, Hajhashemi V, Dehghani M, Matinfar M, Mahboubi M, Mohsenzadeh A (2023) Efficacy of an oral solution containing five herbal extracts in the treatment of urolithiasis: a randomized, single-blind, placebo-controlled clinical trial. J Res Pharm Pract 12:96–103 38716323 10.4103/jrpp.jrpp_11_24 PMC11071061

[CR120] Sayed MA-B, Moeen AM, Saada H, Nassir A, Tayib A, Gadelkareem RA (2022) Mirabegron as a medical expulsive therapy for 5–10 mm distal ureteral stones: a prospective, randomized, comparative study. Turk J Urol 48:209 35634939 10.5152/tud.2022.22014 PMC9730259

[CR121] Schaeffer AJ, Feng Z, Trock BJ, Mathews RI, Neu AM, Gearhart JP, Matlaga BR (2011) Medical comorbidities associated with pediatric kidney stone disease. Urology 77:195–199 20970831 10.1016/j.urology.2010.06.062 PMC4695976

[CR122] Seegmiller JE (1968) Xanthine stone formation. Am J Med 45:780–783 4879836 10.1016/0002-9343(68)90210-6

[CR123] Segall M, Mousavi A, Eisner B, Scotland K (2024) Pharmacologic treatment of kidney stones: current medication and pH monitoring. Actas Urológicas Españolas (English Edition) 48:11–18 10.1016/j.acuroe.2023.11.008 38043680

[CR124] Seker KG, Arikan Y, Seker YC, Ozlu DN, Evren I (2020) An unexpected complication after extracorporeal shock wave lithotripsy: emphysematous pyelitis. Cureus 12(5):e8307 10.7759/cureus.8307 PMC7320654 32607291

[CR125] Setthawong V, Srisubat A, Potisat S, Lojanapiwat B, Pattanittum P (2023) Extracorporeal shock wave lithotripsy (ESWL) versus percutaneous nephrolithotomy (PCNL) or retrograde intrarenal surgery (RIRS) for kidney stones. Cochrane Database Syst Rev 8(8):CD007044 10.1002/14651858.CD007044.pub4 PMC10392035 37526261

[CR126] Shakeri N, Mehrabi S, Mehrabi A, Jahanabad HM (2020) Comparison efficacy of oral Peganum harmala seed versus tamsulosin on pain relief and expulsion of renal and ureteral stones; a randomized clinical trial. J Nephropathol 9(4):e40–e40

[CR127] Shakeri N, Mehrabi S, Paymard A (2021) Comparison efficacy of oral *Nigella sativa* seeds and tamsulosin on pain relief and passage of 4 to 10 mm stones of kidney and ureter; a randomized clinical trial. J Nephropharmacol 11:e8–e8

[CR128] Shastri S, Patel J, Sambandam KK, Lederer ED (2023) Kidney stone pathophysiology, evaluation and management: core curriculum 2023. Am J Kidney Dis 82:617–634 37565942 10.1053/j.ajkd.2023.03.017 PMC11370273

[CR129] Siener R (2021) Nutrition and kidney stone disease. Nutrients 13:1917 34204863 10.3390/nu13061917 PMC8229448

[CR130] Siener R, Metzner C (2023) Dietary weight loss strategies for kidney stone patients. World J Urol 41:1221–1228 36593299 10.1007/s00345-022-04268-w PMC10188387

[CR131] Singh N, Agarwal S, Sarpal R (2024) Prospective evaluation of extracorporeal shockwave lithotripsy in renal and upper ureteric stone treatment: clinical assessment and results. Cureus 16(5):e61102 10.7759/cureus.61102 PMC11128184 38800778

[CR132] Singh P, Harris PC, Sas DJ, Lieske JC (2022) The genetics of kidney stone disease and nephrocalcinosis. Nat Rev Nephrol 18:224–240 34907378 10.1038/s41581-021-00513-4

[CR133] Song H, Liang L, Liu H, Liu Y, Hu W, Zhang G, Xiao B, Fu M, Li J (2024) Mirabegron for medical expulsive therapy of ureteral stones: a systematic review and meta-analysis. Front Med 10:1280487 10.3389/fmed.2023.1280487 PMC10797003 38249979

[CR134] Srivastava T, Schwaderer A (2009) Diagnosis and management of hypercalciuria in children. Curr Opin Pediatr 21:214–219 19307900 10.1097/MOP.0b013e3283223db7

[CR135] Stamatelou KK, Francis ME, Jones CA, Nyberg JLM, Curhan GC (2003) Time trends in reported prevalence of kidney stones in the United States: 1976–1994. Kidney Int 63:1817–1823 12675858 10.1046/j.1523-1755.2003.00917.x

[CR136] Talyshinskii A, Naik N, Hameed BZ, Khairley G, Randhawa P, Somani BK (2024) Telemedicine in endourology for patient management and healthcare delivery: current status and future perspectives. Curr Urol Rep 25:299–310 38980521 10.1007/s11934-024-01224-6 PMC11366724

[CR137] Tanaka Y, Maruyama M, Okada A, Furukawa Y, Momma K, Sugiura Y, Tajiri R, Sawada KP, Tanaka S, Takano K (2021) Multicolor imaging of calcium-binding proteins in human kidney stones for elucidating the effects of proteins on crystal growth. Sci Rep 11:16841 34446727 10.1038/s41598-021-95782-1 PMC8390759

[CR138] Tasian GE, Copelovitch L (2014) Evaluation and medical management of kidney stones in children. J Urol 192:1329–1336 24960469 10.1016/j.juro.2014.04.108

[CR139] Tavichakorntrakool R, Prasongwattana V, Sungkeeree S, Saisud P, Sribenjalux P, Pimratana C, Bovornpadungkitti S, Sriboonlue P, Thongboonkerd V (2012) Extensive characterizations of bacteria isolated from catheterized urine and stone matrices in patients with nephrolithiasis. Nephrol Dial Transplant 27:4125–4130 22461670 10.1093/ndt/gfs057

[CR140] Taylor EN, Stampfer MJ, Curhan GC (2004) Dietary factors and the risk of incident kidney stones in men: new insights after 14 years of follow-up. J Am Soc Nephrol 15:3225–3232 15579526 10.1097/01.ASN.0000146012.44570.20

[CR141] Taylor EN, Stampfer MJ, Curhan GC (2005) Obesity, weight gain, and the risk of kidney stones. JAMA 293:455–462 15671430 10.1001/jama.293.4.455

[CR142] Thorleifsson G, Holm H, Edvardsson V, Walters GB, Styrkarsdottir U, Gudbjartsson DF, Sulem P, Halldorsson BV, de Vegt F, d’Ancona FC (2009) Sequence variants in the CLDN14 gene associate with kidney stones and bone mineral density. Nat Genet 41:926–930 19561606 10.1038/ng.404

[CR143] Ungerer G, Winoker J, Healy K, Shah O, Koo K (2024) Mobile and ehealth technologies in the management and prevention of nephrolithiasis: a systematic review. Actas Urológicas Españolas (English Edition) 48:25–41 10.1016/j.acuroe.2023.06.010 37364768

[CR144] Uribarri J (2020) Chronic kidney disease and kidney stones. Curr Opin Nephrol Hypertens 29:237–242 31972597 10.1097/MNH.0000000000000582

[CR145] Verkoelen C, Verhulst A (2007) Proposed mechanisms in renal tubular crystal retention. Kidney Int 72:13–18 17429341 10.1038/sj.ki.5002272

[CR146] Vitkovskyy VF (2021) Efficacy of an herbal preparation based on lovage, rosemary, and centaury on patients after extracorporal shockwave lithotripsy. Clin Phytoscience 7:1–7

[CR147] Vo TQ, Nguyen TTN, Le TQT, Pham LD, Tran QV (2018) Direct medical costs of kidney stone: a retrospective study. J Clin Diagn Res 12:52–58

[CR148] Wang B, He G, Xu G, Wen J, Yu X (2020) Mirna-34a inhibits cell adhesion by targeting CD44 in human renal epithelial cells: implications for renal stone disease. Urolithiasis 48:109–116 31506763 10.1007/s00240-019-01155-9

[CR149] Wang M, Lin X, Yang X, Yang Y (2022) Research progress on related mechanisms of uric acid activating NLRP3 inflammasome in chronic kidney disease. Ren Fail 44:615–624 35382689 10.1080/0886022X.2022.2036620 PMC9004527

[CR150] Wang Y, Yang J, Amier Y, Yuan D, Xun Y, Yu X (2025) Advancements in nanomedicine for the diagnosis and treatment of kidney stones. Int J Nanomedicine. 10.2147/IJN.S504318 39925679 10.2147/IJN.S504318 PMC11805677

[CR151] Wang Z, Zhang Y, Zhang J, Deng Q, Liang H (2021) Recent advances on the mechanisms of kidney stone formation. Int J Mol Med 48:149 34132361 10.3892/ijmm.2021.4982 PMC8208620

[CR152] Watson G, Payne SR, Kunitsky K, Natchagande G, Mabedi C, Scotland KB (2022) Stone disease in low-and middle-income countries: could augmented reality have a role in its management? BJU Int 130:400–407 35993671 10.1111/bju.15877

[CR153] Xu Z, Yao X, Duan C, Liu H, Xu H (2023) Metabolic changes in kidney stone disease. Front Immunol 14:1142207 37228601 10.3389/fimmu.2023.1142207 PMC10203412

[CR154] Xue X, Liu Z, Li X, Lu J, Wang C, Wang X, Ren W, Sun R, Jia Z, Ji X (2021) The efficacy and safety of citrate mixture vs sodium bicarbonate on urine alkalization in Chinese primary gout patients with benzbromarone: a prospective, randomized controlled study. Rheumatology 60:2661–2671 33211886 10.1093/rheumatology/keaa668 PMC8213434

[CR155] Yohannes P, Smith AD (2002) The endourological management of complications associated with horseshoe kidney. J Urol 168:5–8 12050480

[CR156] Yoon JH, Park S, Kim SC, Park S, Moon KH, Cheon SH, Kwon T (2021) Outcomes of extracorporeal shock wave lithotripsy for ureteral stones according to ESWL intensity. Transl Androl Urol 10:1588 33968647 10.21037/tau-20-1397 PMC8100855

[CR157] Yu J, Yin B (2017) Postmenopausal hormone and the risk of nephrolithiasis: a meta-analysis. EXCLI J 16:986 28900379 10.17179/excli2017-304 PMC5579406

[CR158] Yuan C, Jian Z, Jin X, Ma Y, Li H, Wang K (2021) Efficacy and safety of external physical vibration lithecbole after extracorporeal shock wave lithotripsy or retrograde intrarenal surgery for urinary stone: a systematic review and meta-analysis. J Endourol 35:712–720 32972194 10.1089/end.2020.0820

[CR159] Zheng J, Wang Y, Chen B, Wang H, Liu R, Duan B, Xing J (2020) Risk factors for ureteroscopic lithotripsy: a case-control study and analysis of 385 cases of holmium laser ureterolithotripsy. Videosurg Other Miniinvasive Tech 15:185–191 32117503 10.5114/wiitm.2019.85360 PMC7020703

[CR160] Zhu W, Huang Z, Zeng G (2023) Miniaturization in percutaneous nephrolithotomy: what is new? Asian J Urol 10:275–280 37538153 10.1016/j.ajur.2023.01.003 PMC10394306

[CR161] Ziaeefar P, Basiri A, Zangiabadian M, de la Rosette J, Zargar H, Taheri M, Kashi AH (2023) Medical expulsive therapy for pediatric ureteral stones: a meta-analysis of randomized clinical trials. J Clin Med 12:1410 36835945 10.3390/jcm12041410 PMC9966932

